# Clinical performance of short implants vs. standard implants in edentulous patients. An umbrella review

**DOI:** 10.3389/froh.2025.1670095

**Published:** 2025-09-18

**Authors:** Heber Isac Arbildo-Vega, Fredy Hugo Cruzado-Oliva, Edward Demer Infantes-Ruíz, Sara Antonieta Luján-Valencia, Joan Manuel Meza-Málaga, Consuelo Marroquín-Soto, Franz Tito Coronel-Zubiate

**Affiliations:** 1 Faculty of Dentistry, Dentistry School, Universidad San Martín de Porres, Chiclayo, Peru; 2 Faculty of Human Medicine, Human Medicine School, Universidad San Martín de Porres, Chiclayo, Peru; 3 Faculty of Post Graduate, Universidad Nacional, Toribio Rodríguez de Mendoza de Amazonas, Chachapoyas, Peru; 4 Faculty of Stomatology, Stomatology School, Universidad Nacional de Trujillo, Trujillo, Peru; 5 Faculty of Health Science, Stomatology School, Universidad César Vallejo, Piura, Peru; 6 Postgraduate School, Universidad Católica de Santa María, Arequipa, Peru; 7 Faculty of Dentistry, Dentistry School, Universidad Católica de Santa María, Arequipa, Peru; 8 Department of Dentistry, School of Dentistry, Universidad Científica del Sur, Lima, Peru; 9 Faculty of Health Sciences, Universidad Nacional Toribio Rodríguez de Mendoza de Amazonas, Chachapoyas, Peru

**Keywords:** short implants, standard implants, clinical performance, edentulous patients, review

## Abstract

**Aim:**

To assess the clinical performance of short implants compared to standard-length implants in edentulous patients through an umbrella review.

**Material and methods:**

A comprehensive search was conducted in databases such as PubMed, Cochrane Library, Scopus, SciELO, Google Scholar, ProQuest Dissertations and Theses, and OpenGrey, covering literature up to June 2025. Included studies were systematic reviews, with or without meta-analysis, that compared short implants with standard-length implants, with or without bone augmentation, reporting on implant survival, marginal bone loss, and biological or prosthetic complications. Reviews of a narrative nature, rapid reviews, clinical trials, observational or experimental studies, case reports, editorials, letters, protocols, and posters were excluded. The methodological quality of the reviews was assessed using the AMSTAR-2 tool.

**Results:**

From an initial retrieval of 790 records, 60 systematic reviews met the inclusion criteria. The data showed no significant differences in survival rates, implant failure, or prosthetic complications between short and standard implants. However, short implants showed less marginal bone loss and fewer biological complications.

**Conclusion:**

Based on high-confidence systematic reviews, short implants provide comparable clinical outcomes to standard-length implants and are a viable, less invasive alternative for patients with reduced vertical bone height.

**Systematic Review Registration:**

https://www.crd.york.ac.uk/PROSPERO/view/CRD42020218497, PROSPERO CRD42020218497.

## 1 Introduction

At present, dental implants are considered a dependable therapeutic option for restoring missing teeth in both partially edentulous individuals (1, 2) and those who are completely edentulous (3). Nonetheless, in certain anatomical zones of the oral cavity, limited bone height may hinder the placement of implants with standard dimensions (4).

To address this anatomical limitation, various bone augmentation techniques have been proposed in the literature to improve deficient ridges. These include inlay and onlay grafts (5, 6), sinus floor elevation procedures (7, 8), distraction osteogenesis (9), sandwich osteotomies (10), and guided bone regeneration (11). In the mandibular arch, surgical alternatives such as lateralization or transposition of the inferior alveolar nerve have also been documented (12, 13).

Despite their effectiveness, such interventions are often invasive, technically demanding, and associated with intraoperative or postoperative complications, which may discourage patients from accepting them (14–16). Additionally, these procedures tend to raise the overall cost and extend the treatment duration (17). As a result, short dental implants have emerged as a less invasive, more economical solution, offering satisfactory outcomes, reduced morbidity, and fewer complications in specific clinical scenarios (18–22).

This group of dental implants is supported by several randomized clinical trials (RCTs) (23–25) being compared with standard length implants. However, there are two clinical scenarios for making this comparison: the first is when bone height is limited and there is a need for bone augmentation (23, 24, 26), and the second is when the objective is to compare implants. short and standard under similar conditions (sufficient native bone available for both options) (27–29).

Short implants were initially defined as those with a length of less than 10 mm (18, 30). Other authors have proposed that implants measuring 8 mm or less should be considered as short implants (31), while others have defined them as having a length ≤6 mm (32). Although a consensus on the precise definition remains elusive, a general trend towards shorter implants lengths is evident in the literature (4).

Recent umbrella reviews, such as those by Sáenz-Ravello et al. (33) and Ravidà et al. (34), have contributed valuable insights into the comparison between short and standard implants. However, these reviews present some limitations. Sáenz-Ravello et al. (33) highlighted the lack of consensus on the definition of short implants and noted that many of the reviews included in their analysis had low methodological confidence. Similarly, Ravidà et al. (34) found that implants ≤6 mm may be viable alternatives to longer implants but identified gaps in the evidence regarding long-term outcomes and specific clinical scenarios.

This umbrella review aims to address these gaps by providing a comprehensive synthesis of the most recent and high-quality systematic reviews, considering varying definitions of short implants and assessing the overall reliability and confidence level of the available evidence. We acknowledge that the varying definitions of short implants (from ≤6 mm to ≤10 mm) may influence the outcomes of the studies included. Our review highlights the need for standardized definitions in future research to enhance comparability and clarity in clinical outcomes. Importantly, while several low-confidence reviews were included in our analysis, we conducted a sensitivity analysis to exclude studies with moderate or low methodological quality. By doing so, this review not only updates the existing literature but also offers a more nuanced understanding of the clinical performance of short implants in comparison to standard-length implants.

To provide treatments that are both durable and predictable—while ensuring patient comfort and minimizing complications—clinicians must base their decisions on a robust scientific foundation. The process of consolidating knowledge into a comprehensive resource facilitates this, enabling practitioners to efficiently access, interpret, and apply pertinent information. This process, referred to as knowledge synthesis, entails the organization and integration of individual study findings within a broader context of understanding (35). It acts as a bridge between isolated research outcomes and their practical application, thereby enhancing evidence-based decision-making.

To date, the scientific literature includes only three umbrella systematic reviews comparing the outcomes of short dental implants to those of standard-length implants in combination with either bone augmentation (4, 33) or sinus lifts procedures (16). However, a comprehensive evaluation that synthesizes all existing systematic reviews on this topic—including more recent publications—has yet to be conducted. As a result, this umbrella review seeks to consolidate and interpret the current body of evidence to address the following key question: What is the clinical effectiveness of short implants in comparison to standard-length implants? Additionally, this review seeks to assess the overall reliability and confidence level of the systematic reviews available on this topic.

## 2 Materials and methods

### 2.1 Protocol and registration

This umbrella review was conducted following the Preferred Reporting Items for Systematic Review and Meta-Analysis Protocols (PRISMA-P) guidelines (36), and was registered in the Prospective Register of Systematic Reviews (PROSPERO) under the ID number CRD 42020218497 (https://www.crd.york.ac.uk/PROSPERO/view/CRD42020218497) (37), which is publicly accessible. Furthermore, the reporting of this review adhered to the PRIO-harms checklist (Preferred Reporting Items for Overviews of Systematic Reviews) (38). Given the nature of the study, ethical approval was not required.

### 2.2 Eligibility criteria and results of interest

Studies eligible for inclusion were systematic reviews (with or without meta-analysis) assessing primary research comparing dental implants of different lengths, with or without concurrent bone augmentation procedures. Given the variability and lack of consensus in the literature regarding the definition of short implants, and following prior studies that investigated clinical outcomes according to implant length (4, 8, 19, 33, 34), we adopted a pragmatic classification system, hereafter referred to as the “Proposed Implant Length Classification”, to facilitate synthesis, interpretation and reproducibility. This classification defines implants as follows:

- Conventional (or long) implants: length ≥10 mm
- Intermediate (or medium) implants: length >8 mm and <10 mm (infrequently reported in the literature)
- Short implants: length ≤8 mm
- Ultrashort implants: length ≤6 mm

This classification was informed by the distribution of implant lengths reported in the included systematic reviews, which are summarized in Supplementary Material 6. Although this Supplementary Material reflects extracted study data and thus comes from results already obtained, presenting it after the main methodological materials (1–5) maintains the natural sequence of supplements while transparently linking the classification framework to the evidence.

While some studies compare implants with overlapping length categories (e.g., 6 mm vs. 8 mm), this classification provides a standardized operational framework to interpret outcomes such as implant survival rates, marginal bone loss, and biological and/or prosthetic complications. The inclusion of an intermediate category ensures completeness and transparency, even if few studies specifically report results in this range, and allows future research to adopt this framework for better comparability.

No restrictions were applied regarding publication date or language. Excluded publication types were narrative reviews, rapid reviews, interventional studies, observational research, preclinical and basic science studies, protocols, abstracts, case reports, commentaries, letters, opinions, and poster presentations.

### 2.3 Sources of information, search strategy and additional search for primary studies

An electronic literature search was conducted on June 20, 2025, using four major databases: PubMed, Cochrane Library, Scopus, and SciELO. To identify additional records, gray literature sources were also consulted, including Google Scholar, ProQuest Dissertations and Theses, and OpenGrey. Reference lists of included studies were manually screened to identify any relevant additional publications. All retrieved articles were imported into Zotero® (Center for History and New Media, Virginia, USA), and duplicates were removed. Detailed search strategies for each database are presented in Supplementary Material S1.

### 2.4 Data management and selection process

The screening and selection process was carried out using Rayyan® online software (Qatar Computing Research Institute, Qatar). Study selection was conducted in two phases. In the first phase, two independent reviewers (F.C.O. and E.I.) assessed titles and abstracts. In the second phase, the full texts of potentially relevant studies were evaluated independently by the same reviewers. Disagreements at any stage were resolved through discussion with a third reviewer (H.A.).

### 2.5 Data collection process

Data extraction was carried out independently and in duplicate by two reviewers (F.C.O. and C.CH.) using a standardized data collection form. Extracted information was cross-checked for consistency, and disagreements were resolved by a third author (H.A.). The following variables were recorded: author names, year of publication, type of systematic review, characteristics of included primary studies, number of studies included in qualitative and quantitative analyses, intervention and comparator details, implant placement region, treatment conditions (e.g., with or without bone augmentation or sinus lift), reported outcomes, main conclusions, and whether the reviews reported adherence to PRISMA guidelines, PROSPERO registration, use of the GRADE system, and performance of a meta-analysis.

### 2.6 Assessment of methodological quality, quality of evidence and meta-bias

The methodological quality of the included systematic reviews was assessed independently and in duplicate by two reviewers (F.C.Z. and S.L.V.), calibrated (Kappa 0.85), using the AMSTAR-2 checklist (A MeaSurement Tool to Assess Systemic Reviews) (39). The overall confidence level in the studies was rated as high, moderate, low, or critically low. To assess meta-bias or the risk of bias in the systematic reviews, we adopted a sensitivity analysis approach, excluding studies with moderate or low methodological quality. While the ROBIS tool is recommended for assessing risk of bias in umbrella reviews, we chose not to use it in this analysis due to its complexity in handling multiple reviews with varying methodologies. Instead, we relied on AMSTAR-2, which provided a more streamlined and consistent evaluation of the included systematic reviews.

### 2.7 Summary of measures

For systematic reviews (SRs) that did not include a meta-analysis, the extracted outcomes were reported in millimeters for marginal bone loss and in percentages for implant survival, implant failure, biological and prosthetic complications, as well as perioperative, intraoperative, and postoperative events. In cases where the SRs provided a meta-analysis, the results were recorded using either mean difference or standardized mean difference for marginal bone loss. For implant survival and other complications (biological, prosthetic, intraoperative, perioperative, and postoperative), effect estimates such as relative risk (RR), odds ratio (OR), or risk difference (RD) were included.

### 2.8 Summary of results

The primary findings from the included systematic reviews were organized and reported according to key clinical outcomes, including marginal bone loss, implant survival and failure, biological complications, prosthetic complications, and complications occurring during the perioperative, intraoperative, or postoperative periods.

## 3 Results

### 3.1 Review and selection of primary studies

**Figure 1:**
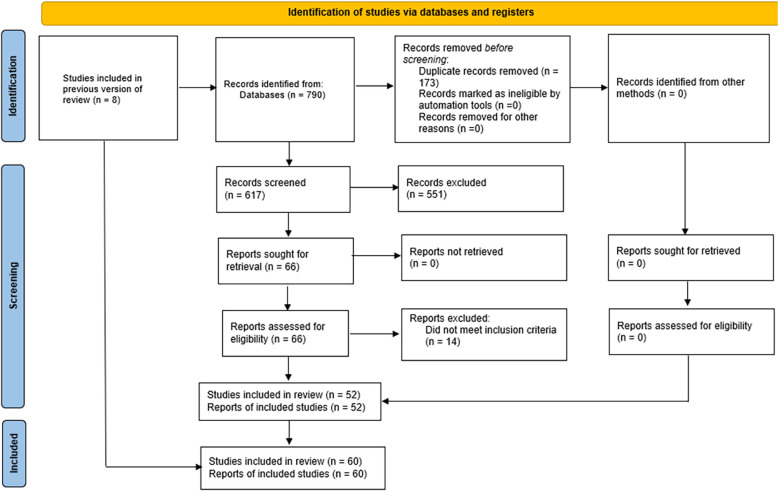
PRISMA flow diagram showing the selection process of studies included in the systematic review, from initial identification to final inclusion.

A total of 790 records were identified through the electronic database search. After eliminating duplicates, 617 unique references remained. In the initial screening phase, titles and abstracts were reviewed, resulting in 51 studies deemed suitable for full-text assessment. Additionally, nine more articles were identified through manual searches within other umbrella reviews, bringing the total to 60 systematic reviews included in the qualitative synthesis. Details regarding the exclusion criteria applied during the selection process are provided in Supplementary Material S2. The complete workflow for study identification and selection is illustrated in Figure 1.

### 3.2 Review and characteristics of included studies

The included systematic reviews (SRs) were published between 2009 and 2025. Of these, only one was published in Chinese; the remainder were in English. The studies originated from a diverse range of countries, including Puerto Rico (40), Lithuania (41, 42), China (43–55), Greece (56, 57), Iran (58, 59), Romania (60), Italy (15, 61–65), Germany (66, 67), India (14, 68), Spain (69–72), Brazil (18, 73–78), Sweden (79, 80), the United States (32, 81–86), Tunisia (87), Denmark (88, 89), Egypt (90), Morocco (91), Saudi Arabia (92), Switzerland (93), and France (94). Further details regarding the characteristics of the SRs are provided in Supplementary Material S3.

### 3.3 Assessment of methodological quality and quality of evidence

23 SRs (15, 43–45, 47–49, 53, 58, 59, 61–63, 68, 69, 73–75, 82, 83, 89, 91) were considered to have high confidence, 1 SR (52) to have moderate confidence, 27 SRs (18, 32, 40, 41, 44, 46, 50, 51, 54, 56, 57, 60, 64, 65, 70, 71, 76, 77, 79, 80, 84, 85, 87, 88, 90, 93, 94) they had low confidence and 9 SRs (42, 55, 66, 67, 72, 78, 81, 86, 92) had critically low confidence (Supplementary Material S4).

The included systematic reviews were assessed for methodological quality using the AMSTAR-2 tool, which rated studies as having high, moderate, low, or critically low confidence. To mitigate the impact of low-confidence reviews on our overall findings, a sensitivity analysis was conducted, excluding studies with moderate or low confidence. The results presented here are based exclusively on high-confidence reviews, ensuring the reliability of our conclusions.

### 3.4 Overlapping

A total of 1,030 primary studies were identified within the SRs. The degree of overlap according to the CCA index is 1.98%, and this value indicates “slight overlap”. Specifically, 31 studies were included twice, 12 appeared three times, 8 were included four times, 4 appeared in five SRs, and 6 studies were included six times. Additionally, 3 studies were cited in eight SRs, 5 in 9, 2 in 10, and 3 in 12. Four studies were included 14 times, 2 appeared in 15, 4 in 16, and 2 in 17 SRs. Notably, one study was included 19, 20, 24, 26, 27, and 28 times. Further details on the degree of overlap and characteristics of the primary studies are provided in Supplementary Material S5.

### 3.5 Synthesis of results

The synthesis of the results is presented in Supplementary Material S3.

In the analysis of implant survival rates, marginal bone loss, biological complications, and prosthetic complications, we further categorized these outcomes based on the type of implant (short vs. standard) and whether bone augmentation was performed. This allowed for a more precise comparison of the clinical outcomes across different clinical scenarios.

### 3.6 Survival rate

Thirty-six SRs (14, 32, 40, 44, 45, 47, 48, 52–54, 57, 60–64, 66, 67, 69–71, 73–78, 82, 84, 87–89, 93, 94) included reported no differences in the survival rate of short implants compared to standard implants, while 3 SRs (56, 68, 92) reported that standard implants had a higher survival rate and 5 SRs (41, 50, 51, 83, 86) reported that short implants short implants had a higher survival rate. Thirty-eight SRs (14, 32, 40, 44, 45, 47, 48, 50–54, 56, 57, 60, 62–64, 66–71, 73–78, 82–86, 88, 92) meta-analyzed the results, where they found that the relative risk ratio ranged from 0.68 (CI: 0.24–1.93) (84) to 3.28 (CI: 0.94–11.50) (66), the odds ratio ranged from 0.90 (CI: 0.15–5.44) (88) to 1.42 (CI: 0.21–9.63) (44) and the risk difference ranged from −0.02 (CI: −0.04 to 0.00) (74) to 0.05 (CI: 0.03–0.07) (56). Carosi et al. (15) presented the results descriptively and reported a survival of 92%–96.9% for short implants and 84.8%–100% for standard implants. Starch-Jensen et al. (89) reported a survival of 91.7% for short implants and 95.1% for standard implants. Thoma et al. (24) found a 99% survival for short implants and 99.5% for standard implants. Nisand et al. (94) indicated a survival of 96.24% for short implants and 95.09% for standard implants. Rosa et al. (61) determined an average survival of 94.2%–97.4% for short implants and standard implants. Abayov et al. (41) determined an average survival of 93.91%–91.83% for short implants and standard implants.

### 3.7 Implant failure

Eleven systematic reviews (42, 43, 46, 49, 55, 58, 59, 65, 72, 80, 90) reported no significant difference in failure rates between short and standard implants. In contrast, two SRs (81, 83) found lower failure rates for standard implants, whereas one SR (18) indicated better outcomes with short implants. All reviews conducted meta-analyses. Reported relative risk ratios ranged from 0.78 (CI: 0.10–5.16) (55) to 3.64 (CI: 0.91–14.53) (46); odds ratios varied between 1.02 (CI: 0.31–3.31) (72) and 1.38 (CI: 0.67–2.84) (49); and risk differences ranged from −0.05 (CI: −0.19 to 0.09) (90) to 0.06 (CI: 0.04–0.10) (18).

### 3.8 Marginal bone loss

Fifteen systematic reviews (44, 47, 51, 56, 61, 62, 71, 75, 77, 85, 90, 91, 93, 94) reported no significant difference in marginal bone loss between short and standard implants, whereas 31 SRs (14, 15, 18, 40–43, 45, 46, 48, 50, 52, 53, 55, 58–60, 64–70, 73, 74, 80, 82, 83, 88, 89) found that short implants were associated with reduced marginal bone loss. A total of 40 SRs (18, 40–48, 50–53, 55, 56, 58–60, 62, 64–71, 73–75, 77, 80, 82, 83, 85, 88, 90) conducted meta-analyses, with mean differences ranging from −0.88 mm (CI: −1.26 to −0.50) (67) to 0.86 mm (CI: 0.75–0.98) (70), and standardized mean differences from −0.51 (CI: −0.93 to −0.10) (41) to −0.09 (CI: −0.18 to 0.01) (56). Descriptive data from individual SRs further supported these findings: Carosi et al. (15) reported marginal bone loss values of −0.51 to −2.30 mm for short implants and −0.77 to −2.64 mm for standard implants; Amine et al. (91) observed a loss ranging from −0.1 to −1.49 mm for short implants and −0.1 to −2.34 mm for standard implants; Starch-Jensen et al. (89) reported losses of −2.24 mm and −3.01 mm for short and standard implants, respectively; Thoma et al. (24) found marginal bone loss ranging from −0.1 to −1.02 mm for short implants and −0.1 to −1.15 mm for standard implants; Nisand et al. (95) observed losses of −1.23 mm in short implants and −1.51 mm in standard implants; and Rosa et al. (61) found a mean marginal bone loss of 0.12 mm in both groups.

### 3.9 Biological complications

Eigth SRs (43, 46, 53, 61, 62, 82–84) included reported that there were no differences in the presence of biological complications of short implants compared to standard implants, while 14 SRs (14, 18, 45, 48, 50, 58–60, 65, 70, 73, 75, 80, 93) reported that short implants had fewer biological complications. Twenty SRs (14, 18, 43, 45, 46, 48, 50, 53, 58–60, 62, 65, 70, 73, 75, 80, 82–84) meta-analyzed the results, where they found that the risk ratio relative ranged from 0.21 (CI: 0.10–0.41) (76) to 4.72 (CI: 2.43–9.17) (80), the odds ratio was 0.47 (CI: 0.19–1.18) (43), and the risk difference ranged from −0.07 (CI: −0.11 to −0.04) (58) to 0.04 (CI: 0.02–0.08) (18). Thoma et al. (24) presented the results descriptively and reported a presence of biological complications of 2.94% for short implants and 8.84% for standard implants. Rosa et al. (61) determined an average presence of biological complications of 0%–11.1% for short implants and standard implants.

### 3.10 Prosthetic complications

Twenty systematic reviews (14, 15, 43, 48, 53, 55, 56, 58–61, 65, 70–73, 77, 80, 83, 84) reported no significant difference in the incidence of prosthetic complications between short and standard implants, while seven SRs (45, 46, 50, 62, 75, 82, 93) concluded that standard implants were associated with fewer prosthetic complications, and two SRs (18, 94) found a lower frequency of such complications in short implants. Meta-analyses were performed in 25 SRs (14, 18, 43, 45, 46, 48, 50, 53, 55, 56, 58–60, 62, 65, 70–73, 75, 77, 80, 82–84), where the relative risk ratios ranged from 0.43 (CI: 0.13–1.43) (73) to 3.15 (CI: 1.32–7.51) (75), the odds ratios varied from 0.64 (CI: 0.21–1.96) (72) to 0.94 (CI: 0.45–1.94) (43), and the risk differences ranged from 0.0 (CI: −0.01 to 0.01) (58) to 0.03 (CI: 0.02–0.06) (18). Descriptive data reported by Carosi et al. (15) showed prosthetic complication rates ranging from 0% to 9.1% for short implants and from 0% to 10% for standard implants; Thoma et al. (24) found a complication rate of 1.98% in short implants and 1.4% in standard ones; Nisand et al. (94) reported values of 3.68% and 5.45% for short and standard implants, respectively; and Rosa et al. (61) observed an average complication rate of 31.8% in both types of implants.

### 3.11 Complication rate

Five SRs (40, 52, 64, 77, 92) included reported no difference in the complication rate of short implants compared to standard implants, while 5 SRs (54, 55, 78, 87, 94) reported that short implants had a lower complication rate. Nine SRs (40, 52, 54, 55, 64, 77, 79, 92) meta-analyzed the results, where they found that the relative risk ratio ranged from 0.17 (CI: 0.04–0.73) (78) to 0.88 (CI: 0.64–1.21) (40). Nisand et al. (94) presented the results descriptively and reported a complication rate of 14.11% for short implants and 38.79% for standard implants.

### 3.12 Intra-operative complications

Two systematic reviews (47, 70) reported no significant difference in the incidence of intraoperative complications between short and standard implants. Both SRs conducted meta-analyses, with relative risk ratios ranging from 0.51 (CI: 0.16–1.63) (72) to 1.14 (CI: 0.46–2.83) (47).

### 3.13 Perioperative complications

One SR (66) included reported that there were no differences in the presence of perioperative complications of short implants compared to standard implants, while 1 SR (67) reported that short implants had fewer perioperative complications operative. These 2 studies meta-analyzed the results, finding that the relative risk ratio ranged from 0.33 (CI: 0.09–1.16) (66) to 0.34 (CI: 0.19–0.60) (67).

### 3.14 Post-operative complications

Four systematic reviews (42, 47, 70, 90) reported no significant differences in the occurrence of postoperative complications between short and standard implants, while two SRs (72, 78) found a lower incidence of such complications associated with short implants. All six SRs conducted meta-analyses, reporting relative risk ratios ranging from 0.22 (CI: 0.07–0.71) (78) to 1.34 (CI: 0.71–2.55) (47), an odds ratio of 0.12 (CI: 0.05–0.26) (72), and risk differences ranging from −0.39 (CI: −0.92 to −0.14) (42) to −0.27 (CI: −0.89 to −0.35) (90).

## 4 Discussion

In recent years, there has been growing interest in evaluating the clinical performance of short dental implants compared to standard-length implants placed in sites with or without bone augmentation. Numerous randomized controlled trials (RCTs) and systematic reviews (SRs) have provided evidence in favor of short implants; However, their clinical effectiveness can be influenced by varying clinical conditions. Some studies have focused on cases with limited vertical bone availability, while others have evaluated outcomes in patients with sufficient native bone to accommodate either implant type. In scenarios of inadequate bone height, a range of augmentation techniques has been proposed to recover lost dimensions; However, these approaches often involve higher risks of intraoperative or postoperative complications, increased financial burden, and extended treatment times—factors that may lead patients to reject them. As a result, short implants have emerged as a viable alternative in these cases (4). A systematic review conducted during the sixth ITI Consensus Conference (32) addressed both of these clinical scenarios in a unified analysis. Meanwhile, other SRs have approached the comparison differently, analyzing the performance of short implants vs. standard-length implants combined with augmentation procedures in a separated manner. These differing methodologies emphasize the importance of synthesizing the available findings and critically evaluating the methodological quality of the reviews addressing this topic.

Two previously conducted umbrella reviews analyzing the performance of short vs. standard dental implants in cases requiring bone augmentation (4, 16) have reported that, in terms of survival rate, no significant differences were observed between the two types of implants. Regarding marginal bone loss, the findings favored short implants, indicating better performance in this parameter. When biological complications were evaluated, the results also leaned toward short implants. However, with respect to prosthetic complications, both groups showed similar outcomes, although certain systematic reviews cited in these umbrella reviews did report a lower incidence of such complications in standard implants.

This umbrella review involved an extensive literature search aimed at identifying and synthesizing systematic reviews (SRs) that compared short dental implants to standard implants, with or without accompanying bone augmentation procedures. A total of 60 SRs met the predefined inclusion criteria and were included for detailed analysis. However, a sensitivity analysis was performed to ensure that our conclusions were based on high-confidence reviews only, excluding those with low or moderate methodological quality. This helped mitigate the impact of low-confidence studies on our overall findings. Given the lack of a universally accepted definition for short implants and the variability in definitions across included systematic reviews, we adopted a pragmatic classification for this review, herein referred to as the “Proposed Implant Length Classification.” This classification groups implants as conventional (≥10 mm), intermediate (>8 mm and <10 mm), short (≤8 mm), and ultrashort (≤6 mm). This operational classification facilitated a more standardized synthesis and interpretation of clinical outcomes, while acknowledging the inherent overlap and heterogeneity among studies comparing implants of varying lengths. The development of this classification was informed by the distribution of implant lengths extracted from the included systematic reviews, which are summarized in Supplementary Material S6. Despite the fact that this Supplementary Material represents data already obtained from the included SRs, presenting it as SM6 allows readers to understand the evidence base that directly informed the proposed length categories, maintaining transparency and reproducibility. Although SRs are considered the highest level in the hierarchy of scientific evidence, they are not immune to potential sources of bias, and their findings must be interpreted with critical consideration. The SRs analyzed in this review presented several limitations stemming from the characteristics of the included primary studies, such as variability in study designs, types of implant systems, surface treatments, prosthetic platforms, implant–abutment connections, soft tissue thickness, follow-up durations, implant placement sites, bone quality, prosthetic protocols, types of bone grafts used, and the surgical techniques employed.

A key limitation of this umbrella review is the heterogeneity in implant length definitions used across the included systematic reviews. Although we proposed the “Proposed Implant Length Classification” as an operational framework to standardize grouping—defining conventional implants as ≥10 mm, short implants as ≤8 mm, and ultrashort implants as ≤6 mm—some studies compared implants with overlapping or intermediate length categories (e.g., 6 mm vs. 8 mm). This variability introduces potential inconsistencies in the pooled results and highlights the lack of a universally accepted classification in the field. Consequently, our findings should be interpreted considering this inherent heterogeneity. Additionally, the proposed classification provides a standardized framework that may inform future consensus efforts on defining implant lengths. By offering clear operational categories—conventional, intermediate, short, and ultrashort—this approach can facilitate consistent reporting, enable comparative analyzes across studies, and support evidence-based clinical decision-making in implant dentistry. Future research would benefit greatly from the adoption of standardized implant length definitions to improve comparability and the strength of evidence synthesis.

Some of the systematic reviews included in this umbrella analysis exhibited a high level of confidence, which may enhance the overall quality of evidence and the reliability of the conclusions drawn. However, the continued presence of SRs with low or moderate confidence levels highlights the ongoing need to improve the methodological rigor in studies addressing this topic. The assessment of methodological quality was conducted using the AMSTAR-2 tool, a current and validated instrument for evaluating SRs. Particular attention must be paid to the critical domains 2, 4, 9, and 13 of AMSTAR-2, as several reviews failed to explicitly detail their methodologies, did not apply comprehensive search strategies, lacked adequate techniques for assessing risk of bias, and did not integrate the risk of bias into the interpretation or discussion of their results. These shortcomings emphasize the importance of incorporating these methodological elements in future systematic reviews. Additionally, a frequently unmet criterion among the included SRs was the reporting of publication bias. Similar findings were reported by Koletsi et al. (95), who also noted that publication bias is often overlooked or inadequately assessed in meta-analyses related to oral health research.

Although numerous systematic reviews have examined this specific topic, the interpretation of their findings should be approached with caution, as more than half of the primary studies were included in multiple reviews. This repeated inclusion may result in redundant assessments of the same data, potentially creating an inflated sense of the available evidence. While the development of new systematic reviews could help address certain methodological limitations, as highlighted by Moher (96), the high degree of overlap suggests that future efforts should prioritize the design and execution of robust randomized controlled trials (RCTs), conducted by independent research groups and incorporating long-term follow-up, to strengthen and diversify the current evidence base.

### 4.1 Evidence summary

This umbrella review was conducted with the aim of supporting clinical decision-making concerning the effectiveness of short vs. standard dental implants in edentulous patients. The objective was to reduce potential biases and random errors commonly encountered in systematic reviews and meta-analyses addressing this topic. Despite the limitations observed among the SRs included in this study, it remains possible to synthesize and critically discuss the key findings derived from the available evidence.

The systematic reviews analyzed in this study indicated that both short and standard implants exhibit comparable rates of survival and failure. These findings are consistent with those reported by Felice et al. (26), whose randomized controlled trial with an 8-year follow-up showed similar outcomes. Nonetheless, the ITI Consensus Report (97), referencing a separate RCT with a 5-year follow-up (27), suggests that the duration of functional loading might negatively impact the long-term survival of short implants when compared to longer ones.

With respect to marginal bone loss, short implants demonstrated better performance compared to standard implants. Although this outcome may be partially attributed to the placement of short implants in native bone rather than in sites with bone augmentation (98), the available evidence on this distinction remains limited. Nevertheless, current literature indicates that marginal bone loss around implants is influenced by multiple variables, such as soft tissue thickness (99), implant positioning (100), the type of implant–abutment connection (101), and the number of implants involved in the prosthetic rehabilitation (67).

Biological complications appeared more frequently in the group receiving standard implants, which may be attributed to their placement in conjunction with bone grafting procedures. We further categorized biological complications into mucositis, peri-implantitis, and implant failure to provide more detailed comparisons between short and standard implants. This finding aligns with the 2018 ITI Consensus Report, which indicated that the incidence of surgical and postoperative complications tends to be higher in patients treated with standard implants compared to those receiving short implants. Most of these complications were associated specifically with the bone grafting techniques used during implant placement (97).

Biological complications may arise over time such as mucositis and peri-implantitis and can even progress to implant failure; However, they may also originate as immediate consequences of the surgical intervention. Furthermore, the effectiveness of peri-implantitis treatment may differ between standard and short implants, particularly due to the rapid progression of peri-implant diseases (102), posing a significant clinical challenge in cases involving short implants. This is especially relevant since resective surgical approaches are contraindicated for short and ultra-short implants when managing peri-implantitis (103). Additionally, future studies should aim to better standardize the definition of biological complications, clearly distinguishing between those that occur during or after surgery and those that emerge following prosthetic loading.

Most of the systematic reviews included in this analysis reported no significant differences between short and standard implants regarding prosthetic complications. However, some reviews indicated a greater incidence of specific issues such as crown fractures and screw loosening within the short implant group. To accurately assess the extent of these findings, future studies should provide more detailed reporting of such complications. Concerning the overall complication rate, the SRs generally suggested that short implants were associated with fewer complications. Nevertheless, the lack of differentiation between biological and prosthetic complications limits a comprehensive understanding of this outcome.

The systematic reviews included in this analysis indicated generally no significant differences between short and standard implants in terms of intraoperative, perioperative, and postoperative complications. However, given the limited number of studies that categorized complications in this manner, it is recommended that future research clearly classify these events as either biological or prosthetic in nature to allow for more accurate interpretation and comparison.

### 4.2 Implications for clinical practice

Short dental implants may offer a feasible, simpler, and less invasive alternative, particularly in cases with limited vertical bone availability. Given their reduced surgical invasiveness, they can be particularly beneficial in elderly patients or those with significant comorbidities, where minimizing surgical risk is a priority. Additionally, their use can reduce the need for costly and complex bone augmentation procedures, which may enhance patient acceptance and satisfaction. Their use, supported by a comprehensive clinical diagnosis and treatment planning, can reduce patient morbidity, shorten treatment time, and minimize the need for bone augmentation procedures. This makes them a valuable option in clinical scenarios where traditional implant placement may be more invasive, costly, or time-consuming. Given the reduced risk of complications and the potential for faster recovery, short implants could enhance patient satisfaction by providing a less complex treatment pathway.

### 4.3 Implications for research

Despite the large number of existing systematic reviews, this review highlights the critical need for improved reporting quality and methodological rigor. Future systematic reviews should adhere strictly to structured guidelines and use comprehensive methodological quality assessment tools throughout their development. Furthermore, it is crucial that future studies adopt standardized definitions of short implants to improve consistency and comparability of outcomes. Long-term randomized controlled trials with larger sample sizes are necessary to assess the effectiveness and safety of short implants across various clinical scenarios. Primary studies, particularly randomized controlled trials (RCTs), must be conducted with high methodological standards to generate reliable and robust outcomes. These studies should include precise descriptions of the implant systems used, detailed reporting on types of failure (particularly biological and prosthetic), and longer-term follow-up to assess the durability of outcomes. Furthermore, future research should prioritize patient-reported outcomes, which remain underexplored, and focus on diverse patient populations across different clinical settings to ensure the generalizability of the findings.

## 5 Conclusions

Based on the results and conclusions of the systematic reviews with high methodological confidence, short implants demonstrate clinical outcomes comparable to those of standard-length implants. These results suggests that short implants are a viable and less invasive alternative, particularly in areas with limited bone height. They offer a promising solution for patients who may not be candidates for traditional implants, providing comparable survival rates and marginal bone loss, short implants could become the preferred choice in certain clinical scenarios, reducing patient morbidity and shortening treatment time. However, further randomized controlled trials (RCTs) with long-term follow-up are needed to reinforce these findings and provide more robust evidence for their clinical application.

## Data Availability

The original contributions presented in the study are included in the article/Supplementary Material, further inquiries can be directed to the corresponding author.

## Supplementary material

Supplementary Material S1 Database search strategy—a detailed description of the search terms and strategies used for each database.

Supplementary Material S2 Reason for exclusion of studies—a list of studies that were excluded from the review with reasons for their exclusion.

Supplementary Material S3 Characteristics of included studies—a table summarizing the characteristics of the systematic reviews included in the analysis.

Supplementary Material S4 Assessment of the methodological quality and the quality of the evidence of the included studies—a summary of the quality assessments for the included systematic reviews using the AMSTAR-2 tool.

Supplementary Material S5 Overlap of primary studies in systematic reviews—an overview of the overlap of primary studies across the included systematic reviews.

Supplementary Material S6 Implant lengths compared across included systematic reviews—a table summarizing the number of studies, main authors, implant lengths compared, and clinical outcomes.
