# Supplementary material for: Clinical performance of short implants vs. standard implants in edentulous patients. An umbrella review
Source: Front Oral Health. 2025 Sep 18;6:1670095. doi: 10.3389/froh.2025.1670095 (PMC12488567; doi:10.3389/froh.2025.1670095)
Supplement: Supplementary Material S1 — Database search strategy—a detailed description of the search terms and strategies used for each database. [file Table1.docx]

Supplementary Material 1. Database search strategy

| **Database** | **Search strategy** | **Number of studies** |
| --- | --- | --- |
| Pubmed | (("short implant") OR ("extra short implant") OR ("ultrashort implant") OR ("short dental implant") OR ("reduced implant length") OR ("regular implant")) AND (("long implant") OR ("standard implant") OR ("longer implant")) AND (("systematic review") OR ("meta-analysis")) | 9 |
| Cochrane database | #1 MeSH descriptor: [Dental Implants] explode all trees  #2 ("short implant"):ti,ab,kw OR ("extra short implant"):ti,ab,kw OR ("ultrashort implant"):ti,ab,kw OR ("super short implant"):ti,ab,kw OR ("Short dental implant"):ti,ab,kw (Word variations have been searched)  #3 ("5 mm dental implant"):ti,ab,kw OR ("6 mm dental implant"):ti,ab,kw OR ("7 mm dental implant"):ti,ab,kw OR ("reduced implant length"):ti,ab,kw OR ("regular implant"):ti,ab,kw (Word variations have been searched)  #4 #1 OR #2 OR #3  #5 ("long implant"):ti,ab,kw OR ("long dental implant"):ti,ab,kw OR ("Regular length implant"):ti,ab,kw OR ("Standard implant"):ti,ab,kw OR ("Longer implant"):ti,ab,kw (Word variations have been searched)  #6 #1 OR #5  #7 MeSH descriptor: [Systematic Review] explode all trees  #8 MeSH descriptor: [Meta-Analysis as Topic] explode all trees  #9 ("systematic review"):ti,ab,kw OR ("meta analysis"):ti,ab,kw OR ("meta-analysis"):ti,ab,kw (Word variations have been searched)  #10 #7 OR #8 OR #9  #11 #4 AND #6 AND #10 | 3 |
| Scielo | (short implant) AND (longer implant) AND (systematic review) | 1 |
| Scopus | (TITLE-ABS-KEY ((("short implant") OR ("extra short implant") OR ("ultrashort implant") OR ("super short implant") OR ("short dental implant") OR ("5 mm dental implant") OR ("6 mm dental implant") OR ("7 mm dental implant") OR ("reduced implant length") OR ("regular implant"))) AND TITLE-ABS-KEY ((("long implant") OR ("long dental implant") OR ("regular length implant") OR ("standard implant") OR ("longer implant"))) AND TITLE-ABS-KEY ((("systematic review") OR ("meta-analysis")))) | 68 |
| Google Scholar | (("short implant") OR ("short dental implant")) + (("long implant") OR ("long dental implant") OR ("Standard implant") OR ("Longer implant")) + (("Systematic review") OR ("Meta-analysis")) | 573 |
| Proquest Dissertations and Theses | (("short implant") OR ("short dental implant")) AND (("long implant") OR ("long dental implant") OR ("Standard implant") OR ("Longer implant")) AND (("Systematic review") OR ("Meta-analysis")) | 136 |
| OpenGrey | (("short implant") OR ("short dental implant")) AND (("long implant") OR ("long dental implant") OR ("Standard implant") OR ("Longer implant")) AND (("Systematic review") OR ("Meta-analysis")) | 0 |
