# Supplementary material for: Clinical performance of short implants vs. standard implants in edentulous patients. An umbrella review
Source: Front Oral Health. 2025 Sep 18;6:1670095. doi: 10.3389/froh.2025.1670095 (PMC12488567; doi:10.3389/froh.2025.1670095)
Supplement: Supplementary Material S2 — Reason for exclusion of studies—a list of studies that were excluded from the review with reasons for their exclusion. [file Table2.docx]

Supplementary Material 2. Reason for exclusion of studies

| **Author** | **Reason for exclusion** |
| --- | --- |
| Menchero – Cantalejo et al. (1) | Not compared with standard implants |
| Telleman et al. (2) |  |
| Sun et al. (3) |  |
| Atieh et al. (4) |  |
| Karthikeyan et al. (5) |  |
| Annibali et al. (6) |  |
| Santos et al. (7) |  |
| Ravidà et al. (8) |  |
| Michel et al. (9) |  |
| Barause et al. (10) | Not compared with short implants |
| Mugri (11) |  |
| Kadkhodazadeh et al. (12) |  |
| Shi et al. (13) |  |
| Yang et al. (14) | Does not define the lengths of short and standard implants |

**References:**

1. Menchero-Cantalejo E, Barona-Dorado C, Cantero-Álvarez M, Fernández-Cáliz F, Martínez-González JM. Meta-analysis on the survival of short implants. *Med Oral Patol Oral Cirugia Bucal*. 2011;16(4):e546-51. doi:10.4317/medoral.16.e546

2. Telleman G, Raghoebar GM, Vissink A, den Hartog L, Huddleston Slater JJR, Meijer HJA. A systematic review of the prognosis of short (<10 mm) dental implants placed in the partially edentulous patient. *J Clin Periodontol.* 2011;38(7):667-76. doi:10.1111/j.1600-051X.2011.01736.x

3. Sun HL, Huang C, Wu YR, Shi B. Failure rates of short (≤ 10 mm) dental implants and factors influencing their failure: A systematic review. *Int J Oral Maxillofac Implants*. 2011;26(4):816-25.

4. Atieh MA, Zadeh H, Stanford CM, Cooper LF. Survival of short dental implants for treatment of posterior partial edentulism: A systematic review. *Int J Oral Maxillofac Implants.* 2012;27(6):1323-31.

5. Desai S, Karthikeyan I, Singh R. Short implants: A systematic review. *J Indian Soc Periodontol*. 2012;16(3):302-12. doi:10.4103/0972-124X.100901

6. Annibali S, Cristalli MP, Dell’Aquila D, Bignozzi I, La Monaca G, Pilloni A. Short Dental Implants: A Systematic Review. *J Dent Res.* 2012;91(1):25-32. doi:10.1177/0022034511425675

7. Santos MHB, Bovo PJ de LB, Magalhães HE, Neves LR, Kassis EN. Short Dental Implants: State of the Art and Systematic Review. *MedNEXT J Med Health Sci.* 2021;2(3):50-5. doi:10.34256/mdnt2138

8. Ravidà A, Galli M, Bianchi M, Parisi E, Saleh M, Stacchi C, et al. Clinical outcomes of short implants (≤ 6 mm) placed between two adjacent teeth/implants or in the most distal position: A systematic review and meta-analysis. *Eur J Oral Implantol*. 2021;14(3):241-57.

9. Michel RC, Ferreira R, Valle LA, Manfredi GG do P, Stuani V de T, Pavani AP de S, et al. Splinted or nonsplinted short dental implants in posterior mandible: a systematic review and meta-analysis. *Res Soc Dev.* 2022;11(14):e189111436282. doi: http://dx.doi.org/10.33448/rsd-v11i14.36282

10. Barausse C, Tayeb S, Pellegrino G, Bonifazi L, Mancuso E, Ratti S, et al. The Inlay Technique in Alveolar Ridge Augmentation: A Systematic Review. *J Clin Med*. 2025;14(5):1684. doi:10.3390/jcm14051684

11. Mugri MH, Sayed ME, Bhandi S, Alaqi H a. A, Alsubeaie NHB, Alsubaie SH, et al. Success Rate of Immediately Loaded Implants in the Posterior Zone. Niger *J Clin Pract*. 2023; 26(9):1215-25. doi: 10.4103/njcp.njcp_884_22

12. Kadkhodazadeh M, Alimardani Y, Azadi A, Daneshvar A, Amid R, Khaleghi A. Clinical outcomes of implants placed with transcrestal maxillary sinus elevation: a systematic review and meta-analysis. *Br J Oral Maxillofac Surg.* 2024;62(8):685-703. doi: 10.1016/j.bjoms.2024.05.006

13. Shi S, Han L, Su J, Guo J, Yu F, Zhang W. Clinical efficacy of transcrestal sinus floor augmentation, in comparison with lateral approach, in sites with residual bone height ≤6 mm: A systematic review and meta-analysis. *Clin Oral Implants Res*. 2023; 34(11):1151-75. doi:10.1111/clr.14155

14. Yang J, Cheng Z, Shi B. Augmentation of the alveolar ridge compared with shorter implants in atrophic jaws: a meta-analysis based on randomised controlled trials. Br J Oral Maxillofac Surg. enero de 2016;54(1):68-73. doi: 10.1016/j.bjoms.2015.10.011
