# Supplementary material for: Clinical performance of short implants vs. standard implants in edentulous patients. An umbrella review
Source: Front Oral Health. 2025 Sep 18;6:1670095. doi: 10.3389/froh.2025.1670095 (PMC12488567; doi:10.3389/froh.2025.1670095)
Supplement: Supplementary Material S3 — Characteristics of included studies—a table summarizing the characteristics of the systematic reviews included in the analysis. [file Table3.docx]

Supplementary Material 4. Assessment of the methodological quality and the quality of the evidence of the included studies

| Authors | Year | AMSTAR – 2 | | | | | | | | | | | | | | | | Overall confidence |
| --- | --- | --- | --- | --- | --- | --- | --- | --- | --- | --- | --- | --- | --- | --- | --- | --- | --- | --- |
|  |  | 1 | 2* | 3 | 4* | 5 | 6 | 7* | 8 | 9* | 10 | 11* | 12 | 13* | 14 | 15* | 16 |  |
| Alemán et al. (1) | 2025 | Yes | Yes partial | Yes | Yes partial | Yes | Yes | Yes partial | Yes | Yes | Yes | Yes | Yes | Yes | Yes | No | Yes | Low |
| Abayov et al. (2) | 2024 | Yes | Yes partial | Yes | Yes partial | Yes | No | Yes partial | Yes | Yes partial | Yes | Yes | Yes | Yes | Yes | No | No | Low |
| Zhang et al. (3) | 2024 | Yes | Yes | Yes | Yes partial | Yes | Yes | Yes partial | Yes | Yes | Yes | Yes | Yes | Yes | Yes | Yes | Yes | High |
| Emfietzoglou et al. (4) | 2024 | Yes | Yes | Yes | Yes partial | Yes | Yes | Yes | Yes | Yes | Yes | Yes | Yes | Yes | Yes | No | Yes | Low |
| Liang et al. (5) | 2024 | Yes | Yes | Yes | Yes partial | Yes | Yes | Yes partial | Yes | Yes | Yes | Yes | Yes | Yes | Yes | Yes | No | High |
| Kermanshah et al. (6) | 2023 | Yes | Yes | Yes | Yes | Yes | Yes | Yes | Yes | Yes | Yes | Yes | Yes | Yes | Yes | Yes | Yes | High |
| Mester et al. (7) | 2023 | Yes | Yes | Yes | Yes | Yes | No | Yes | Yes | Yes | Yes | Yes | No | Yes | Yes | No | Yes | Low |
| Rosa et al. (8) | 2023 | Yes | Yes partial | Yes | Yes partial | Yes | Yes | Yes | Yes | Yes | Yes | No meta-analysis | | Yes | No | No meta-analysis | Yes | High |
| Grunau et al. (9) | 2023 | Yes | Yes | Yes | No | No | No | Yes | Yes | Yes | Yes | Yes | No | Yes | Yes | No | Yes | Critically low |
| Zhang et al. (10) | 2023 | Yes | Yes partial | Yes | Yes partial | Yes | Yes | Yes partial | Yes | Yes partial | Yes | Yes | Yes | Yes | Yes | Yes | Yes | High |
| Liang et al. (11) | 2022 | Yes | Yes | Yes | Yes partial | Yes | Yes | Yes partial | Yes | Yes | Yes | Yes | Yes | Yes | Yes | No | No | Low |
| Wang et al. (12) | 2022 | Yes | Yes partial | Yes | Yes partial | Yes | Yes | Yes partial | Yes | Yes | Yes | Yes | Yes | Yes | Yes | No | Yes | Low |
| Medikeri et al. (13) | 2022 | Yes | Yes | Yes | Yes partial | Yes | Yes | Yes partial | Yes | Yes | Yes | Yes | Yes | Yes | Yes | Yes | Yes | High |
| Toledano et al. (14) | 2022 | Yes | Yes | Yes | Yes partial | Yes | Yes | Yes | Yes | Yes | Yes | Yes | Yes | Yes | Yes | Yes | Yes | High |
| Guida et al. (15) | 2022 | Yes | Yes | Yes | Yes partial | Yes | Yes | Yes | Yes | Yes | Yes | Yes | Yes | Yes | Yes | Yes | Yes | High |
| Tang et al. (16) | 2022 | Yes | Yes | Yes | Yes partial | Yes | Yes | Yes | Yes | Yes | Yes | Yes | Yes | Yes | Yes | Yes | Yes | High |
| Terheyden et al. (17) | 2021 | Yes | Yes partial | Yes | No | No | No | Yes partial | Yes | Yes | Yes | Yes | Yes | Yes | Yes | No | Yes | Critically low |
| Moraschini et al. (18) | 2021 | Yes | Yes | Yes | Yes | Yes | Yes | Yes | Yes | Yes | Yes | Yes | Yes | Yes | Yes | Yes | Yes | High |
| Bitinas et al. (19) | 2021 | Yes | Yes partial | Yes | No | No | No | Yes partial | Yes | Yes partial | Yes | Yes | Yes | Yes | Yes | No | Yes | Critically low |
| Yu et al. (20) | 2021 | Yes | Yes | Yes | Yes partial | Yes | Yes | Yes | Yes | Yes | Yes | Yes | Yes | Yes | Yes | Yes | Yes | High |
| Wu et al. (21) | 2021 | Yes | Yes | Yes | Yes partial | Yes | Yes | Yes | Yes | Yes | Yes | Yes | Yes | Yes | Yes | Yes | Yes | High |
| Chaware et al. (22) | 2021 | Yes | Yes | Yes | Yes partial | Yes | Yes | Yes partial | Yes | Yes | Yes | Yes | Yes | Yes | Yes | Yes | Yes | High |
| Abdel-Halim et al. (23) | 2021 | Yes | Yes | Yes | Yes partial | Yes | No | Yes partial | Yes | No | Yes | Yes | Yes | Yes | Yes | Yes | Yes | Low |
| Carosi et al. (24) | 2021 | Yes | Yes partial | Yes | Yes partial | Yes | Yes | Yes | Yes | Yes | Yes | Yes | Yes | Yes | Yes | Yes | Yes | High |
| Carosi et al. (25) | 2021 | Yes | Yes partial | Yes | Yes partial | Yes | Yes | Yes | Yes | Yes | Yes | No meta-analysis | | Yes | Yes | No meta-analysis | Yes | High |
| Xu et al. (26) | 2020 | Yes | Yes | Yes | Yes partial | Yes | Yes | Yes partial | Yes | Yes | Yes | Yes | Yes | Yes | Yes | No | Yes | Low |
| Lozano-Carrascal et al. (27) | 2020 | Yes | Yes | Yes | Yes | Yes | Yes | Yes | Yes | Yes | Yes | Yes | Yes | Yes | Yes | No | Yes | Low |
| Xu et al. (28) | 2020 | Yes | Yes | Yes | Yes partial | Yes | Yes | Yes partial | Yes | Yes | Yes | Yes | Yes | Yes | Yes | No | No | Low |
| Iezzi et al. (29) | 2020 | Yes | Yes | Yes | Yes partial | Yes | Yes | Yes | Yes | Yes | Yes | Yes | Yes | Yes | Yes | No | Yes | Low |
| Vazouras et al. (30) | 2020 | Yes | Yes | Yes | No | Yes | Yes | Yes | Yes | Yes | Yes | Yes | Yes | Yes | Yes | No | Yes | Critically low |
| Mokcheh et al. (31) | 2019 | Yes | Yes partial | Yes | Yes partial | Yes | Yes | Yes partial | Yes | Yes | Yes | Yes | Yes | Yes | Yes | No | Yes | Low |
| Nielsen et al. (32) | 2019 | Yes | Yes | Yes | Yes partial | Yes | No | Yes partial | Yes | Yes partial | Yes | Yes | Yes | Yes | Yes | No | Yes | Low |
| Altaib et al. (33) | 2019 | Yes | Yes | Yes | Yes partial | Yes | Yes | Yes | Yes | Yes | Yes | Yes | Yes | Yes | Yes | No | Yes | Low |
| Ravidà et al. (34) | 2019 | Yes | Yes | Yes | Yes | Yes | Yes | Yes partial | Yes | Yes | Yes | Yes | Yes | Yes | Yes | Yes | Yes | High |
| Esposito et al. (35) | 2019 | Yes | Yes partial | Yes | Yes partial | Yes | Yes | Yes partial | Yes | Yes | Yes | Yes | Yes | Yes | Yes | No | Yes | Low |
| Amine et al. (36) | 2019 | Yes | Yes partial | Yes | Yes partial | Yes | Yes | Yes partial | Yes | Yes | Yes | No meta-analysis | | Yes | Yes | No meta-analysis | Yes | High |
| Chen et al. (37) | 2019 | Yes | Yes | Yes | Yes partial | No | No | Yes partial | Yes | Yes | Yes | Yes | Yes | Yes | Yes | Yes | Yes | Moderate |
| Bitaraf et al. (38) | 2019 | Yes | Yes | Yes | Yes | Yes | Yes | Yes | Yes | Yes | Yes | Yes | Yes | Yes | Yes | Yes | No | High |
| Ravidà et al. (39) | 2019 | Yes | Yes | Yes | Yes | Yes | Yes | Yes partial | Yes | Yes | Yes | Yes | Yes | Yes | Yes | Yes | Yes | High |
| Yan et al. (40) | 2019 | Yes | Yes | Yes | Yes partial | Yes | Yes | Yes partial | Yes | Yes | Yes | Yes | Yes | Yes | Yes | Yes | Yes | High |
| Aldawood et al. (41) | 2019 | Yes | Yes partial | Yes | No | Yes | Yes | Yes partial | No | No | Yes | Yes | No | No | No | No | Yes | Critically low |
| de N Dias et al. (42) | 2019 | Yes | Yes partial | Yes | Yes partial | Yes | Yes | Yes | Yes | Yes | Yes | Yes | No | No | No | No | Yes | Critically low |
| Uehara et al. (43) | 2018 | Yes | Yes | Yes | Yes partial | No | Yes | Yes partial | Yes | Yes | Yes | Yes | Yes | Yes | Yes | Yes | Yes | High |
| Palacios et al. (44) | 2018 | Yes | Yes | Yes | Yes partial | No | Yes | Yes partial | Yes | Yes | Yes | Yes | Yes | Yes | Yes | No | Yes | Low |
| Papaspyridakos et al. (45) | 2018 | Yes | Yes | Yes | Yes partial | Yes | Yes | Yes partial | Yes | Yes | Yes | Yes | Yes | Yes | Yes | No | Yes | Low |
| Starch-Jensen et al. (46) | 2018 | Yes | Yes | Yes | Yes partial | Yes | No | Yes partial | Yes | Yes | Yes | No meta-analysis | | Yes | Yes | No meta-analysis | Yes | High |
| Cruz et al. (47) | 2018 | Yes | Yes | Yes | Yes partial | Yes | No | Yes | Yes | Yes | Yes | Yes | Yes | Yes | Yes | Yes | Yes | High |
| de Souza et al. (48) | 2018 | Yes | Yes partial | Yes | Yes partial | Yes | Yes | Yes | Yes | Yes | Yes | Yes | Yes | Yes | Yes | No | Yes | Low |
| Fan et al. (49) | 2017 | Yes | Yes partial | Yes | Yes partial | No | Yes | Yes partial | Yes | Yes | Yes | Yes | Yes | Yes | Yes | No | Yes | Low |
| Tong et al. (50) | 2017 | Yes | No | Yes | Yes partial | No | Yes | Yes partial | Yes | Yes | Yes | Yes | Yes | Yes | Yes | No | Yes | Critically low |
| Toti et al. (51) | 2017 | Yes | Yes partial | Yes | Yes partial | Yes | Yes | Yes partial | Yes | Yes | Yes | Yes | Yes | Yes | Yes | No | Yes | Low |
| Lemos et al. (52) | 2016 | Yes | Yes | Yes | Yes | Yes | Yes | Yes | Yes | Yes | Yes | Yes | Yes | Yes | Yes | No | No | Low |
| Camps-Font et al. (53) | 2016 | Yes | No | Yes | Yes partial | Yes | Yes | Yes partial | Yes | Yes | Yes | Yes | Yes | Yes | Yes | No | Yes | Critically low |
| Thoma et al. (54) | 2015 | Yes | Yes partial | Yes | No | Yes | Yes | Yes | Yes | Yes | Yes | No meta-analysis | | Yes | No | No meta-analysis | Yes | Low |
| Nisand et al. (55) | 2015 | Yes | Yes partial | Yes | No | Yes | Yes | Yes partial | Yes | Yes | Yes | No meta-analysis | | Yes | Yes | No meta-analysis | No | Low |
| Lee et al. (56) | 2014 | Yes | Yes partial | Yes | Yes partial | No | Yes | Yes partial | Yes | Yes | Yes | Yes | Yes | Yes | Yes | No | Yes | Low |
| Monje et al. (57) | 2014 | Yes | Yes partial | Yes | Yes partial | No | No | Yes partial | Yes | Yes partial | Yes | Yes | Yes | Yes | Yes | No | Yes | Low |
| Mezzomo et al. (58) | 2014 | Yes | Yes partial | Yes | Yes | Yes | Yes | Yes | Yes | Yes | Yes | Yes | No | Yes | Yes | No | Yes | Low |
| Monje et al. (59) | 2013 | Yes | No | Yes | Yes partial | No | No | Yes partial | Yes | No | Yes | Yes | No | No | Yes | No | Yes | Critically low |
| Kotsovilis et al. (60) | 2009 | Yes | No | Yes | Yes partial | Yes | Yes | Yes | Yes | Yes partial | Yes | Yes | Yes | Yes | Yes | Yes | No | Low |

AMSTAR = A MeaSurement Tool to Assess Systemic Reviews

1 = Did the research questions and inclusion criteria for the review include the components of PICO?

2 = Did the report of the review contain an explicit statement that the review methods were established prior to the conduct of the review and did the report justify any significant deviations from the protocol?

3 = Did the review authors explain their selection of the study designs for inclusion in the review?

4 = Did the review authors use a comprehensive literature search strategy?

5 = Did the review authors perform study selection in duplicate?

6 = Did the review authors perform data extraction in duplicate?

7 = Did the review authors provide a list of excluded studies and justify the exclusions?

8 = Did the review authors describe the included studies in adequate detail?

9 = Did the review authors use a satisfactory technique for assessing the risk of bias (RoB) in individual studies that were included in the review?

10 = Did the review authors report on the sources of funding for the studies included in the review?

11 = If meta-analysis was performed, did the review authors use appropriate methods for statistical combination of results?

12 = If meta-analysis was performed, did the review authors assess the potential impact of RoB in individual studies on the results of the meta-analysis or other evidence synthesis?

13 = Did the review authors account for RoB in primary studies when interpreting/discussing the results of the review?

14 = Did the review authors provide a satisfactory explanation for, and discussion of, any heterogeneity observed in the results of the review?

15 = If they performed quantitative synthesis did the review authors carry out an adequate investigation of publication bias (small study bias) and discuss its likely impact on the results of the review?

16 = Did the review authors report any potential sources of conflict of interest, including any funding they received for conducting the review?

* = Critical domain

**References**

1. Alemán BO, Rivera-Velazquez I, Jana-Hernández Z, Rivas-Tumanyan S, Guerrero-Rodríguez LM, Elias-Boneta AR. Long-Term Outcomes of Short versus Long Dental Implants with Sinus Lift in Atrophied Posterior Maxillae: A Systematic Review and Meta-Analysis. *P R Health Sci J*. 2025;44(1):54-62.

2. Abayov P, Sarikov R, Nazarenko LM, Babich O, Haimov E, Juodzbalys G. Outcome Difference between Short and Longer Dental Implants Placed Simultaneously with Alveolar Bone Augmentation: a Systematic Review and Meta-Analysis. *J Oral Maxillofac Res.* 2024 ;15(2):e2. doi:10.5037/jomr.2024.15202

3. Zhang Y, Tang X, Zhang Y, Cao C. A network meta-analysis comparing treatment modalities of short and long implants in the posterior maxilla with insufficient bone height. *BMC Oral Health*. 2024;24(1). doi:10.1186/s12903-024-05377-1

4. Emfietzoglou R, Dereka X. Survival Rates of Short Dental Implants (≤6 mm) Used as an Alternative to Longer (>6 mm) Implants for the Rehabilitation of Posterior Partial Edentulism: A Systematic Review of RCTs. *Dent J*. 2024;12(6). doi:10.3390/dj12060185

5. Liang L, Wu X, Yan Q, Shi B. Are short implants (≤8.5 mm) reliable in the rehabilitation of completely edentulous patients: A systematic review and meta-analysis. *J Prosthet Dent*. 2024;131(5):826-32. doi: 0.1186/s12903-024-05377-1

6. Kermanshah H, Keshtkar A, Hassani A, Bitaraf T. Comparing short implants to standard dental implants: a systematic review and meta-analysis of randomized controlled trials with extended follow-up. *Evid Based Den*. 2023;24(4):192-3. doi:10.1038/s41432-023-00924-1

7. Mester A, Onisor F, Stasio DD, Piciu A, Cosma AM, Bran S. Short Implants versus Standard Implants and Sinus Floor Elevation in Atrophic Posterior Maxilla: A Systematic Review and Meta-Analysis of Randomized Clinical Trials with ≥5 Years’ Follow-Up. *J Pers Med*. 2023;13(2):169. doi:10.3390/jpm13020169.

8. Rosa A, nueva E a sitio externo E enlace se abrirá en una ventana, Pujia AM, Arcuri C. Complete Full Arch Supported by Short Implant (<8 mm) in Edentulous Jaw: A Systematic Review. *Appl Sci.* 2023;13(12):7162. doi: 10.3390/app13127162.

9. Grunau O, Terheyden H. Lateral augmentation of the sinus floor followed by regular implants versus short implants in the vertically deficient posterior maxilla: a systematic review and timewise meta-analysis of randomized studies. *Int J Oral Maxillofac Surg*. 2023;52(7):813-24. doi: 10.1016/j.ijom.2022.11.015

10. 启航张, 佳明龚, 佳颖余, 瑞敏赵, 萍苟, 占海余. 在后牙区应用4 mm超短种植体临床效果的Meta分析. *West China J Stomatol.* 2023;41(1):80. doi: 10.7518/hxkq.2023.01.011

11. Liang L, Wu X, Yan Q, Shi B. Are short implants (≤8.5 mm) reliable in the rehabilitation of completely edentulous patients: A systematic review and meta-analysis. *J Prosthet Dent*. 2022;S0022-3913(22):140-8. doi: 10.1016/j.prosdent.2022.02.015

12. Wang M, Liu F, Ulm C, Shen H, Rausch-Fan X. Short Implants versus Longer Implants with Sinus Floor Elevation: A Systemic Review and Meta-Analysis of Randomized Controlled Trials with a Post-Loading Follow-Up Duration of 5 Years. *Materials.* 2022;15(13):4722. doi:10.3390/ma15134722

13. Medikeri RS, Pereira MA, Waingade M, Navale S. Survival of surface-modified short versus long implants in complete or partially edentulous patients with a follow-up of 1 year or more: a systematic review and meta-analysis. *J Periodontal Implant Sci*. 2022;52(2):261-81. doi:10.5051/jpis.2007340367

14. Toledano M, Fernández-Romero E, Vallecillo C, Toledano R, Osorio MT, Vallecillo-Rivas M. Short versus standard implants at sinus augmented sites: a systematic review and meta-analysis. *Clin Oral Investig*. 2022;26(11):6681-98. doi:10.1007/s00784-022-04628-1

15. Guida L, Bressan E, Cecoro G, Volpe AD, Fabbro MD, Annunziata M. Short versus Longer Implants in Sites without the Need for Bone Augmentation: A Systematic Review and Meta-Analysis of Randomized Controlled Trials. *Materials.* 2022;15(9). doi:10.3390/ma15093138

16. Tang C, Du Q, Luo J, Peng L. Simultaneous placement of short implants (≤ 8 mm) versus standard length implants (≥ 10 mm) after sinus floor elevation in atrophic posterior maxillae: a systematic review and meta-analysis. *Int J Implant Dent.* 2022;8(1):45. doi:10.1186/s40729-022-00443-1

17. Terheyden H, Meijer GJ, Raghoebar GM. Vertical bone augmentation and regular implants versus short implants in the vertically deficient posterior mandible: a systematic review and meta-analysis of randomized studies. *Int J Oral Maxillofac Surg.* 2021;50(9):1249-58. doi: 10.1016/j.ijom.2021.01.005

18. Moraschini V, Mourão CF de AB, Montemezzi P, Kischinhevsky ICC, de Almeida DCF, Javid K, et al. Clinical Comparation of Extra-Short (4 mm) and Long (>8 mm) Dental Implants Placed in Mandibular Bone: A Systematic Review and Metanalysis. *Healthc Basel Switz*. 2021;9(3):315. doi:10.3390/healthcare9030315

19. Bitinas D, Bardijevskyt G. Short implants without bone augmentation vs. long implants with bone augmentation: systematic review and meta-analysis. *Aust Dent J.* 2021;66(S1):S71-81. doi:10.1111/adj.12859

20. Yu X, Ruogu X, Zhengchuan Z, Yang Y, Feilong D. A meta-analysis indicating extra-short implants (≤ 6 mm) as an alternative to longer implants (≥ 8 mm) with bone augmentation. *Sci Rep Nat Publ Group.* 2021;11(1):8152. doi:10.1038/s41598-021-87507-1

21. Wu H, Shi Q, Huang Y, Chang P, Huo N, Jiang Y, et al. Failure Risk of Short Dental Implants Under Immediate Loading: A Meta-Analysis. *J Prosthodont*. 2021;30(7):569-80. doi:10.1111/jopr.13376

22. Chaware S, Thakare V, Chaudhary R, Jankar A, Thakkar S, Borse S. The rehabilitation of posterior atrophic maxilla by using the graftless option of short implant versus conventional long implant with sinus graft: A systematic review and meta-analysis of randomized controlled clinical trial. *J Indian Prosthodont Soc.* 2021;21(1):28-44. doi: 10.4103/jips.jips_400_20

23. Abdel-Halim M, Issa D, Chrcanovic BR, nueva E a sitio externo E enlace se abrirá en una ventana. The Impact of Dental Implant Length on Failure Rates: A Systematic Review and Meta-Analysis. *Materials.* 2021;14(14):3972. doi:10.3390/ma14143972

24. Carosi P, Lorenzi C, Lio F, Laureti M, Ferrigno N, Arcuri C. Short implants (≤6mm) as an alternative treatment option to maxillary sinus lift. *Int J Oral Maxillofac Surg.* 2021;50(11):1502-10. doi: 10.1016/j.ijom.2021.02.014

25. Carosi P, Lorenzi C, Laureti M, Ferrigno N, Arcuri C. Short Dental Implants (≤ 6 mm) to Rehabilitate Severe Mandibular Atrophy: A Systematic Review. *Int J Oral Maxillofac Implants.* 2021;36(1):30-7. doi:10.11607/jomi.8510

26. Xu X, Huang J, Fu X, Kuang Y, Yue H, Song J, et al. Short implants versus longer implants in the posterior alveolar region after an observation period of at least five years: A systematic review and meta-analysis. *J Dent*. 2020;100:103386. doi: 10.1016/j.jdent.2020.103386

27. Lozano-Carrascal N, Anglada-Bosqued A, Salomó-Coll O, Hernández-Alfaro F, Wang HL, Gargallo-Albiol J. Short implants (<8mm) versus longer implants (≥8mm) with lateral sinus floor augmentation in posterior atrophic maxilla: A meta-analysis of RCT`s in humans. *Med Oral Patol Oral Cirugia Bucal.* 2020;25(2):e168-79. doi: 10.4317/medoral.23248

28. Xu X, Hu B, Xu Y, Liu Q, Ding H, Xu L. Short versus standard implants for single-crown restorations in the posterior region: A systematic review and meta-analysis. J Prosthet Dent. 2020;124(5):530-8. doi: 10.1016/j.prosdent.2019.09.030

29. Iezzi G, Perrotti V, Felice P, Barausse C, Piattelli A, Del Fabbro M. Are <7-mm long implants in native bone as effective as longer implants in augmented bone for the rehabilitation of posterior atrophic jaws? A systematic review and meta-analysis. *Clin Implant Dent Relat Res*. 2020;22(5):552-66. doi:10.1111/cid.12946

30. Vazouras K, de Souza AB, Gholami H, Papaspyridakos P, Pagni S, Weber HP. Effect of time in function on the predictability of short dental implants (≤6 mm): A meta-analysis. *J Oral Rehabil.* 2020;47(3):403-15. doi:10.1111/joor.12925

31. Mokcheh A, Jegham H, Turki S. Short implants as an alternative to sinus lift for the rehabilitation of posterior maxillary atrophies: Systematic review and meta-analysis. *J Stomatol Oral Maxillofac Surg*. 2019;120(1):28-37. doi:10.1016/j.jormas.2018.11.006

32. Nielsen HB, Schou S, Isidor F, Christensen AE, Starch-Jensen T. Short implants (≤8mm) compared to standard length implants (>8mm) in conjunction with maxillary sinus floor augmentation: a systematic review and meta-analysis. *Int J Oral Maxillofac Surg*. 2019;48(2):239-49. doi:10.1016/j.ijom.2018.05.010

33. Altaib FH, Alqutaibi AY, Al-Fahd A, Eid S. Short dental implant as alternative to long implant with bone augmentation of the atrophic posterior ridge: A systematic review and meta-analysis of RCTs. *Quintessence Int.* 2019;50(8):636-51. doi:10.3290/j.qi.a42948

34. Ravidà A, Wang IC, Sammartino G, Barootchi S, Tattan M, Troiano G, et al. Prosthetic Rehabilitation of the Posterior Atrophic Maxilla, Short (≤6 mm) or Long (≥10 mm) Dental Implants? A Systematic Review, Meta-analysis, and Trial Sequential Analysis: Naples Consensus Report Working Group A. Implant Dent. 2019;28(6):590-602. doi:10.1097/ID.0000000000000919

35. Esposito M, Buti J, Barausse C, Gasparro R, Sammartino G, Felice P. Short implants versus longer implants in vertically augmented atrophic mandibles: A systematic review of randomised controlled trials with a 5-year post-loading follow-up. Int J Oral Implantol. 2019;12(3):267-80.

36. Amine M, Guelzim Y, Benfaida S, Bennani A, Andoh A. Short implants (5–8 mm) vs. long implants in augmented bone and their impact on peri-implant bone in maxilla and/or mandible: Systematic review. J Stomatol Oral Maxillofac Surg. 2019;120(2):133-42. doi:10.1016/j.jormas.2018.11.007

37. Chen S, Ou Q, Wang Y, Lin X. Short implants (5‐8 mm) vs long implants (≥10 mm) with augmentation in atrophic posterior jaws: A meta‐analysis of randomised controlled trials. *J Oral Rehabil.* 2019;46(12):1192-203. doi:10.1111/joor.12860

38. Bitaraf T, Keshtkar A, Rokn AR, Monzavi A, Geramy A, Hashemi K. Comparing short dental implant and standard dental implant in terms of marginal bone level changes: A systematic review and meta-analysis of randomized controlled trials. *Clin Implant Dent Relat Res*. 2019;21(4):796-812. doi:10.1111/cid.12774

39. Ravidà A, Wang IC, Barootchi S, Askar H, Tavelli L, Gargallo-Albiol J, et al. Meta-analysis of randomized clinical trials comparing clinical and patient-reported outcomes between extra-short (≤6 mm) and longer (≥10 mm) implants. *J Clin Periodontol.* 2019;46(1):118-42. doi:10.1111/jcpe.13026

40. Yan Q, Wu X, Su M, Hua F, Shi B. Short implants (≤6 mm) versus longer implants with sinus floor elevation in atrophic posterior maxilla: A systematic review and meta-analysis. *BMJ Open*. 2019;9(10). doi:10.1136/bmjopen-2019-029826

41. Aldawood T, Qarni M, Alhayek A, Muslih W, Alfantoukh A, Albeladi R, et al. Comparison between short dental implants versus standard dental implants of posterior jaws: A systematic review & meta-analysis. World J Pharm Res. 2019;8:1501-13. doi: 10.20959/wjpr201912-16180

42. de N Dias FJ, Pecorari VGA, Martins CB, Del Fabbro M, Casati MZ. Short implants versus bone augmentation in combination with standard-length implants in posterior atrophic partially edentulous mandibles: systematic review and meta-analysis with the Bayesian approach. *Int J Oral Maxillofac Surg.* 2019;48(1):90-6. doi: 10.1016/j.ijom.2018.05.009

43. Uehara PN, Matsubara VH, Igai F, Sesma N, Mukai MK, Araujo MG. Short dental implants (≤7mm) versus longer implants in augmented bone area: A meta-analysis of randomized controlled trials. *Open Dent J.* 2018;12(1):354-65. doi: 10.2174/1874210601812010354

44. Palacios JAV, Garcia JJ, Caramês JMM, Quirynen M, da Silva Marques DN. Short implants versus bone grafting and standard-length implants placement: a systematic review. *Clin Oral Investig.* 2018;22(1):69-80. doi:10.1007/s00784-017-2205-0

45. Papaspyridakos P, De Souza A, Vazouras K, Gholami H, Pagni S, Weber HP. Survival rates of short dental implants (≤6 mm) compared with implants longer than 6 mm in posterior jaw areas: A meta‐analysis. *Clin Oral Implants Res*. 2018;29(S16):8-20. doi:10.1111/clr.13289

46. Starch-Jensen T, Nielsen HB. Prosthetic Rehabilitation of the Partially Edentulous Atrophic Posterior Mandible with Short Implants (≤ 8 mm) Compared with the Sandwich Osteotomy and Delayed Placement of Standard Length Implants (> 8 mm): a Systematic Review. *J Oral Maxillofac Res.* 2018;9(2):e2. doi:10.5037/jomr.2018.9202

47. Cruz RS, de Araújo Lemos CA, de Souza Batista VE, e Oliveira HFF, de Luna Gomes JM, Pellizzer EP, et al. Short implants versus longer implants with maxillary sinus lift. A systematic review and meta-analysis. *Braz Oral Res.* 2018;32:e86. doi:10.1590/1807-3107bor-2018.vol32.0086

48. Tolentino da Rosa de Souza P, Binhame Albini Martini M, Reis Azevedo-Alanis L. Do short implants have similar survival rates compared to standard implants in posterior single crown?: A systematic review and meta-analysis. *Clin Implant Dent Relat Res*. 2018;20(5):890-901. doi:10.1111/cid.12634

49. Fan T, Li Y, Deng WW, Wu T, Zhang W. Short Implants (5 to 8 mm) Versus Longer Implants (>8 mm) with Sinus Lifting in Atrophic Posterior Maxilla: A Meta-Analysis of RCTs. *Clin Implant Dent Relat Res.* 2017;19(1):207-15. doi:10.1111/cid.12432

50. Tong Q, Zhang X, Yu L. Meta-analysis of randomized controlled trials comparing clinical outcomes between short implants and long implants with bone augmentation procedure. *Int J Oral Maxillofac Implants.* 2017;32(1):e25-34. doi:10.11607/jomi.4793

51. Toti P, Marchionni S, Menchini-Fabris GB, Marconcini S, Covani U, Barone A. Surgical techniques used in the rehabilitation of partially edentulous patients with atrophic posterior mandibles: A systematic review and meta-analysis of randomized controlled clinical trials. *J Cranio-Maxillo-fac Surg Off Publ Eur Assoc Cranio-Maxillo-fac Surg.* 2017;45(8):1236-45. doi:10.1016/j.jcms.2017.04.011

52. Lemos CAA, Ferro-Alves ML, Okamoto R, Mendonça MR, Pellizzer EP. Short dental implants versus standard dental implants placed in the posterior jaws: A systematic review and meta-analysis. *J Dent.* 2016;47:8-17. doi:10.1016/j.jdent.2016.01.005

53. Camps-Font O, Burgueño-Barris G, Figueiredo R, Jung RE, Gay-Escoda C, Valmaseda-Castellón E. Interventions for Dental Implant Placement in Atrophic Edentulous Mandibles: Vertical Bone Augmentation and Alternative Treatments. A Meta-Analysis of Randomized Clinical Trials. *J Periodontol.* 2016;87(12):1444-57. doi:10.1902/jop.2016.160226

54. Thoma DS, Zeltner M, Hüsler J, Hämmerle CHF, Jung RE. EAO Supplement Working Group 4 - EAO CC 2015 Short implants versus sinus lifting with longer implants to restore the posterior maxilla: A systematic review. *Clin Oral Implants Res*. 2015; 26:154-69. doi:10.1111/clr.12615

55. Nisand D, Picard N, Rocchietta I. Short implants compared to implants in vertically augmented bone: a systematic review. *Clin Oral Implants Res.* 2015;26(S11):170-9. doi:10.1111/clr.12632

56. Lee SA, Lee CT, Fu MM, Elmisalati W, Chuang SK. Systematic review and meta-analysis of randomized controlled trials for the management of limited vertical height in the posterior region: Short implants (5 to 8 mm) vs longer implants (> 8 mm) in vertically augmented sites. *Int J Oral Maxillofac Implants.* 2014;29(5):1085-97. doi:10.11607/jomi.3504

57. Monje A, Suarez F, Galindo-Moreno P, García-Nogales A, Fu JH, Wang HL. A systematic review on marginal bone loss around short dental implants (<10 mm) for implant-supported fixed prostheses. *Clin Oral Implants Res*. 2014;25(10):1119-24. doi:10.1111/clr.12236

58. Mezzomo LA, Miller R, Triches D, Alonso F, Shinkai RSA. Meta-analysis of single crowns supported by short (<10 mm) implants in the posterior region. *J Clin Periodontol*. 2014;41(2):191-213. doi:10.1111/jcpe.12180

59. Monje A, Chan HL, Fu JH, Suarez F, Galindo-Moreno P, Wang HL. Are short dental implants (<10 mm) effective? A meta-analysis on prospective clinical trials. *J Periodontol*. 2013;84(7):895-904. doi:10.1902/jop.2012.120328

60. Kotsovilis S, Fourmousis I, Karoussis IK, Bamia C. A systematic review and meta-analysis on the effect of implant length on the survival of rough-surface dental implants. *J Periodontol.* 2009;80(11):1700-18. doi:10.1902/jop.2009.090107
