# Supplementary material for: Clinical performance of short implants vs. standard implants in edentulous patients. An umbrella review
Source: Front Oral Health. 2025 Sep 18;6:1670095. doi: 10.3389/froh.2025.1670095 (PMC12488567; doi:10.3389/froh.2025.1670095)
Supplement: Supplementary Material S4 — Assessment of the methodological quality and the quality of the evidence of the included studies—a summary of the quality assessments for the included systematic reviews using the AMSTAR-2 tool. [file Table4.docx]

Supplementary Material 5. Overlapping of primary studies in systematic reviews

| **Primary studies** | **Systematic reviews that included the primary studies** | **Times that primary studies were included** |
| --- | --- | --- |
| Pistilli et al. (1) | Grunau et al. (2), Terheyden et al. (3), Bitinas et al. (4), Chaware et al. (5), Abdel-Halim et al. (6), Lozano-Carrascal et al. (7), Iezzi et al. (8), Vazouras et al. (9), Mokcheh et al. (10), Altaib et al. (11), Ravidà et al. (12), Amine et al. (13), Chen et al. (14), Bitaraf et al. (15), Ravidà et al. (16), de N Dias et al. (17), Uehara et al. (18), Palacios et al. (19), Papaspyridakos et al. (20), Starch-Jensen et al. (21), Cruz et al. (22), Fan et al. (23), Tong et al. (24), Toti et al. (25), Lemos et al. (26), Camps-Font et al. (27), Thoma et al. (28), Nisand et al. (29) | 28 |
| Esposito et al. (30) | Grunau et al. (2), Medikeri et al. (31), Terheyden et al. (3), Bitinas et al. (4), Yu et al. (32), Chaware et al. (5), Lozano-Carrascal et al. (7), Iezzi et al. (8), Vazouras et al. (9), Mokcheh et al. (10), Nielsen et al. (33), Altaib et al. (11), Ravidà et al. (12), Amine et al. (13), Chen et al. (14), Bitaraf et al. (15), Ravidà et al. (16), Aldawood et al. (34), Palacios et al. (19), Papaspyridakos et al. (20), Starch-Jensen et al. (21), Cruz et al. (22), Fan et al. (23), Tong et al. (24), Lemos et al. (26), Camps-Font et al. (27), Nisand et al. (29) | 27 |
| Pistilli et al. (35) | Grunau et al. (2), Medikeri et al. (31), Terheyden et al. (3), Yu et al. (32), Chaware et al. (5), Abdel-Halim et al. (6), Iezzi et al. (8), Mokcheh et al. (10), Altaib et al. (11), Ravidà et al. (12), Amine et al. (13), Chen et al. (14), Bitaraf et al. (15), Ravidà et al. (16), de N Dias et al. (17), Uehara et al. (18), Palacios et al. (19), Papaspyridakos et al. (20), Cruz et al. (22), Fan et al. (23), Tong et al. (24), Toti et al. (25), Lemos et al. (26), Camps-Font et al. (27), Thoma et al. (28), Nisand et al. (29) | 26 |
| Bechara et al. (36) | Abayov et al. (37), Zhang et al. (38), Kermanshah et al. (39), Grunau et al. (2), Medikeri et al. (31), Toledano et al. (40), Bitinas et al. (4), Yu et al. (32), Chaware et al. (5), Abdel-Halim et al. (6), Carosi et al. (41), Lozano-Carrascal et al. (7), Iezzi et al. (8), Vazouras et al. (9), Mokcheh et al. (10), Nielsen et al. (33), Altaib et al. (11), Ravidà et al. (12), Chen et al. (14), Bitaraf et al. (15), Ravidà et al. (16), Yan et al. (42), Uehara et al. (18), Papaspyridakos et al. (20), Cruz et al. (22) | 25 |
| Pohl et al. (43) | Emfietzoglou et al. (44), Grunau et al. (2), Medikeri et al. (31), Bitinas et al. (4), Yu et al. (32), Chaware et al. (5), Iezzi et al. (8), Vazouras et al. (9), Mokcheh et al. (10), Nielsen et al. (33), Altaib et al. (11), Ravidà et al. (12), Chen et al. (14), Bitaraf et al. (15), Ravidà et al. (16), Yan et al. (42), Aldawood et al. (34), Papaspyridakos et al. (20), Cruz et al. (22), de Souza et al. (45) | 20 |
| Bolle et al. (46) | Abayov et al. (37), Kermanshah et al. (39), Grunau et al. (2), Zhang et al. (47), Toledano et al. (40), Terheyden et al. (3), Moraschini et al. (48), Bitinas et al. (4), Yu et al. (32), Chaware et al. (5), Abdel-Halim et al. (6), Carosi et al. (41), Carosi et al. (49), Lozano-Carrascsl et al. (7), Iezzi et al. (8), Vazouras et al. (9), Ravidà et al. (12), Ravidà et al. (16), Yan et al. (42), Starch-Jensen et al. (21) | 20 |
| Felice et al. (50) | Alemán et al. (51), Zhang et al. (38), Kermanshah et al. (39), Mester et al. (52), Grunau et al. (2), Wang et al. (53), Medikeri et al. (31), Toledano et al. (40), Terheyden et al. (3), Bitinas et al. (4), Yu et al. (32), Abdel-Halim et al. (6), Carosi et al. (41), Carosi et al. (49), Xu et al. (54), Iezzi et al. (8), Esposito et al. (55) | 17 |
| Thoma et al. (56) | Alemán et al. (51), Zhang et al. (38), Emfietzoglou et al. (44), Kermanshah et al. (39), Mester et al. (52), Grunau et al. (2), Wang et al. (53), Medikeri et al. (31), Toledano et al. (40), Yu et al. (32), Chaware et al. (5), Abdel-Halim et al. (6), Carosi et al. (41), Xu et al. (54), Lozano-Carrascal et al. (7), Xu et al. (57), Iezzi et al. (8) | 17 |
| Felice et al. (58) | Abayov et al. (37), Alemán et al. (51), Zhang et al. (38), Kermanshah et al. (39), Mester et al. (52), Grunau et al. (2), Wang et al. (53), Toledano et al. (40), Terheyden et al. (3), Yu et al. (32), Chaware et al. (5), Abdel-Halim et al. (6), Carosi et al. (41), Carosi et al. (49), Xu et al. (54), Iezzi et al. (8), Esposito et al. (55) | 17 |
| Esposito et al. (59) | Grunau et al. (2), Terheyden et al. (3), Bitinas et al. (4), Yu et al. (32), Chaware et al. (5), Altaib et al. (11), Amine et al. (13), Bitaraf et al. (15), de N Dias et al. (17), Uehara et al. (18), Fan et al. (23), Tong et al. (24), Toti et al. (25), Thoma et al. (28), Lee et al. (60), Monje et al. (61) | 16 |
| Felice et al. (62) | Grunau et al. (2), Bitinas et al. (4), Yu et al. (32), Chaware et al. (5), Iezzi et al. (8), Vazouras et al. (9), Mokcheh et al. (10), Altaib et al. (11), Ravidà et al. (12), Amine et al. (13), Chen et al. (14), Bitaraf et al. (15), Ravidà et al. (16), Uehara et al. (18), Palacios et al. (19), Papaspyridakos et al. (20) | 16 |
| Guljé et al. (63) | Grunau et al. (2), Bitinas et al. (4), Yu et al. (32), Chaware et al. (5), Lozano-Carrascsl et al. (7), Xu et al. (57), Mokcheh et al. (10), Altaib et al. (11), Amine et al. (13), Chen et al. (14), Bitaraf et al. (15), Yan et al. (42), Palacios et al. (19), Cruz et al. (22), de Souza et al. (45), Thoma et al. (28) | 16 |
| Esposito et al. (64) | Alemán et al. (51), Zhang et al. (38), Kermanshah et al. (39), Mester et al. (52), Grunau et al. (2), Wang et al. (53), Toledano et al. (40), Terheyden et al. (3), Yu et al. (32), Abdel-Halim et al. (6), Carosi et al. (41), Carosi et al. (49), Xu et al. (54), Iezzi et al. (8), Esposito et al. (55) | 15 |
| Rossi et al. (65) | Emfietzoglou et al. (44), Kermanshah et al. (39), Medikeri et al. (31), Guida et al. (66), Yu et al. (32), Abdel-Halim et al. (6), Xu et al. (54), Xu et al. (57), Vazouras et al. (9), Bitaraf et al. (15), Ravidà et al. (16), Aldawood et al. (34), Papaspyridakos et al. (20), de Souza et al. (45), Lemos et al. (26) | 15 |
| Felice et al. (67) | Abayov et al. (37), Medikeri et al. (31), Terheyden et al. (3), Bitinas et al. (4), Iezzi et al. (8), Altaib et al. (11), Esposito et al. (55), Amine et al. (13), Bitaraf et al. (15), Palacios et al. (19), Starch-Jensen et al. (21), Tong et al. (24), Lemos et al. (26), Camps-Font et al. (27), Nisand et al. (29) | 15 |
| Gastaldi et al. (68) | Grunau et al. (2), Medikeri et al. (31), Terheyden et al. (3), Bitinas et al. (4), Yu et al. (32), Chaware et al. (5), Lozano-Carrascsl et al. (7), Iezzi et al. (8), Vazouras et al. (9), Ravidà et al. (12), Chen et al. (14), Ravidà et al. (16), Yan et al. (42), Starch-Jensen et al. (21) | 14 |
| Gastaldi et al. (69) | Zhang et al. (38), Kermanshah et al. (39), Grunau et al. (2), Toledano et al. (40), Bitinas et al. (4), Yu et al. (32), Chaware et al. (5), Abdel-Halim et al. (6), Carosi et al. (41), Iezzi et al. (8), Ravidà et al. (12), Ravidà et al. (16), Yan et al. (42), Cruz et al. (22) | 14 |
| Guljé et al. (70) | Emfietzoglou et al. (44), Medikeri et al. (31), Guida et al. (66), Yu et al. (32), Abdel-Halim et al. (6), Vazouras et al. (9), Bitaraf et al. (15), Ravidà et al. (16), Aldawood et al. (34), Papaspyridakos et al. (20), Tong et al. (24), Lemos et al. (26), Lee et al. (60), Monje et al. (61) | 14 |
| Guljé et al. (71) | Alemán et al. (51), Emfietzoglou et al. (44), Kermanshah et al. (39), Mester et al. (52), Grunau et al. (2), Wang et al. (53), Toledano et al. (40), Bitinas et al. (4), Yu et al. (32), Abdel-Halim et al. (6), Carosi et al. (41), Xu et al. (54) | 12 |
| Schincaglia et al. (72) | Emfietzoglou et al. (44), Grunau et al. (2), Medikeri et al. (31), Toledano et al. (40), Bitinas et al. (4), Yu et al. (32), Chaware et al. (5), Iezzi et al. (8), Ravidà et al. (12), Amine et al. (13), Bitaraf et al. (15), Ravidà et al. (16) | 12 |
| Esposito et al. (73) | Liang et al. (74), Kermanshah et al. (39), Liang et al. (75), Bitinas et al. (4), Chaware et al. (5), Abdel-Halim et al. (6), Mokcheh et al. (10), Altaib et al. (11), Amine et al. (13), Bitaraf et al. (15), Palacios et al. (19), Cruz et al. (22) | 12 |
| Thoma et al. (76) | Iezzi et al. (8), Mokcheh et al. (10), Ravidà et al. (12), Ravidà et al. (16), Uehara et al. (18), Palacios et al. (19), Fan et al. (23), Tong et al. (24), Lemos et al. (26), Thoma et al. (28) | 10 |
| Cannizzaro et al. (77) | Kermanshah et al. (39), Tang et al. (78), Abdel-Halim et al. (6), Amine et al. (13), Chen et al. (14), Bitaraf et al. (15), Fan et al. (23), Tong et al. (24), Lemos et al. (26), Lee et al. (60) | 10 |
| Shi et al. (79) | Emfietzoglou et al. (44), Kermanshah et al. (39), Medikeri et al. (31), Toledano et al. (40), Bitinas et al. (4), Yu et al. (32), Abdel-Halim et al. (6), Carosi et al. (41), Iezzi et al. (8) | 9 |
| Felice et al. (80) | Kermanshah et al. (39), Zhang et al. (47), Guida et al. (66), Moraschini et al. (48), Bitinas et al. (4), Vazouras et al. (9), Altaib et al. (11), Bitaraf et al. (15), Papaspyridakos et al. (20) | 9 |
| Felice et al. (81) | Grunau et al. (2), Terheyden et al. (3), Bitinas et al. (4), Yu et al. (32), Chaware et al. (5), Iezzi et al. (8), Ravidà et al. (12), Ravidà et al. (16), Yan et al. (42) | 9 |
| Rokn et al. (82) | Emfietzoglou et al. (44), Kermanshah et al. (39), Zhang et al. (47), Terheyden et al. (3), Moraschini et al. (48), Bitinas et al. (4), Yu et al. (32), Carosi et al. (49), Iezzi et al. (8) | 9 |
| Esposito et al. (83) | Terheyden et al. (3), Bitinas et al. (4), Iezzi et al. (8), Altaib et al. (11), Amine et al. (13), Bitaraf et al. (15), Tong et al. (24), Toti et al. (25), Lee et al. (60) | 9 |
| Guida et al. (84) | Liang et al. (74), Kermanshah et al. (39), Rosa et al. (85), Liang et al. (75), Medikeri et al. (31), Guida et al. (66), Yu et al. (32), Abdel-Halim et al. (6) | 8 |
| Naenni et al. (86) | Emfietzoglou et al. (44), Kermanshah et al. (39), Medikeri et al. (31), Yu et al. (32), Xu et al. (54), Xu et al. (57), Vazouras et al. (9), Ravidà et al. (16) | 8 |
| Felice et al. (87) | Terheyden et al. (3), Iezzi et al. (8), Amine et al. (13), Chen et al. (14), Bitaraf et al. (15), de N Dias et al. (17), Uehara et al. (18), Toti et al. (25) | 8 |
| Zadeh et al. (88) | Emfietzoglou et al. (44), Kermanshah et al. (39), Medikeri et al. (31), Guida et al. (66), Yu et al. (32), Abdel-Halim et al. (6) | 6 |
| Romeo et al. (89) | Medikeri et al. (31), Guida et al. (66), Yu et al. (32), Bitaraf et al. (15), Ravidà et al. (16), Lemos et al. (26) | 6 |
| Cannizzaro et al. (90) | Rosa et al. (85), Guida et al. (66), Yu et al. (32), Wu et al. (91), Bitaraf et al. (15), Ravidà et al. (16) | 6 |
| Cannizzaro et al. (92) | Liang et al. (74), Kermanshah et al. (39), Liang et al. (75), Guida et al. (66), Wu et al. (91), Abdel-Halim et al. (6) | 6 |
| Weerapong et al. (93) | Kermanshah et al. (39), Guida et al. (66), Yu et al. (32), Wu et al. (91), Abdel-Halim et al. (6), Xu et al. (57) | 6 |
| Yu et al. (94) | Zhang et al. (38), Kermanshah et al. (39), Tang et al. (78), Abdel-Halim et al. (6), Iezzi et al. (8), Bitaraf et al. (15) | 6 |
| Shah et al. (95) | Abayov et al. (37), Kermanshah et al. (39), Medikeri et al. (31), Terheyden et al. (3), Yu et al. (32), Iezzi et al. (8) | 6 |
| Bernardi et al. (96) | Abayov et al. (37), Terheyden et al. (3), Bitinas et al. (4), Yu et al. (32), Abdel-Halim et al. (6), Starch-Jensen et al. (21) | 6 |
| Storelli et al. (97) | Kermanshah et al. (39), Medikeri et al. (31), Guida et al. (66), Abdel-Halim et al. (6), Xu et al. (54) | 5 |
| Felice et al. (98) | Kermanshah et al. (39), Medikeri et al. (31), Terheyden et al. (3), Xu et al. (54), Iezzi et al. (8) | 5 |
| Nielsen et al. (99) | Abayov et al. (37), Zhang et al. (38), Emfietzoglou et al. (44), Grunau et al. (2), Toledano et al. (40) | 5 |
| Shi et al. (100) | Zhang et al. (38), Emfietzoglou et al. (44), Toledano et al. (40), Tang et al. (78) | 4 |
| Sahrmann et al. (101) | Medikeri et al. (31), Yu et al. (32), Ravidà et al. (16), Papaspyridakos et al. (20) | 4 |
| Hadzik et al. (102) | Emfietzoglou et al. (44), Grunau et al. (2), Lozano-Carrascal et al. (7), Iezzi et al. (8) | 4 |
| Esposito et al. (103) | Chaware et al. (5), Mokcheh et al. (10), Bitaraf et al. (15), Cruz et al. (22) | 4 |
| Zhang et al. (104) | Chaware et al. (5), Abdel-Halim et al. (6), Mokcheh et al. (10), Cruz et al. (22) | 4 |
| Taschieri et al. (105) | Chaware et al. (5), Abdel-Halim et al. (6), Cruz et al. (22) | 4 |
| Hadzik et al. (106) | Alemán et al. (51), Zhang et al. (38), Emfietzoglou et al. (44), Medikeri et al. (31) | 4 |
| Rossi et al. (107) | Grunau et al. (2), Zhang et al. (47), Toledano et al. (40) | 3 |
| Calvo-Guirado et al. (108) | Zhang et al. (47), Moraschini et al. (48), Abdel-Halim et al. (6) | 3 |
| Guljé et al. (109) | Guida et al. (66), Yu et al. (32) | 3 |
| Felice et al. (35) | Chaware et al. (5), Mokcheh et al. (10), Thoma et al. (28) | 3 |
| Felice et al. (110) | Chaware et al. (5), Mokcheh et al. (10), Thoma et al. (28) | 3 |
| Stellingsma et al. (111) | Liang et al. (74), Liang et al. (75), Altaib et al. (11) | 3 |
| Queiroz et al. (112) | Abdel-Halim et al. (6), Aldawood et al. (34), Lemos et al. (26) | 3 |
| Rossi et al. (113) | Monje et al. (61), Mezzomo et al. (114), Monje et al. (115) | 3 |
| Romeo et al. (116) | Monje et al. (61), Monje et al. (115), Kotsovilis et al. (117) | 3 |
| Ferrigno et al. (118) | Abdel-Halim et al. (6), Lemos et al. (26), Monje et al. (115) | 3 |
| Nedir et al. (119) | Abdel-Halim et al. (6), Mezzomo et al. (114), Monje et al. (115) | 3 |
| Deporter et al. (120) | Abdel-Halim et al. (6), Mezzomo et al. (114), Kotsovilis et al. (117) | 3 |
| Guljé et al. (121) | Alemán et al. (51), Abayov et al. (37), Zhang et al. (47), | 3 |
| Barausse et al. (122) | Zhang et al. (47), Guida et al. (66) | 2 |
| Cannizzaro et al. (123) | Amine et al. (13), Bitaraf et al. (15) | 2 |
| Esposito et al. (124) | Bitaraf et al. (15), Lemos et al. (26) | 2 |
| Felice et al. (125) | Toti et al. (25), Camps-Font et al. (27) | 2 |
| Felice et al. (126) | Toti et al. (25), Camps-Font et al. (27) | 2 |
| Bianchi et al. (127) | Toti et al. (25), Camps-Font et al. (27) | 2 |
| Chiapasco et al. (128) | Toti et al. (25), Camps-Font et al. (27) | 2 |
| Esposito et al. (129) | Mokcheh et al. (10), Thoma et al. (28) | 2 |
| Cannizzaro et al. (130) | Wu et al. (91), Mezzomo et al. (114) | 2 |
| Perelli et al. (131) | Vazouras et al. (9), Mezzomo et al. (114) | 2 |
| Telleman et al. (132) | Monje et al. (61), Mezzomo et al. (114) | 2 |
| Romeo et al. (133) | Mezzomo et al. (114), Kotsovilis et al. (117) | 2 |
| Brocard et al. (134) | Mezzomo et al. (114), Kotsovilis et al. (117) | 2 |
| Strietzel et al. (135) | Mezzomo et al. (114), Kotsovilis et al. (117) | 2 |
| Amato et al. (136) | Wu et al. (91), Abdel-Halim et al. (6) | 2 |
| Anitua et al. (137) | Wu et al. (91), Abdel-Halim et al. (6) | 2 |
| Anitua et al. (138) | Wu et al. (91), Abdel-Halim et al. (6) | 2 |
| Arlin (139) | Abdel-Halim et al. (6), Lemos et al. (26) | 2 |
| Degidi et al. (140) | Wu et al. (91), Abdel-Halim et al. (6) | 2 |
| Cochran et al. (141) | Abdel-Halim et al. (6), Kotsovilis et al. (117) | 2 |
| Hallman (142) | Abdel-Halim et al. (6), Monje et al. (115) | 2 |
| Malmstrom et al. (143) | Abdel-Halim et al. (6), Vazouras et al. (9) | 2 |
| McGlumphy et al. (144) | Abdel-Halim et al. (6), Monje et al. (115) | 2 |
| Polizzi et al. (145) | Abdel-Halim et al. (6), Monje et al. (115) | 2 |
| Roccuzzo et al. (146) | Abdel-Halim et al. (6), Kotsovilis et al. (117) | 2 |
| Roccuzzo et al. (147) | Abdel-Halim et al. (6), Kotsovilis et al. (117) | 2 |
| Tawil et al. (148) | Abdel-Halim et al. (6), Mezzomo et al. (114) | 2 |
| Testori et al. (149) | Abdel-Halim et al. (6), Monje et al. (115) | 2 |
| Barausse et al. (150) | Zhang et al. (38), Zhang et al. (47), | 2 |
| Magdy et al. (151) | Zhang et al. (38), Toledano et al. (40) | 2 |

**References**

1. Pistilli R, Felice P, Cannizzaro G, Piatelli M, Corvino V, Barausse C, et al. Posterior atrophic jaws rehabilitated with prostheses supported by 6 mm long 4 mm wide implants or by longer implants in augmented bone. One-year post-loading results from a pilot randomised controlled trial. Eur J Oral Implantol. 2013;6(4):359-72. https://europepmc.org/article/med/24570981

2. Grunau O, Terheyden H. Lateral augmentation of the sinus floor followed by regular implants versus short implants in the vertically deficient posterior maxilla: a systematic review and timewise meta-analysis of randomized studies. Int J Oral Maxillofac Surg. 2023;52(7):813-24. doi: 10.1016/j.ijom.2022.11.015

3. Terheyden H, Meijer GJ, Raghoebar GM. Vertical bone augmentation and regular implants versus short implants in the vertically deficient posterior mandible: a systematic review and meta-analysis of randomized studies. Int J Oral Maxillofac Surg. 2021;50(9):1249-58. doi: 10.1016/j.ijom.2021.01.005

4. Bitinas D, Bardijevskyt G. Short implants without bone augmentation vs. long implants with bone augmentation: systematic review and meta-analysis. Aust Dent J. 2021;66(S1):S71-81. doi: 10.1111/adj.12859

5. Chaware S, Thakare V, Chaudhary R, Jankar A, Thakkar S, Borse S. The rehabilitation of posterior atrophic maxilla by using the graftless option of short implant versus conventional long implant with sinus graft: A systematic review and meta-analysis of randomized controlled clinical trial. J Indian Prosthodont Soc. 2021;21(1):28-44. doi: 10.4103/jips.jips_400_20

6. Abdel-Halim M, Issa D, Chrcanovic BR, nueva E a sitio externo E enlace se abrirá en una ventana. The Impact of Dental Implant Length on Failure Rates: A Systematic Review and Meta-Analysis. Materials. 2021;14(14):3972. doi: 10.3390/ma14143972

7. Lozano-Carrascal N, Anglada-Bosqued A, Salomó-Coll O, Hernández-Alfaro F, Wang HL, Gargallo-Albiol J. Short implants (<8mm) versus longer implants (≥8mm) with lateral sinus floor augmentation in posterior atrophic maxilla: A meta-analysis of RCT`s in humans. Med Oral Patol Oral Cirugia Bucal. 2020;25(2):e168-79. doi: 10.4317/medoral.23248

8. Iezzi G, Perrotti V, Felice P, Barausse C, Piattelli A, Del Fabbro M. Are <7-mm long implants in native bone as effective as longer implants in augmented bone for the rehabilitation of posterior atrophic jaws? A systematic review and meta-analysis. Clin Implant Dent Relat Res. 2020;22(5):552-66. doi: 10.1111/cid.12946

9. Vazouras K, de Souza AB, Gholami H, Papaspyridakos P, Pagni S, Weber HP. Effect of time in function on the predictability of short dental implants (≤6 mm): A meta-analysis. J Oral Rehabil. 2020;47(3):403-15. doi: 10.1111/joor.12925

10. Mokcheh A, Jegham H, Turki S. Short implants as an alternative to sinus lift for the rehabilitation of posterior maxillary atrophies: Systematic review and meta-analysis. J Stomatol Oral Maxillofac Surg. 2019;120(1):28-37. doi: 10.1016/j.jormas.2018.11.006

11. Altaib FH, Alqutaibi AY, Al-Fahd A, Eid S. Short dental implant as alternative to long implant with bone augmentation of the atrophic posterior ridge: A systematic review and meta-analysis of RCTs. Quintessence Int. 2019;50(8):636-51. doi: 10.3290/j.qi.a42948

12. Ravidà A, Wang IC, Sammartino G, Barootchi S, Tattan M, Troiano G, et al. Prosthetic Rehabilitation of the Posterior Atrophic Maxilla, Short (≤6 mm) or Long (≥10 mm) Dental Implants? A Systematic Review, Meta-analysis, and Trial Sequential Analysis: Naples Consensus Report Working Group A. Implant Dent. 2019;28(6):590-602. doi: 10.1097/ID.0000000000000919

13. Amine M, Guelzim Y, Benfaida S, Bennani A, Andoh A. Short implants (5–8 mm) vs. long implants in augmented bone and their impact on peri-implant bone in maxilla and/or mandible: Systematic review. J Stomatol Oral Maxillofac Surg. 2019;120(2):133-42. doi: 10.1016/j.jormas.2018.11.007

14. Chen S, Ou Q, Wang Y, Lin X. Short implants (5‐8 mm) vs long implants (≥10 mm) with augmentation in atrophic posterior jaws: A meta‐analysis of randomised controlled trials. J Oral Rehabil. 2019;46(12):1192-203. doi: 10.1111/joor.12860

15. Bitaraf T, Keshtkar A, Rokn AR, Monzavi A, Geramy A, Hashemi K. Comparing short dental implant and standard dental implant in terms of marginal bone level changes: A systematic review and meta-analysis of randomized controlled trials. Clin Implant Dent Relat Res. 2019;21(4):796-812. doi: 10.1111/cid.12774

16. Ravidà A, Wang IC, Barootchi S, Askar H, Tavelli L, Gargallo-Albiol J, et al. Meta-analysis of randomized clinical trials comparing clinical and patient-reported outcomes between extra-short (≤6 mm) and longer (≥10 mm) implants. J Clin Periodontol. 2019;46(1):118-42. doi: 10.1111/jcpe.13026

17. de N Dias FJ, Pecorari VGA, Martins CB, Del Fabbro M, Casati MZ. Short implants versus bone augmentation in combination with standard-length implants in posterior atrophic partially edentulous mandibles: systematic review and meta-analysis with the Bayesian approach. Int J Oral Maxillofac Surg. 2019;48(1):90-6. doi: 10.1016/j.ijom.2018.05.009

18. Uehara PN, Matsubara VH, Igai F, Sesma N, Mukai MK, Araujo MG. Short dental implants (≤7mm) versus longer implants in augmented bone area: A meta-analysis of randomized controlled trials. Open Dent J. 2018;12(1):354-65. doi: 10.2174/1874210601812010354

19. Palacios JAV, Garcia JJ, Caramês JMM, Quirynen M, da Silva Marques DN. Short implants versus bone grafting and standard-length implants placement: a systematic review. Clin Oral Investig. 2018;22(1):69-80. doi: 10.1007/s00784-017-2205-0

20. Papaspyridakos P, De Souza A, Vazouras K, Gholami H, Pagni S, Weber HP. Survival rates of short dental implants (≤6 mm) compared with implants longer than 6 mm in posterior jaw areas: A meta‐analysis. Clin Oral Implants Res. 2018;29(S16):8-20. doi: 10.1111/clr.13289

21. Starch-Jensen T, Nielsen HB. Prosthetic Rehabilitation of the Partially Edentulous Atrophic Posterior Mandible with Short Implants (≤ 8 mm) Compared with the Sandwich Osteotomy and Delayed Placement of Standard Length Implants (> 8 mm): a Systematic Review. J Oral Maxillofac Res. 2018;9(2):e2. doi: 10.5037/jomr.2018.9202

22. Cruz RS, de Araújo Lemos CA, de Souza Batista VE, e Oliveira HFF, de Luna Gomes JM, Pellizzer EP, et al. Short implants versus longer implants with maxillary sinus lift. A systematic review and meta-analysis. Braz Oral Res. 2018;32:e86. doi: 10.1590/1807-3107bor-2018.vol32.0086

23. Fan T, Li Y, Deng WW, Wu T, Zhang W. Short Implants (5 to 8 mm) Versus Longer Implants (>8 mm) with Sinus Lifting in Atrophic Posterior Maxilla: A Meta-Analysis of RCTs. Clin Implant Dent Relat Res. 2017;19(1):207-15. doi: 10.1111/cid.12432

24. Tong Q, Zhang X, Yu L. Meta-analysis of randomized controlled trials comparing clinical outcomes between short implants and long implants with bone augmentation procedure. Int J Oral Maxillofac Implants. 2017;32(1):e25-34. doi: 10.11607/jomi.4793

25. Toti P, Marchionni S, Menchini-Fabris GB, Marconcini S, Covani U, Barone A. Surgical techniques used in the rehabilitation of partially edentulous patients with atrophic posterior mandibles: A systematic review and meta-analysis of randomized controlled clinical trials. J Cranio-Maxillo-fac Surg Off Publ Eur Assoc Cranio-Maxillo-fac Surg. 2017;45(8):1236-45. doi: 10.1016/j.jcms.2017.04.011

26. Lemos CAA, Ferro-Alves ML, Okamoto R, Mendonça MR, Pellizzer EP. Short dental implants versus standard dental implants placed in the posterior jaws: A systematic review and meta-analysis. J Dent. 2016;47:8-17. doi: 10.1016/j.jdent.2016.01.005

27. Camps-Font O, Burgueño-Barris G, Figueiredo R, Jung RE, Gay-Escoda C, Valmaseda-Castellón E. Interventions for Dental Implant Placement in Atrophic Edentulous Mandibles: Vertical Bone Augmentation and Alternative Treatments. A Meta-Analysis of Randomized Clinical Trials. J Periodontol. 2016;87(12):1444-57. doi: 10.1902/jop.2016.160226

28. Thoma DS, Zeltner M, Hüsler J, Hämmerle CHF, Jung RE. EAO Supplement Working Group 4 - EAO CC 2015 Short implants versus sinus lifting with longer implants to restore the posterior maxilla: A systematic review. Clin Oral Implants Res. 2015;26:154-69. doi: 10.1111/clr.12615

29. Nisand D, Picard N, Rocchietta I. Short implants compared to implants in vertically augmented bone: a systematic review. Clin Oral Implants Res. 2015;26(S11):170-9. doi: 10.1111/clr.12632

30. Esposito M, Pistilli R, Barausse C, Felice P. Three-year results from a randomised controlled trial comparing prostheses supported by 5-mm long implants or by longer implants in augmented bone in posterior atrophic edentulous jaws. Eur J Oral Implantol. 2014;7(4):383-95. https://pubmed.ncbi.nlm.nih.gov/25422826/

31. Medikeri RS, Pereira MA, Waingade M, Navale S. Survival of surface-modified short versus long implants in complete or partially edentulous patients with a follow-up of 1 year or more: a systematic review and meta-analysis. J Periodontal Implant Sci. 2022;52(2):261-81. doi: 10.5051/jpis.2007340367

32. Yu X, Ruogu X, Zhengchuan Z, Yang Y, Feilong D. A meta-analysis indicating extra-short implants (≤ 6 mm) as an alternative to longer implants (≥ 8 mm) with bone augmentation. Sci Rep Nat Publ Group. 2021;11(1):8152. doi: 10.1038/s41598-021-87507-1

33. Nielsen HB, Schou S, Isidor F, Christensen AE, Starch-Jensen T. Short implants (≤8mm) compared to standard length implants (>8mm) in conjunction with maxillary sinus floor augmentation: a systematic review and meta-analysis. Int J Oral Maxillofac Surg. 2019;48(2):239-49. doi: 10.1016/j.ijom.2018.05.010

34. Aldawood T, Qarni M, Alhayek A, Muslih W, Alfantoukh A, Albeladi R, et al. Comparison between short dental implants versus standard dental implants of posterior jaws: A systematic review & meta-analysis. World J Pharm Res. 2019;8:1501-13. https://wjpr.s3.ap-south-1.amazonaws.com/article_issue/1586583733.pdf

35. Felice P, Pistilli R, Piattelli M, Soardi E, Corvino V, Esposito M. Posterior atrophic jaws rehabilitated with prostheses supported by 5 x 5 mm implants with a novel nanostructured calcium-incorporated titanium surface or by longer implants in augmented bone. One-year results from a randomised controlled trial. Eur J Oral Implantol. 2012;5(2):149-61. https://pubmed.ncbi.nlm.nih.gov/22866291/

36. Bechara S, Kubilius R, Veronesi G, Pires JT, Shibli JA, Mangano FG. Short (6-mm) dental implants versus sinus floor elevation and placement of longer (≥10-mm) dental implants: a randomized controlled trial with a 3-year follow-up. Clin Oral Implants Res. 2017;28(9):1097-107. doi: 10.1111/clr.12923

37. Abayov P, Sarikov R, Nazarenko LM, Babich O, Haimov E, Juodzbalys G. Outcome Difference between Short and Longer Dental Implants Placed Simultaneously with Alveolar Bone Augmentation: a Systematic Review and Meta-Analysis. J Oral Maxillofac Res.;15(2):e2. doi: 10.5037/jomr.2024.15202

38. Zhang Y, Tang X, Zhang Y, Cao C. A network meta-analysis comparing treatment modalities of short and long implants in the posterior maxilla with insufficient bone height. BMC Oral Health 2024;24(1). doi: 10.1186/s12903-024-05377-1

39. Kermanshah H, Keshtkar A, Hassani A, Bitaraf T. Comparing short implants to standard dental implants: a systematic review and meta-analysis of randomized controlled trials with extended follow-up. Evid Based Dent. 2023;24(4):192-3. doi: 10.1038/s41432-023-00924-1

40. Toledano M, Fernández-Romero E, Vallecillo C, Toledano R, Osorio MT, Vallecillo-Rivas M. Short versus standard implants at sinus augmented sites: a systematic review and meta-analysis. Clin Oral Investig. 2022;26(11):6681-98. doi: 10.1007/s00784-022-04628-1

41. Carosi P, Lorenzi C, Lio F, Laureti M, Ferrigno N, Arcuri C. Short implants (≤6mm) as an alternative treatment option to maxillary sinus lift. Int J Oral Maxillofac Surg. 2021;50(11):1502-10. doi: 10.1016/j.ijom.2021.02.014

42. Yan Q, Wu X, Su M, Hua F, Shi B. Short implants (≤6 mm) versus longer implants with sinus floor elevation in atrophic posterior maxilla: A systematic review and meta-analysis. BMJ Open. 2019;9(10). doi: 10.1136/bmjopen-2019-029826

43. Pohl V, Thoma DS, Sporniak-Tutak K, Garcia-Garcia A, Taylor TD, Haas R, et al. Short dental implants (6 mm) versus long dental implants (11-15 mm) in combination with sinus floor elevation procedures: 3-year results from a multicentre, randomized, controlled clinical trial. J Clin Periodontol. 2017;44(4):438-45. doi: 10.1111/jcpe.12694

44. Emfietzoglou R, Dereka X. Survival Rates of Short Dental Implants (≤6 mm) Used as an Alternative to Longer (>6 mm) Implants for the Rehabilitation of Posterior Partial Edentulism: A Systematic Review of RCTs. Dent J. 2024;12(6). doi: 10.3390/dj12060185

45. Tolentino da Rosa de Souza P, Binhame Albini Martini M, Reis Azevedo-Alanis L. Do short implants have similar survival rates compared to standard implants in posterior single crown?: A systematic review and meta-analysis. Clin Implant Dent Relat Res. 2018;20(5):890-901. doi: doi.org/10.1111/cid.12634

46. Bolle C, Felice P, Barausse C, Pistilli V, Trullenque-Eriksson A, Esposito M. 4 mm long vs longer implants in augmented bone in posterior atrophic jaws: 1-year post-loading results from a multicentre randomised controlled trial. Eur J Oral Implantol. 2018;11(1):31-47. https://europepmc.org/article/med/29557399

47. 启航张, 佳明龚, 佳颖余, 瑞敏赵, 萍苟, 占海余. 在后牙区应用4 mm超短种植体临床效果的Meta分析. West China J Stomatol. 2023;41(1):80. doi: 10.7518/hxkq.2023.01.011

48. Moraschini V, Mourão CF de AB, Montemezzi P, Kischinhevsky ICC, de Almeida DCF, Javid K, et al. Clinical Comparation of Extra-Short (4 mm) and Long (>8 mm) Dental Implants Placed in Mandibular Bone: A Systematic Review and Metanalysis. Healthc Basel Switz. 2021;9(3):315. doi: 10.3390/healthcare9030315

49. Carosi P, Lorenzi C, Laureti M, Ferrigno N, Arcuri C. Short Dental Implants (≤ 6 mm) to Rehabilitate Severe Mandibular Atrophy: A Systematic Review. Int J Oral Maxillofac Implants. 2021;36(1):30-7. doi: 10.11607/jomi.8510

50. Felice P, Barausse C, Pistilli R, Ippolito DR, Esposito M. Five-year results from a randomised controlled trial comparing prostheses supported by 5-mm long implants or by longer implants in augmented bone in posterior atrophic edentulous jaws. Int J Oral Implantol Berl Ger. 2019;12(1):25-37. https://europepmc.org/article/med/31116186

51. Alemán BO, Rivera-Velazquez I, Jana-Hernández Z, Rivas-Tumanyan S, Guerrero-Rodríguez LM, Elias-Boneta AR. Long-Term Outcomes of Short versus Long Dental Implants with Sinus Lift in Atrophied Posterior Maxillae: A Systematic Review and Meta-Analysis. P R Health Sci J. 17 de marzo de 2025;44(1):54-62. https://prhsj.rcm.upr.edu/index.php/prhsj/article/view/3472

52. Mester A, Onisor F, Stasio DD, Piciu A, Cosma AM, Bran S. Short Implants versus Standard Implants and Sinus Floor Elevation in Atrophic Posterior Maxilla: A Systematic Review and Meta-Analysis of Randomized Clinical Trials with ≥5 Years’ Follow-Up. J Pers Med. 2023;13(2):169. doi: 10.3390/jpm13020169

53. Wang M, Liu F, Ulm C, Shen H, Rausch-Fan X. Short Implants versus Longer Implants with Sinus Floor Elevation: A Systemic Review and Meta-Analysis of Randomized Controlled Trials with a Post-Loading Follow-Up Duration of 5 Years. Materials. 2022;15(13):4722. doi: 10.3390/ma15134722

54. Xu X, Huang J, Fu X, Kuang Y, Yue H, Song J, et al. Short implants versus longer implants in the posterior alveolar region after an observation period of at least five years: A systematic review and meta-analysis. J Dent. 2020;100:103386. doi: 10.1016/j.jdent.2020.103386

55. Esposito M, Buti J, Barausse C, Gasparro R, Sammartino G, Felice P. Short implants versus longer implants in vertically augmented atrophic mandibles: A systematic review of randomised controlled trials with a 5-year post-loading follow-up. Int J Oral Implantol. 2019;12(3):267-80. https://europepmc.org/article/med/31535097

56. Thoma DS, Haas R, Sporniak-Tutak K, Garcia A, Taylor TD, Hämmerle CHF. Randomized controlled multicentre study comparing short dental implants (6 mm) versus longer dental implants (11-15 mm) in combination with sinus floor elevation procedures: 5-Year data. J Clin Periodontol. 2018;45(12):1465-74. doi: 10.1111/jcpe.12323

57. Xu X, Hu B, Xu Y, Liu Q, Ding H, Xu L. Short versus standard implants for single-crown restorations in the posterior region: A systematic review and meta-analysis. J Prosthet Dent. 2020;124(5):530-8. doi: 10.1016/j.prosdent.2019.09.030

58. Felice P, Pistilli R, Barausse C, Piattelli M, Buti J, Esposito M. Posterior atrophic jaws rehabilitated with prostheses supported by 6-mm-long 4-mm-wide implants or by longer implants in augmented bone. Five-year post-loading results from a within-person randomised controlled trial. Int J Oral Implantol Berl Ger. 2019;12(1):57-72. https://europepmc.org/article/med/31116188

59. Esposito M, Pellegrino G, Pistilli R, Felice P. Rehabilitation of postrior atrophic edentulous jaws: prostheses supported by 5 mm short implants or by longer implants in augmented bone? One-year results from a pilot randomised clinical trial. Eur J Oral Implantol. 2011;4(1):21-30. https://europepmc.org/article/med/21594216

60. Lee SA, Lee CT, Fu MM, Elmisalati W, Chuang SK. Systematic review and meta-analysis of randomized controlled trials for the management of limited vertical height in the posterior region: Short implants (5 to 8 mm) vs longer implants (> 8 mm) in vertically augmented sites. Int J Oral Maxillofac Implants. 2014;29(5):1085-97. doi: 10.11607/jomi.3504

61. Monje A, Suarez F, Galindo-Moreno P, García-Nogales A, Fu JH, Wang HL. A systematic review on marginal bone loss around short dental implants (<10 mm) for implant-supported fixed prostheses. Clin Oral Implants Res. 2014;25(10):1119-24. doi: 10.1111/clr.12236

62. Felice P, Pistilli R, Barausse C, Bruno V, Trullenque-Eriksson A, Esposito M. Short implants as an alternative to crestal sinus lift: A 1-year multicentre randomised controlled trial. Eur J Oral Implantol. 2015;8(4):375-84. https://pubmed.ncbi.nlm.nih.gov/26669547/

63. Guljé FL, Raghoebar GM, Vissink A, Meijer HJA. Single crowns in the resorbed posterior maxilla supported by either 6-mm implants or by 11-mm implants combined with sinus floor elevation surgery: a 1-year randomised controlled trial. Eur J Oral Implantol. 2014;7(3):247-55. doi: https://pubmed.ncbi.nlm.nih.gov/25237669/

64. Esposito M, Barausse C, Pistilli R, Piattelli M, Di Simone S, Ippolito DR, et al. Posterior atrophic jaws rehabilitated with prostheses supported by 5 × 5 mm implants with a nanostructured calcium-incorporated titanium surface or by longer implants in augmented bone. Five-year results from a randomised controlled trial. Int J Oral Implantol Berl Ger. 2019;12(1):39-54. https://europepmc.org/article/med/31116187

65. Rossi F, Botticelli D, Cesaretti G, De Santis E, Storelli S, Lang NP. Use of short implants (6 mm) in a single-tooth replacement: a 5-year follow-up prospective randomized controlled multicenter clinical study. Clin Oral Implants Res. 2016;27(4):458-64. doi: 10.1111/clr.12564

66. Guida L, Bressan E, Cecoro G, Volpe AD, Fabbro MD, Annunziata M. Short versus Longer Implants in Sites without the Need for Bone Augmentation: A Systematic Review and Meta-Analysis of Randomized Controlled Trials. Materials. 2022;15(9). doi: 10.3390/ma15093138

67. Felice P, Cannizzaro G, Barausse C, Pistilli R, Esposito M. Short implants versus longer implants in vertically augmented posterior mandibles: a randomised controlled trial with 5-year after loading follow-up. Eur J Oral Implantol. 2014;7(4):359-69. https://europepmc.org/article/med/25422824

68. Gastaldi G, Felice P, Pistilli V, Barausse C, Ippolito DR, Esposito M. Posterior atrophic jaws rehabilitated with prostheses supported by 5 × 5 mm implants with a nanostructured calcium-incorporated titanium surface or by longer implants in augmented bone. 3-year results from a randomised controlled trial. Eur J Oral Implantol. 2018;11(1):49-61. https://europepmc.org/article/med/29557400

69. Gastaldi G, Felice P, Pistilli R, Barausse C, Trullenque-Eriksson A, Esposito M. Short implants as an alternative to crestal sinus lift: a 3-year multicentre randomised controlled trial. Eur J Oral Implantol. 2017;10(4):391-400. https://research.ebsco.com/linkprocessor/plink?id=32d96242-8e2c-3a5c-bc8f-938365ebb866

70. Guljé F, Abrahamsson I, Chen S, Stanford C, Zadeh H, Palmer R. Implants of 6 mm vs. 11 mm lengths in the posterior maxilla and mandible: a 1-year multicenter randomized controlled trial. Clin Oral Implants Res. 2013;24(12):1325-31. doi: 10.1111/clr.12001

71. Guljé FL, Raghoebar GM, Vissink A, Meijer HJA. Single crowns in the resorbed posterior maxilla supported by either 11-mm implants combined with sinus floor elevation or 6-mm implants:A 5-year randomised controlled trial. Int J Oral Implantol Berl Ger. 2019;12(3):315-26. doi: https://europepmc.org/article/med/31535100

72. Schincaglia GP, Thoma DS, Haas R, Tutak M, Garcia A, Taylor TD, et al. Randomized controlled multicenter study comparing short dental implants (6 mm) versus longer dental implants (11-15 mm) in combination with sinus floor elevation procedures. Part 2: clinical and radiographic outcomes at 1 year of loading. J Clin Periodontol. 2015;42(11):1042-51. doi: 10.1111/jcpe.13025

73. Esposito M, Barausse C, Pistilli R, Sammartino G, Grandi G, Felice P. Short implants versus bone augmentation for placing longer implants in atrophic maxillae: One-year post-loading results of a pilot randomised controlled trial. Eur J Oral Implantol. 2015;8(3):257-68. doi: 10.1111/jcpe.12465

74. Liang L, Wu X, Yan Q, Shi B. Are short implants (≤8.5 mm) reliable in the rehabilitation of completely edentulous patients: A systematic review and meta-analysis. J Prosthet Dent. mayo de 2024;131(5):826-32. doi: 10.1016/j.prosdent.2022.02.015

75. Liang L, Wu X, Yan Q, Shi B. Are short implants (≤8.5 mm) reliable in the rehabilitation of completely edentulous patients: A systematic review and meta-analysis. J Prosthet Dent. 2022;S0022-3913(22):140-8. doi: 10.1016/j.prosdent.2022.02.015

76. Thoma DS, Haas R, Tutak M, Garcia A, Schincaglia GP, Hämmerle CHF. Randomized controlled multicentre study comparing short dental implants (6 mm) versus longer dental implants (11-15 mm) in combination with sinus floor elevation procedures. Part 1: demographics and patient-reported outcomes at 1 year of loading. J Clin Periodontol. 2015;42(1):72-80. doi: 10.1111/jcpe.12323

77. Cannizzaro G, Felice P, Minciarelli AF, Leone M, Viola P, Esposito M. Early implant loading in the atrophic posterior maxilla: 1-stage lateral versus crestal sinus lift and 8 mm hydroxyapatite-coated implants. A 5-year randomised controlled trial. Eur J Oral Implantol. 2013;6(1):13-25. doi: https://europepmc.org/article/med/23513199

78. Tang C, Du Q, Luo J, Peng L. Simultaneous placement of short implants (≤ 8 mm) versus standard length implants (≥ 10 mm) after sinus floor elevation in atrophic posterior maxillae: a systematic review and meta-analysis. Int J Implant Dent. 2022;8(1):45. doi: 10.1186/s40729-022-00443-1

79. Shi JY, Li Y, Qiao SC, Gu YX, Xiong YY, Lai HC. Short versus longer implants with osteotome sinus floor elevation for moderately atrophic posterior maxillae: A 1-year randomized clinical trial. J Clin Periodontol. 2019;46(8):855-62. doi: 10.1111/jcpe.13147

80. Felice P, Checchi L, Barausse C, Pistilli R, Sammartino G, Masi I, et al. Posterior jaws rehabilitated with partial prostheses supported by 4.0 x 4.0 mm or by longer implants: One-year post-loading results from a multicenter randomised controlled trial. Eur J Oral Implantol. 2016;9(1):35-45. https://europepmc.org/article/med/27022635

81. Felice P, Barausse C, Pistilli V, Piattelli M, Ippolito DR, Esposito M. Posterior atrophic jaws rehabilitated with prostheses supported by 6 mm long × 4 mm wide implants or by longer implants in augmented bone. 3-year post-loading results from a randomised controlled trial. Eur J Oral Implantol. 2018;11(2):175-87. https://research.ebsco.com/linkprocessor/plink?id=5f8f09c9-9cb2-3b98-bd8b-2905c9c39d28

82. Rokn AR, Monzavi A, Panjnoush M, Hashemi HM, Kharazifard MJ, Bitaraf T. Comparing 4-mm dental implants to longer implants placed in augmented bones in the atrophic posterior mandibles: One-year results of a randomized controlled trial. Clin Implant Dent Relat Res. 2018;20(6):997-1002. doi: 10.1111/cid.12672

83. Esposito M, Cannizarro G, Soardi E, Pellegrino G, Pistilli R, Felice P. A 3-year post-loading report of a randomised controlled trial on the rehabilitation of posterior atrophic mandibles: short implants or longer implants in vertically augmented bone? Eur J Oral Implantol. 2011;4(4):301-11. https://research.ebsco.com/linkprocessor/plink?id=2811daf7-db09-343d-993b-f291332442f7

84. Guida L, Annunziata M, Esposito U, Sirignano M, Torrisi P, Cecchinato D. 6-mm-short and 11-mm-long implants compared in the full-arch rehabilitation of the edentulous mandible: A 3-year multicenter randomized controlled trial. Clin Oral Implants Res. 2020;31(1):64-73. doi: 10.1111/clr.13547

85. Rosa A, Pujia AM, Arcuri C. Complete Full Arch Supported by Short Implant (<8 mm) in Edentulous Jaw: A Systematic Review. Appl Sci. 2023;13(12):7162. doi: 10.3390/app13127162

86. Naenni N, Sahrmann P, Schmidlin PR, Attin T, Wiedemeier DB, Sapata V, et al. Five-Year Survival of Short Single-Tooth Implants (6 mm): A Randomized Controlled Clinical Trial. J Dent Res. julio de 2018;97(8):887-92. doi: 10.1177/0022034518758036

87. Felice P, Pellegrino G, Checchi L, Pistilli R, Esposito M. Vertical augmentation with interpositional blocks of anorganic bovine bone vs. 7-mm-long implants in posterior mandibles: 1-year results of a randomized clinical trial. Clin Oral Implants Res. 2010;21(12):1394-403. doi: 10.1111/j.1600-0501.2010.01966.x

88. Zadeh HH, Guljé F, Palmer PJ, Abrahamsson I, Chen S, Mahallati R, et al. Marginal bone level and survival of short and standard-length implants after 3 years: An Open Multi-Center Randomized Controlled Clinical Trial. Clin Oral Implants Res. 2018;29(8):894-906. doi: 10.1111/clr.13341

89. Romeo E, Storelli S, Casano G, Scanferla M, Botticelli D. Six-mm versus 10-mm long implants in the rehabilitation of posterior edentulous jaws: a 5-year follow-up of a randomised controlled trial. Eur J Oral Implantol. 2014;7(4):371-81. https://research.ebsco.com/linkprocessor/plink?id=29895d3e-2425-3fd2-a516-a5392c0503a2

90. Cannizzaro G, Felice P, Buti J, Leone M, Ferri V, Esposito M. Immediate loading of fixed cross-arch prostheses supported by flapless-placed supershort or long implants: 1-year results from a randomised controlled trial. Eur J Oral Implantol. 2015;8(1):27-36. https://research.ebsco.com/linkprocessor/plink?id=5fbd08bd-3200-3122-aeef-75ee812afb2d

91. Wu H, Shi Q, Huang Y, Chang P, Huo N, Jiang Y, et al. Failure Risk of Short Dental Implants Under Immediate Loading: A Meta-Analysis. J Prosthodont. 2021;30(7):569-80. doi: 10.1111/jopr.13376

92. Cannizzaro G, Felice P, Ippolito DR, Velasco-Ortega E, Esposito M. Immediate loading of fixed cross-arch prostheses supported by flapless-placed 5 mm or 11.5 mm long implants: 5-year results from a randomised controlled trial. Eur J Oral Implantol. 2018;11(3):295-306. https://research.ebsco.com/linkprocessor/plink?id=ff892927-a89c-38d0-9eda-0b9699393ad5

93. Weerapong K, Sirimongkolwattana S, Sastraruji T, Khongkhunthian P. Comparative study of immediate loading on short dental implants and conventional dental implants in the posterior mandible: A randomized clinical trial. Int J Oral Maxillofac Implants. 2019;34(1):141-9. doi: 10.11607/jomi.6732

94. Yu H, Wang X, Qiu L. Outcomes of 6.5-mm Hydrophilic Implants and Long Implants Placed with Lateral Sinus Floor Elevation in the Atrophic Posterior Maxilla: A Prospective, Randomized Controlled Clinical Comparison. Clin Implant Dent Relat Res. 2017;19(1):111-22. doi: 10.1111/cid.12439

95. Shah SN, Chung J, Kim DM, Machtei EE. Can extra-short dental implants serve as alternatives to bone augmentation? A preliminary longitudinal randomized controlled clinical trial. Quintessence Int. 2018;49(8):635-43. doi: 10.3290/j.qi.a40763

96. Bernardi S, Gatto R, Severino M, Botticelli G, Caruso S, Rastelli C, et al. Short Versus Longer Implants in Mandibular Alveolar Ridge Augmented Using Osteogenic Distraction: One-Year Follow-up of a Randomized Split-Mouth Trial. J Oral Implantol. junio de 2018;44(3):184-91. doi: 10.1563/aaid-joi-D-16-00216

97. Storelli S, Abbà A, Scanferla M, Botticelli D, Romeo E. 6 mm vs 10 mm-long implants in the rehabilitation of posterior jaws: A 10-year follow-up of a randomised controlled trial. Eur J Oral Implantol. 2018;11(3):283-92. https://research.ebsco.com/linkprocessor/plink?id=dea608fd-322e-33c0-8ee4-f89ae4b33ee5

98. Felice P, Barausse C, Pistilli R, Ippolito DR, Esposito M. Short implants versus longer implants in vertically augmented posterior mandibles: result at 8 years after loading from a randomised controlled trial. Eur J Oral Implantol. 2018;11(4):385-95. https://research.ebsco.com/linkprocessor/plink?id=c62cf5e2-19ee-34d3-b412-34a999d36026

99. Nielsen HB, Søren S, Bruun NH, Starch-Jensen T. Single-crown restorations supported by short implants (6 mm) compared with standard-length implants (13 mm) in conjunction with maxillary sinus floor augmentation: a randomized, controlled clinical trial. Int J Implant Dent. 2021;7(1):66. doi: 10.1186/s40729-021-00348-5

100. Shi JY, Lai YR, Qian SJ, Qiao SC, Tonetti MS, Lai HC. Clinical, radiographic and economic evaluation of short-6-mm implants and longer implants combined with osteotome sinus floor elevation in moderately atrophic maxillae: A 3-year randomized clinical trial. J Clin Periodontol. 2021;48(5):695-704. doi: 10.1111/jcpe.13444

101. Sahrmann P, Naenni N, Jung RE, Held U, Truninger T, Hämmerle CHF, et al. Success of 6-mm Implants with Single-Tooth Restorations: A 3-year Randomized Controlled Clinical Trial. J Dent Res. 2016;95(6):623-8. doi: 10.1177/0022034516633432

102. Hadzik J, Krawiec M, Kubasiewicz-Ross P, Prylińska-Czyżewska A, Gedrange T, Dominiak M. Short Implants and Conventional Implants in The Residual Maxillary Alveolar Ridge: A 36-Month Follow-Up Observation. Med Sci Monit Int Med J Exp Clin Res. 2018;24:5645-52. doi: 10.12659/MSM.910404

103. Esposito M, Zucchelli G, Barausse C, Pistilli R, Trullenque-Eriksson A, Felice P. Four mm-long versus longer implants in augmented bone in atrophic posterior jaws: 4-month post-loading results from a multicentre randomised controlled trial. Eur J Oral Implantol. 2016;9(4):393-409. https://research.ebsco.com/linkprocessor/plink?id=d4333127-212a-35ce-a4db-5bc3ae0fc917

104. Zhang XM, Shi JY, Gu YX, Qiao SC, Mo JJ, Lai HC. Clinical Investigation and Patient Satisfaction of Short Implants Versus Longer Implants with Osteotome Sinus Floor Elevation in Atrophic Posterior Maxillae: A Pilot Randomized Trial. Clin Implant Dent Relat Res. 2017;19(1):161-6. doi: 10.1111/cid.12435

105. Taschieri S, Lolato A, Testori T, Francetti L, Del Fabbro M. Short dental implants as compared to maxillary sinus augmentation procedure for the rehabilitation of edentulous posterior maxilla: Three-year results of a randomized clinical study. Clin Implant Dent Relat Res. 2018;20(1):9-20. doi: 10.1111/cid.12563

106. Hadzik J, Kubasiewicz-Ross P, Nawrot-Hadzik I, Gedrange T, Pitułaj A, Dominiak M. Short (6 mm) and Regular Dental Implants in the Posterior Maxilla-7-Years Follow-up Study. J Clin Med. 1 de marzo de 2021;10(5):940. doi: 10.3390/jcm10050940

107. Rossi F, Tuci L, Ferraioli L, Ricci E, Suerica A, Botticelli D, et al. Two-Year Follow-Up of 4-mm-Long Implants Used as Distal Support of Full-Arch FDPs Compared to 10-mm Implants Installed after Sinus Floor Elevation. A Randomized Clinical Trial. Int J Environ Res Public Health. 2021;18(7):3846. doi: 10.3390/ijerph18073846

108. Calvo-Guirado JL, López Torres JA, Dard M, Javed F, Pérez-Albacete Martínez C, Maté Sánchez de Val JE. Evaluation of extrashort 4-mm implants in mandibular edentulous patients with reduced bone height in comparison with standard implants: a 12-month results. Clin Oral Implants Res. 2016;27(7):867-74. doi: 10.1111/clr.12704

109. Guljé FL, Meijer HJA, Abrahamsson I, Barwacz CA, Chen S, Palmer PJ, et al. Comparison of 6-mm and 11-mm dental implants in the posterior region supporting fixed dental prostheses: 5-year results of an open multicenter randomized controlled trial. Clin Oral Implants Res. 2021;32(1):15-22. doi: 10.1111/clr.13674

110. Felice P, Checchi V, Pistilli R, Scarano A, Pellegrino G, Esposito M. Bone augmentation versus 5-mm dental implants in posterior atrophic jaws. Four-month post-loading results from a randomised controlled clinical trial. Eur J Oral Implant. 2009;2(4):267-81. https://europepmc.org/article/med/20467603

111. Stellingsma K, Raghoebar GM, Visser A, Vissink A, Meijer HJA. The extremely resorbed mandible, 10-year results of a randomized controlled trial on 3 treatment strategies. Clin Oral Implants Res. 2014;25(8):926-32. doi: 10.1111/clr.12184

112. Queiroz TP, Aguiar SC, Margonar R, de Souza Faloni AP, Gruber R, Luvizuto ER. Clinical study on survival rate of short implants placed in the posterior mandibular region: resonance frequency analysis. Clin Oral Implants Res. 2015;26(9):1036-42. doi: 10.1111/clr.12394

113. Rossi F, Ricci E, Marchetti C, Lang NP, Botticelli D. Early loading of single crowns supported by 6-mm-long implants with a moderately rough surface: a prospective 2-year follow-up cohort study. Clin Oral Implants Res. 2010;21(9):937-43. doi: 10.1111/j.1600-0501.2010.01942.x

114. Mezzomo LA, Miller R, Triches D, Alonso F, Shinkai RSA. Meta-analysis of single crowns supported by short (<10 mm) implants in the posterior region. J Clin Periodontol. 2014;41(2):191-213. doi: 10.1111/jcpe.12180

115. Monje A, Chan HL, Fu JH, Suarez F, Galindo-Moreno P, Wang HL. Are short dental implants (<10 mm) effective? A meta-analysis on prospective clinical trials. J Periodontol. 2013;84(7):895-904. doi: 10.1902/jop.2012.120328

116. Romeo E, Ghisolfi M, Rozza R, Chiapasco M, Lops D. Short (8-mm) dental implants in the rehabilitation of partial and complete edentulism: a 3- to 14-year longitudinal study. Int J Prosthodont. 2006;19(6):586-92. https://research.ebsco.com/linkprocessor/plink?id=b0455980-3e40-3fdd-8852-f293c12e3fb9

117. Kotsovilis S, Fourmousis I, Karoussis IK, Bamia C. A systematic review and meta-analysis on the effect of implant length on the survival of rough-surface dental implants. J Periodontol. 2009;80(11):1700-18. doi: 10.1902/jop.2009.090107

118. Ferrigno N, Laureti M, Fanali S. Dental implants placement in conjunction with osteotome sinus floor elevation: a 12-year life-table analysis from a prospective study on 588 ITI implants. Clin Oral Implants Res. 2006;17(2):194-205. doi: 10.1111/j.1600-0501.2005.01192.x

119. Nedir R, Bischof M, Briaux JM, Beyer S, Szmukler-Moncler S, Bernard JP. A 7-year life table analysis from a prospective study on ITI implants with special emphasis on the use of short implants. Results from a private practice. Clin Oral Implants Res. 2004;15(2):150-7. doi: 10.1111/j.1600-0501.2004.00978.x

120. Deporter DA, Todescan R, Watson PA, Pharoah M, Pilliar RM, Tomlinson G. A prospective human clinical trial of Endopore dental implants in restoring the partially edentulous maxilla using fixed prostheses. Int J Oral Maxillofac Implants. 2001;16(4):527-36. https://pubmed.ncbi.nlm.nih.gov/11516000/

121. Guljé FL, Raghoebar GM, Gareb B, Vissink A, Meijer HJA. Single crowns in the posterior maxilla supported by either 11-mm long implants with sinus floor augmentation or by 6-mm long implants: A 10-year randomized controlled trial. Clin Oral Implants Res. enero de 2024;35(1):89-100. doi: 10.1111/clr.14200

122. Barausse C, Felice P, Pistilli R, Buti J, Esposito M. Posterior jaw rehabilitation using partial prostheses supported by implants 4.0×4.0 mm or longer: three-year postloading results of a multicentrerandomised controlled trial. Clin Trials Dent. 2019;1(Issue 1):25-36. doi: 10.36130/ctd.01.2019.03

123. Cannizzaro G, Felice P, Leone M, Viola P, Esposito M. Early loading of implants in the atrophic posterior maxilla: lateral sinus lift with autogenous bone and Bio-Oss versus crestal mini sinus lift and 8-mm hydroxyapatite-coated implants. A randomised controlled clinical trial. Eur J Oral Implantol. 2009;2(1):25-38. https://research.ebsco.com/linkprocessor/plink?id=7b0cc528-bf3c-398b-9798-38d906584b58

124. Esposito M, Barausse C, Pistilli R, Checchi V, Diazzi M, Gatto M, et al. Posterior jaws rehabilitated with partial prostheses supported by 4.0 x 4.0 mm or by longer implants: Four-month post-loading data from a randomised controlled trial. Eur J Oral Implantol. 2015;8(3):221-30. https://research.ebsco.com/linkprocessor/plink?id=24c42f2c-ce66-3c81-a2c5-7a77ae9e25cf

125. Felice P, Marchetti C, Iezzi G, Piattelli A, Worthington H, Pellegrino G, et al. Vertical ridge augmentation of the atrophic posterior mandible with interpositional bloc grafts: bone from the iliac crest vs. bovine anorganic bone. Clinical and histological results up to one year after loading from a randomized-controlled clinical trial. Clin Oral Implants Res. 2009;20(12):1386-93. doi: 10.1111/j.1600-0501.2009.01765.x

126. Felice P, Pistilli R, Lizio G, Pellegrino G, Nisii A, Marchetti C. Inlay versus onlay iliac bone grafting in atrophic posterior mandible: a prospective controlled clinical trial for the comparison of two techniques. Clin Implant Dent Relat Res. 2009;11(Suppl 1):e69-82. doi: 10.1111/j.1708-8208.2009.00212.x

127. Bianchi A, Felice P, Lizio G, Marchetti C. Alveolar distraction osteogenesis versus inlay bone grafting in posterior mandibular atrophy: a prospective study. Oral Surg Oral Med Oral Pathol Oral Radiol Endod. 2008;105(3):282-92. doi: 10.1016/j.tripleo.2007.07.009

128. Chiapasco M, Zaniboni M, Rimondini L. Autogenous onlay bone grafts vs. alveolar distraction osteogenesis for the correction of vertically deficient edentulous ridges: a 2-4-year prospective study on humans. Clin Oral Implants Res. 2007;18(4):432-40. doi: 10.1111/j.1600-0501.2007.01351.x

129. Esposito M, Cannizzaro G, Soardi E, Pistilli R, Piattelli M, Corvino V, et al. Posterior atrophic jaws rehabilitated with prostheses supported by 6 mm-long, 4 mm-wide implants or by longer implants in augmented bone. Preliminary results from a pilot randomised controlled trial. Eur J Oral Implantol. 2012;5(1):19-33. https://research.ebsco.com/linkprocessor/plink?id=79dc4de3-57b7-356b-a943-365c619beb82

130. Cannizzaro G, Felice P, Leone M, Ferri V, Viola P, Esposito M. Immediate versus early loading of 6.5 mm-long flapless-placed single implants: a 4-year after loading report of a split-mouth randomised controlled trial. Eur J Oral Implantol. 2012;5(2):111-21. https://research.ebsco.com/linkprocessor/plink?id=c9eaee28-32f6-3b04-8a4f-65f10264016c

131. Perelli M, Abundo R, Corrente G, Saccone C. Short (5 and 7 mm long) porous implant in the posterior atrophic mandible: a 5-year report of a prospective study. Eur J Oral Implantol. 2011;4(4):363-8. https://research.ebsco.com/linkprocessor/plink?id=6bb22035-d28e-323f-a66d-6648126353e1

132. Telleman G, Meijer HJA, Vissink A, Raghoebar GM. Short implants with a nanometer-sized CaP surface provided with either a platform-switched or platform-matched abutment connection in the posterior region: a randomized clinical trial. Clin Oral Implants Res. 2013;24(12):1316-24. doi: 10.1111/clr.12000

133. Romeo E, Chiapasco M, Ghisolfi M, Vogel G. Long-term clinical effectiveness of oral implants in the treatment of partial edentulism. Seven-year life table analysis of a prospective study with ITI dental implants system used for single-tooth restorations. Clin Oral Implants Res. abril de 2002;13(2):133-43. doi: 10.1034/j.1600-0501.2002.130203.x

134. Brocard D, Barthet P, Baysse E, Duffort JF, Eller P, Justumus P, et al. A multicenter report on 1,022 consecutively placed ITI implants: a 7-year longitudinal study. Int J Oral Maxillofac Implants. 2000;15(5):691-700. https://research.ebsco.com/linkprocessor/plink?id=69c21b5d-f752-3838-a29c-8941bbd73a37

135. Strietzel FP, Reichart PA. Oral rehabilitation using Camlog screw-cylinder implants with a particle-blasted and acid-etched microstructured surface. Results from a prospective study with special consideration of short implants. Clin Oral Implants Res. 2007;18(5):591-600. doi: 10.1111/j.1600-0501.2007.01375.x

136. Amato F, Polara G, Spedicato GA. Immediate Loading of Fixed Partial Dental Prostheses on Extra-Short and Short Implants in Patients with Severe Atrophy of the Posterior Maxilla or Mandible: An Up-to-4-year Clinical Study. Int J Oral Maxillofac Implants. 2020;35(3):607-15. doi: 10.11607/jomi.7943

137. Anitua E, Flores C, Flores J, Alkhraisat MH. Clinical Effectiveness of 6.5-mm-Long Implants to Support Two-Implant Fixed Prostheses in Premolar-Molar Region: The Influence of Immediate Loading and the Length of Splinting Implant. J Prosthodont. 2019;28(2):e688-93. doi: 10.1111/jopr.12761

138. Anitua E, Flores J, Flores C, Alkhraisat MH. Long-term Outcomes of Immediate Loading of Short Implants: A Controlled Retrospective Cohort Study. Int J Oral Maxillofac Implants. 2016;31(6):1360-6. doi: 10.11607/jomi.5330

139. Arlin ML. Short dental implants as a treatment option: results from an observational study in a single private practice. Int J Oral Maxillofac Implants. 2006;21(5):769-76. https://research.ebsco.com/linkprocessor/plink?id=93f27abc-013d-3e7d-aa77-321b9a66f736

140. Degidi M, Piattelli A, Carinci F. Parallel screw cylinder implants: comparative analysis between immediate loading and two-stage healing of 1,005 dental implants with a 2-year follow up. Clin Implant Dent Relat Res. 2006;8(3):151-60. doi: 10.1111/j.1708-8208.2006.00007.x

141. Cochran DL, Buser D, ten Bruggenkate CM, Weingart D, Taylor TM, Bernard JP, et al. The use of reduced healing times on ITI implants with a sandblasted and acid-etched (SLA) surface: early results from clinical trials on ITI SLA implants. Clin Oral Implants Res. 2002;13(2):144-53. doi: 10.1034/j.1600-0501.2002.130204.x

142. Hallman M. A prospective study of treatment of severely resorbed maxillae with narrow nonsubmerged implants: results after 1 year of loading. Int J Oral Maxillofac Implants. 2001;16(5):731-6. https://research.ebsco.com/linkprocessor/plink?id=907208f3-0e8a-382d-8df0-56d82f7cc73d

143. Malmstrom H, Gupta B, Ghanem A, Cacciato R, Ren Y, Romanos GE. Success rate of short dental implants supporting single crowns and fixed bridges. Clin Oral Implants Res. 2016;27(9):1093-8. doi: 10.1111/clr.12693

144. McGlumphy EA, Peterson LJ, Larsen PE, Jeffcoat MK. Prospective study of 429 hydroxyapatite-coated cylindric omniloc implants placed in 121 patients. Int J Oral Maxillofac Implants. 2003;18(1):82-92. https://research.ebsco.com/linkprocessor/plink?id=998578dc-36f8-3eea-ab9f-1828582e7f4c

145. Polizzi G, Rangert B, Lekholm U, Gualini F, Lindström H. Brånemark System Wide Platform implants for single molar replacement: clinical evaluation of prospective and retrospective materials. Clin Implant Dent Relat Res. 2000;2(2):61-9. doi: 10.1111/j.1708-8208.2000.tb00107.x

146. Roccuzzo M, Bunino M, Prioglio F, Bianchi SD. Early loading of sandblasted and acid-etched (SLA) implants: a prospective split-mouth comparative study. Clin Oral Implants Res. 2001;12(6):572-8. doi: 10.1034/j.1600-0501.2001.120604.x

147. Roccuzzo M, Wilson T. A prospective study evaluating a protocol for 6 weeks’ loading of SLA implants in the posterior maxilla: one year results. Clin Oral Implants Res. 2002;13(5):502-7. doi: 10.1034/j.1600-0501.2002.130509.x

148. Tawil G, Younan R. Clinical evaluation of short, machined-surface implants followed for 12 to 92 months. Int J Oral Maxillofac Implants. 2003;18(6):894-901. https://research.ebsco.com/linkprocessor/plink?id=321f6ab7-8e6a-38ec-a4d7-fc463ddb4a31

149. Testori T, Del Fabbro M, Feldman S, Vincenzi G, Sullivan D, Rossi R, et al. A multicenter prospective evaluation of 2-months loaded Osseotite implants placed in the posterior jaws: 3-year follow-up results. Clin Oral Implants Res. 2002;13(2):154-61. https://research.ebsco.com/linkprocessor/plink?id=5f2f784a-4f52-3b1b-8699-c85ae81dd185

150. Barausse C, Pistilli R, Canullo L, Bonifazi L, Ferri A, Felice P. A 5-year randomized controlled clinical trial comparing 4-mm ultrashort to longer implants placed in regenerated bone in the posterior atrophic jaw. Clin Implant Dent Relat Res. febrero de 2022;24(1):4-12. doi: 10.1111/cid.13061

151. Magdy M, Abdelkader MA, Alloush S, Fawzy El-Sayed KM, Nawwar AA, Shoeib M, et al. Ultra-short versus standard-length dental implants in conjunction with osteotome-mediated sinus floor elevation: A randomized controlled clinical trial. Clin Implant Dent Relat Res. agosto de 2021;23(4):520-9. doi: 10.1111/cid.12995
