# Supplementary material for: Clinical performance of short implants vs. standard implants in edentulous patients. An umbrella review
Source: Front Oral Health. 2025 Sep 18;6:1670095. doi: 10.3389/froh.2025.1670095 (PMC12488567; doi:10.3389/froh.2025.1670095)
Supplement: Supplementary Material S6 — Implant lengths compared across included systematic reviews—a table summarizing the number of studies, main authors, implant lengths compared, and clinical outcomes. [file Table6.docx]

Supplementary Material 3. Characteristics of included studies

| **Authors** | **Year** | **Study design** | **Included study design** | **Number of studies (qualitative/quantitative)** | **Type of dental implants** | **Region/Condition** | **Outcomes** | | **Conclusions** |
| --- | --- | --- | --- | --- | --- | --- | --- | --- | --- |
| Alemán et al. (1) | 2025 | SR with MA | RCTs | 7/7 | Short implants (≤6 mm) | Partial atrophic edentulous maxilla / Sinus floor elevation | Survival rate | RR = 0.9 (0.63 - 1.3) | No definitive conclusions can be drawn regarding the success or survival of short versus long implants. |
|  |  |  |  |  |  |  | Marginal bone loss | SMD = -0.41 (-0.72 - -0.09) |  |
|  |  |  |  |  | Standard implants (>6 mm) |  | Complications rate | RR = 0.88 (0.64 - 1.21) |  |
| Abayov et al. (2) | 2024 | SR with MA | RCTs | 9/9 | Short implants (≤8 mm) | Partial or total maxillary and/or mandibular edentulism / Bone augmentation | Survival rate | 93.91% - 91.83% | Short implants showed better clinical results in terms of survival rate, marginal bone loss, and complications. |
|  |  |  |  |  | Standard implants (>8 mm) |  | Marginal bone loss | SMD = -0.51 (-0.93 - -0.1) |  |
| Zhang et al. (3) | 2024 | SR with MA | RCTs and RS | 17/17 | Short implants (≤8 mm) | Partial atrophic edentulous maxilla / Sinus floor elevation | Implants failure | OR = 1.26 (0.53 - 3.0) | In patients with limited vertical bone height in the posterior maxilla who need dental implants, the use of short implants with sinus lifting and short implants without sinus lifting can yield similar results to long implants with sinus floor elevation. |
|  |  |  |  |  |  |  | Marginal bone loss | MD = -0.17 (-0.29 - -0.05) |  |
|  |  |  |  |  | Standard implants (>8 mm) |  | Biological complications | OR = 0.47 (0.19 - 1.18) |  |
|  |  |  |  |  |  |  | Prosthetic complications | OR = 0.94 (0.45 - 1.94) |  |
| Emfietzoglou et al. (4) | 2024 | SR with MA | RCTs | 16/9 | Short implants (≤6 mm) | Maxillary or mandibular partial edentulism / Bone augmentation | Survival rate | RD = 0.05 (0.03 - 0.07) | Short implants represent a promising alternative treatment option for the rehabilitation of posterior jaws to avoid additional bone augmentation procedures. |
|  |  |  |  |  |  |  | Marginal bone loss | SMD = -0.09 (-0.18 - 0.01) |  |
|  |  |  |  |  | Standard implants (>6 mm) |  | Prosthetic complications | RD = 0.01 (-0.04 - 0.06) |  |
| Liang et al. (5) | 2024 | SR with MA | RCTs | 4/4 | Short implants (≤8.5 mm) | Completely edentulous maxilla and/or mandible / Bone augmentation | Survival rate | OR = 1.42 (0.21 - 9.63) | For completely edentulous jaws, short implants provide a reliable treatment alternative, as survival and MBL were not different when compared with those of long implants. |
|  |  |  |  |  |  |  |  | RR = 1.01 (0.97 - 1.05) |  |
|  |  |  |  |  | Standard implants (≥10 mm) |  | Marginal bone loss | MD = -0.19 (-0.39 - 0.02) |  |
| Kermanshah et al. (6) | 2023 | SR with MA | RCTs | 24/24 | Short implants (≤8 mm) | Posterior jaw / Bone augmentation | Implants failure | RD = 0.01 (-0.002 - 0.02) | Short implants and standard implants had comparable overall outcomes, but short implants had less marginal bone loss and lower biological complications. |
|  |  |  |  |  |  |  | Marginal bone loss | SMD = -0.4 (-0.62 - -0.18) |  |
|  |  |  |  |  | Standard implants (>8 mm) |  | Biological complications | RD = -0.07 (-0.11 - -0.04) |  |
|  |  |  |  |  |  |  | Prosthetic complications | RD = 0.0 (-0.01 - 0.01) |  |
| Mester et al. (7) | 2023 | SR with MA | RCTs | 5/5 | Short implants (≤ 7 mm) | Atrophic posterior maxilla / Sinus floor elevation | Survival rate | RR = 0.97 (0.94 - 1) | Short implants might be used as an alternative to standard implants and sinus floor elevation. |
|  |  |  |  |  |  |  | Marginal bone loss | MD = -0.29 (-0.49 - -0.09) |  |
|  |  |  |  |  | Standard implants (≥ 8 mm) |  | Biological complications | RR = 0.46 (0.23 - 0.91) |  |
|  |  |  |  |  |  |  | Prosthetic complications | RR = 1.51 (0.64 - 3.55) |  |
| Rosa et al. (8) | 2023 | SR | RCTs | 3/0 | Short implants (≤ 8 mm) | Fully edentulous mandible with severe posterior atrophy / NR | Survival rate | 94.2% - 97.4% | Full-arch fixed restorations supported by short implants are a viable option for the treatment of mandibular atrophy. |
|  |  |  |  |  |  |  | Marginal bone loss | 0.12 mm |  |
|  |  |  |  |  | Standard implants (> 8 mm) |  | Biological complications | 0% - 11.1% |  |
|  |  |  |  |  |  |  | Prosthetic complications | 31.8% |  |
| Grunau et al. (9) | 2023 | SR with MA | RCTs | 21/11 | Short implants (≤ 6 mm) | Atrophic posterior maxilla / Sinus floor elevation | Survival rate | RR = 3.28 (0.94 - 11.5) | Short implants versus lateral sinus floor augmentation combined with regular implants demonstrated comparable results within the first 5 years. |
|  |  |  |  |  |  |  | Marginal bone loss | MD = -0.25 (-0.46 - -0.03) |  |
|  |  |  |  |  | Standard implants (≥ 10 mm) |  | Perioperative complications | RR = 0.33 (0.09 - 1.16) |  |
| Zhang et al. (10) | 2023 | SR with MA | RCTs and CCTs | 11/11 | Extra short implants (4 mm) | Posterior maxilla and posterior mandible / Bone augmentation | Survival rate | OR = 1.23 (0.66 - 2.27) | The application of extra short implants when the alveolar crest height of the posterior teeth is less than 5 mm can achieve clinical results comparable to standard implants, and has the advantages of low technical sensitivity and few postoperative biological complications. |
|  |  |  |  |  |  |  | Marginal bone loss | MD = -0.16 (-0.25 - -0.07) |  |
|  |  |  |  |  | Standard implants (≥ 8 mm) |  | Biological complications | RR = 0.34 (0.19 - 0.62) |  |
|  |  |  |  |  |  |  | Prosthetic complications | RR = 2.89 (1.05 - 7.92) |  |
| Liang et al. (11) | 2022 | SR with MA | RCTs | 4/4 | Short implants (≤ 8.5 mm) | Completely edentulous maxilla and/or mandible / Bone augmentation | Survival rate | OR = 1.42 (0.21 - 9.63) | Short implants can be used in completely edentulous jaws with implant survival and marginal bone loss similar to standard implants. |
|  |  |  |  |  | Standard implants (≥ 10 mm) |  | Marginal bone loss | MD = -0.19 (-0.39 - 0.02) |  |
| Wang et al. (12) | 2022 | SR with MA | RCTs | 5/5 | Short implants (< 8 mm) | Atrophic posterior maxilla / Sinus floor elevation | Implant failure | RR = 3.64 (0.91 - 14.53) | Short implants could be an alternative to long implants with an elevated sinus floor for atrophic maxillae in aging populations. |
|  |  |  |  |  |  |  | Marginal bone loss | MD = -0.26 (-0.47 - -0.05) |  |
|  |  |  |  |  | Standard implants (≥ 8 mm) |  | Biological complications | RR = 0.85 (0.46 - 1.58) |  |
|  |  |  |  |  |  |  | Prosthetic complications | RR = 2.61 (1.57 - 4.34) |  |
| Medikeri et al. (13) | 2022 | SR with MA | RCTs | 22/22 | Short implants (≤ 8 mm) | Partial or total maxillary and/or mandibular edentulism / NR | Survival rate | RR = 2.28 (1.46 - 3.57) | Short surface-modified implants are a potential alternative to longer implants in atrophic ridges. |
|  |  |  |  |  | Standard implants (> 8 mm) |  | Marginal bone loss | MD = -0.43 (-0.63 - -0.23) |  |
| Toledano et al. (14) | 2022 | SR with MA | RCTs | 14/14 | Short implants (≤ 6 mm) | Atrophic posterior maxilla / Sinus floor elevation | Survival rate | RR = 1.02 (1.00 - 1.05) | Short dental implants can be used as an alternative to standard-length implants plus sinus elevation in cases of atrophic posterior maxilla. |
|  |  |  |  |  | Standard implants (≥ 8 mm) |  | Marginal bone loss | MD = 0.23 (0.07 - 0.39) |  |
| Guida et al. (15) | 2022 | SR with MA | RCTs | 13/13 | Short implants (≤ 6 mm) | Partial or total maxillary and/or mandibular edentulism / NR | Survival rate | RR = 0.96 (0.87 - 1.07) | Moderate evidence exists suggesting that short implants perform as well as longer ones in the rehabilitation of edentulous sites without the need for bone augmentation. |
|  |  |  |  |  |  |  | Marginal bone loss | MD = -0.47 (-1.18 - 0.24) |  |
|  |  |  |  |  | Standard implants (≥ 8.5 mm) |  | Biological complications | RR = 0.4 (0.09 - 1.70) |  |
|  |  |  |  |  |  |  | Prosthetic complications | RR = 2.66 (1.12 - 6.29) |  |
| Tang et al. (16) | 2022 | SR with MA | RCTs | 3/3 | Short implants (≤ 8 mm) | Atrophic posterior maxilla / Sinus floor elevation | Survival rate | RR = 1.02 (0.96 - 1.08) | The use of short implants combined with osteotome sinus floor elevation could be an alternative to standard implants with sinus floor elevation when the residual bone height of the posterior maxilla is insufficient. |
|  |  |  |  |  |  |  | Marginal bone loss | MD = -0.13 (-0.32 - 0.07) |  |
|  |  |  |  |  | Standard implants (≥ 10 mm) |  | Intra-operative complications | RR = 1.14 (0.46 - 2.83) |  |
|  |  |  |  |  |  |  | Post-operative complications | RR = 1.34 (0.71 - 2.55) |  |
| Terheyden et al. (17) | 2021 | SR with MA | RCTs | 17/15 | Short implants (≤7 mm) | Atrophic posterior jaw / Bone augmentation | Survival rate | RR = 1.53 (0.41 - 5.71) | In the deficient atrophic posterior mandible, short implants and regular implants demonstrate comparable outcomes within the first 5 years. |
|  |  |  |  |  | Standard implants (>7 mm) |  | Marginal bone loss | MD = -0.88 (-1.26 - -0.50) |  |
|  |  |  |  |  |  |  | Perioperative complications | RR = 0.34 (0.19 - 0.60) |  |
| Moraschini et al. (18) | 2021 | SR with MA | RCTs and CCTs | 4/4 | Extra short implants (4 mm) | Mandibular partial edentulism / NR | Survival rate | RR = 0.87 (0.37 - 2.03) | Extra-short dental implants placed in the mandible exhibit satisfactory clinical outcomes concerning implant survival rate and marginal bone loss when compared to longer implants, with a low number of biological and prosthetic complications. |
|  |  |  |  |  |  |  | Marginal bone loss | MD = -0.13 (-0.22 - -0.05) |  |
|  |  |  |  |  | Standard implants (> 8 mm) |  | Biological complications | RR = 0.29 (0.15 - 0.55) |  |
|  |  |  |  |  |  |  | Prosthetic complications | RR = 0.43 (0.13 - 1.43) |  |
| Bitinas et al. (19) | 2021 | SR with MA | RCTs | 21/21 | Short implants (≤ 8 mm) | Partial or complete atrophic edentulous maxilla and/or mandible / Bone augmentation | Implant failure | RD = 0.03 (-0.07 - 0.13) | Short dental implants may be a suitable alternative to standard length dental implants after bone augmentation. |
|  |  |  |  |  | Standard implants (≥ 9 mm) |  | Marginal bone loss | MD = -0.45 (-0.87 - -0.02) |  |
|  |  |  |  |  |  |  | Post-operative complications | RD = -0.39 (-0.92 - 0.14) |  |
| Yu et al. (20) | 2021 | SR with MA | RCTs | 31/31 | Short implants (≤ 6 mm) | Partial or complete atrophic edentulous maxilla and/or mandible / Bone augmentation | Survival rate | RR = 0.97 (0.94 - 1) | The placement of extra-short implants could be an acceptable alternative to longer implants in atrophic posterior arch. |
|  |  |  |  |  |  |  | Marginal bone loss | MD = -0.38 (-0.54 - -0.22) |  |
|  |  |  |  |  | Standard implants (≥ 8 mm) |  | Biological complications | RR = 0.32 (0.24 - 0.42) |  |
|  |  |  |  |  |  |  | Prosthetic complications | RR = 1.09 (0.78 - 1.54) |  |
| Wu et al. (21) | 2021 | SR with MA | RCTs and OSs | 17/17 | Short implants (< 10 mm) | Partial or total maxillary and/or mandibular edentulism / NR | Implant failure | OR = 1.38 (0.67 - 2.84) | There is not enough evidence to show that short dental implants under immediate loading may have higher implant failure risk compared to standard implants under immediate loading and short implants under early or delayed loading. |
|  |  |  |  |  | Standard implants (≥ 10 mm) |  |  |  |  |
| Chaware et al. (22) | 2021 | SR with MA | RCTs | 22/22 | Short implant (< 8.5 mm) | Atrophic posterior maxilla / Sinus floor elevation | Survival rate | RR = 1.09 (0.6 - 2.0) | There was no significance difference in implant survival rate and marginal bone resorption recorded for both the short implant and long implant with sinus graft, in the patients rehabilitated with posterior atrophic maxilla. |
|  |  |  |  |  |  |  | Marginal bone loss | MD = -0.16 (-0.23 - -0.08) |  |
|  |  |  |  |  | Standard implants (≥ 8.5 mm) |  | Biological complications | RR = 0.48 (0.23 - 0.8) |  |
|  |  |  |  |  |  |  | Prosthetic complications | RR = 1.56 (0.85 - 3.15) |  |
| Abdel-Halim et al. (23) | 2021 | SR with MA | RCTs, CCTs, RSs and PSs | 353/353 | Short implants (< 10 mm) | Partial or total maxillary and/or mandibular edentulism / NR | Implant failure | RR = 2.44 (2.18 - 2.73) | Short implants showed a 2.5 times higher risk of failure than long implants. |
|  |  |  |  |  | Standard implants (≥ 10 mm) |  |  |  |  |
| Carosi et al. (24) | 2021 | SR with MA | RCTs | 9/9 | Short implants (≤ 6 mm) | Atrophic posterior maxilla / Sinus floor elevation | Survival rate | RR = 1.24 (0.63 - 2.45) | Based on the evidence of the included studies, short implants reported high survival rates over short to medium follow-up in posterior maxilla, but the long-term success is as yet not demonstrated. |
|  |  |  |  |  | Standard implants (≥ 8 mm) |  |  |  |  |
| Carosi et al. (25) | 2021 | SR | RCTs | 5/0 | Short implants (≤ 6 mm) | Severe mandibular atrophy / Bone augmentation | Survival rate | SI: 92-96.9%; ST: 84.8-100% | Short dental implants are a valid therapeutic choice to rehabilitate severe mandibular atrophy in the medium to long term. |
|  |  |  |  |  | Standard implants (≥ 8 mm) |  | Marginal bone loss | SI: –0.51 to –2.30 mm; ST: –0.77 to –2.64 mm |  |
|  |  |  |  |  |  |  | Prosthetic complications | SI: 0-9.1%; ST: 0-10% |  |
| Xu et al. (26) | 2020 | SR with MA | RCTs | 9/9 | Short implants (< 7 mm) | Partial or total maxillary and/or mandibular edentulism / Bone augmentation | Survival rate | RR = 0.96 (0.94 - 0.99) | The survival rate of short implants in the mandible is similar to that of long implants, and short implants can result in a lower rate of biological complications. |
|  |  |  |  |  |  |  | Marginal bone loss | MD = -0.25 (-0.47 - -0.04) |  |
|  |  |  |  |  | Standard implants (≥ 7 mm) |  | Biological complications | RR = 0.47 (0.33 - 0.67) |  |
|  |  |  |  |  |  |  | Prosthetic complications | RR = 1.74 (1.17 - 2.59) |  |
| Lozano-Carrascal et al. (27) | 2020 | SR with MA | RCTs | 8/8 | Short implants (< 8 mm) | Atrophic posterior maxilla / Sinus floor elevation | Survival rate | RR = 1.08 (0.42 - 2.83) | Prosthetic rehabilitations with short implants in posterior maxilla is a reliable treatment option as an alternative to lateral wall sinus floor augmentation. |
|  |  |  |  |  |  |  | Marginal bone loss | MD = 0.86 (0.75 - 0.98) |  |
|  |  |  |  |  |  |  | Biological complications | RR = 0.46 (0.22 - 0.95) |  |
|  |  |  |  |  | Standard implants (≥ 8 mm) |  | Prosthetic complications | RR = 1.52 (0.91 - 2.54) |  |
|  |  |  |  |  |  |  | Intra-operative complications | RR = 0.51 (0.16 - 1.63) |  |
|  |  |  |  |  |  |  | Post-operative complications | RR = 0.76 (0.33 - 1.74) |  |
| Xu et al. (28) | 2020 | SR with MA | RCTs | 5/5 | Short implants (≤ 6 mm) | Posterior maxilla and/or mandible / Bone augmentation | Survival rate | RR = 0.94 (0.90 - 0.99) | Prostheses placed on short implants do not affect marginal bone loss. However, long-term follow-up comparisons indicated that short implants have a poorer survival rate than standard implants. |
|  |  |  |  |  | Standard implants (>6 mm) |  | Marginal bone loss | MD = 0.00 (-0.10 - 0.11) |  |
| Iezzi et al. (29) | 2020 | SR with MA | RCTs | 25/13 | Short implants (< 7 mm) | Atrophic edentulous maxilla and/or mandible / Bone augmentation | Survival rate | RR = 1.85 (0.61 - 5.62) | Short implants supporting partial fixed rehabilitations represent a valuable alternative to augmentation procedures in the medium term. |
|  |  |  |  |  | Standard implants (≥ 7 mm) |  | Marginal bone loss | MD = -0.47 (-0.69 - -0.24) |  |
|  |  |  |  |  |  |  | complications rate | RR = 0.69 (0.34 - 1.40) |  |
| Vazouras et al. (30) | 2020 | SR with MA | RCTs and Cohorts | 20/20 | Short implants (≤ 6 mm) | Maxillary or mandibular posterior partial edentulism / NR | Implant failure | RD = 0.04 (0.02 - 0.06) | Short implants in function for more than 3 years presented higher failure rates compared to standard implants in function for < 3 years. |
|  |  |  |  |  | Standard implants (≥ 7 mm) |  |  |  |  |
| Mokcheh et al. (31) | 2019 | SR with MA | RCTs, Cohorts and SRs | 18/15 | Short implants (≤ 8 mm) | Atrophic posterior maxilla / Sinus floor elevation | Survival rate | RR = 1.00 (0.89 - 1.12) | Short implants are a reliable alternative compared to standard implants associated with sinus lift. |
|  |  |  |  |  | Standard implants (≥ 10 mm) |  | complications rate | RR = 0.23 (0.09 - 0.62) |  |
| Nielsen et al. (32) | 2019 | SR with MA | RCTs | 3/3 | Short implants (≤ 8 mm) | Atrophic posterior maxilla / Sinus floor elevation | Survival rate | OR = 0.90 (0.15 - 5.44) | Short implants seem to be a suitable alternative to standard length implants in conjunction with maxillary sinus floor augmentation. |
|  |  |  |  |  | Standard implants (> 8 mm) |  | Marginal bone loss | MD = -0.07 (-0.12 - -0.02) |  |
| Altaib et al. (33) | 2019 | SR with MA | RCTs | 13/13 | Short implants (≤ 8 mm) | Atrophic posterior ridge / Bone augmentation | Implant failure | RD = -0.05 (-0.19 - 0.09) | Short dental implants seem to be an effective alternative treatment for the atrophic posterior ridge. |
|  |  |  |  |  | Standard implants (> 8 mm) |  | Marginal bone loss | MD = -0.37 (-1.11 - 0.36) |  |
|  |  |  |  |  |  |  | Post-operative complications | RD = -0.27 (-0.89 - 0.35) |  |
| Ravidà et al. (34) | 2019 | SR with MA | RCTs | 12/12 | Short implants (≤ 6 mm) | Atrophic posterior maxilla / Sinus floor elevation | Survival rate | RR = 0.99 (0.96 - 1.02) | The placement of short implants is a predictable option in treating patients with maxillary atrophy up to a 3-year follow-up. |
|  |  |  |  |  |  |  | Marginal bone loss | MD = 0.23 (-0.38 - -0.07) |  |
|  |  |  |  |  | Standard implants (≥ 10 mm) |  | Biological complications | RR = 0.22 (0.05 - 1.02) |  |
|  |  |  |  |  |  |  | Prosthetic complications | RR = 2.66 (1.12 - 6.34) |  |
| Esposito et al. (35) | 2019 | SR with MA | RCTs | 4/4 | Short implants (≤ 8 mm) | Atrophic posterior mandible / Bone augmentation | Implant failure | RR = 1.00 (0.31 - 3.21) | Five years after loading, prosthetic and implant failures were similar between the two interventions, but complications and peri-implant marginal bone loss were higher and more severe at longer implants placed in vertically augmented mandibles. |
|  |  |  |  |  |  |  | Marginal bone loss | MD = 0.60 (0.36 - 0.83) |  |
|  |  |  |  |  | Standard implants (> 8 mm) |  | Biological complications | RR = 4.72 (2.43 - 9.17) |  |
|  |  |  |  |  |  |  | Prosthetic complications | RR = 1.46 (0.52 - 4.09) |  |
| Amine et al. (36) | 2019 | SR | RCTs | 13/0 | Short implants (≤ 8 mm) | Atrophic posterior mandible / Bone augmentation | Marginal bone loss | SI: - 0.1 to -1.49 mm; ST: - 0.1to -2.34 mm | Short implants present satisfactory results in relation to standard implants. |
|  |  |  |  |  | Standard implants (> 8 mm) |  |  |  |  |
| Chen et al. (37) | 2019 | SR with MA | RCTs | 10/10 | Short implants (≤ 8 mm) | Atrophic posterior mandible / Bone augmentation | Survival rate | RR = 1.01 (0.99 - 1.03) | This systematic review showed no difference between the survival rates and complications of short implants and standard implants. The marginal bone loss changes in short implants are lower than those in long implants. |
|  |  |  |  |  | Standard implants (≥ 10 mm) |  | Marginal bone loss | MD = -0.13 (-0.20 - -0.06) |  |
|  |  |  |  |  |  |  | complications rate | RR = 0.48 (0.20 - 1.17) |  |
| Bitaraf et al. (38) | 2019 | SR with MA | RCTs | 23/23 | Short implants (≤ 8 mm) | Maxillary or mandibular partial edentulism / Bone augmentation | Implant failure | RR = 1.21 (0.52 - 2.83) | Short implants and standard implants showed the comparable outcomes except biological complication preferring short implants. |
|  |  |  |  |  |  |  | Marginal bone loss | MD = -0.42 (-0.58 - -0.25) |  |
|  |  |  |  |  | Standard implants (> 8 mm) |  | Biological complications | RR = 0.25 (0.15 - 0.40) |  |
|  |  |  |  |  |  |  | Prosthetic complications | RR = 0.58 (0.22 - 1.51) |  |
| Ravidà et al. (39) | 2019 | SR with MA | RCTs | 18/18 | Short implants (≤ 6 mm)  Standard implants (≥ 10 mm) | Atrophic posterior maxilla and/or mandible / Bone augmentation | Survival rate | RR = 0.92 (0.86 - 0.99) | Extra-short implants are an equivalent option in the treatment of patients with atrophic posterior arch up to 3 years of follow-up. |
|  |  |  |  |  |  |  | Marginal bone loss | MD = -0.23 (-0.34 - -0.13) |  |
|  |  |  |  |  |  |  | Biological complications | RR = 1.79 (0.25 - 12.78) |  |
|  |  |  |  |  |  |  | Prosthetic complications | RR = 1.49 (0.45 - 4.96) |  |
| Yan et al. (40) | 2019 | SR with MA | RCTs | 7/7 | Short implants (≤ 6 mm) | Atrophic posterior maxilla / Sinus floor elevation | Survival rate | RR = 1.00 (0.97 - 1.04) | For atrophic posterior maxilla, short implants are a promising alternative to sinus floor elevation, with comparable survival rate, less marginal bone loss and post-surgery reactions. |
|  |  |  |  |  |  |  | Marginal bone loss | MD = -0.25 (-0.40 - -0.10) |  |
|  |  |  |  |  | Standard implants (≥ 10 mm) |  | Biological complications | RR = 0.91 (0.14 - 5.79) |  |
|  |  |  |  |  |  |  | Prosthetic complications | RR = 2.66 (0.93 - 7.60) |  |
| Aldawood et al. (41) | 2019 | SR with MA | PS | 5/5 | Short implants (≤ 8 mm) | Total or partial posterior edentulism of the mandible / NR | Survival rate | RR = 1.06 (1.02 - 1.1) | Short implant placement is as effective a treatment modality as conventional implant placement in totally and partially edentulous patients. |
|  |  |  |  |  | Standard implants (> 8 mm) |  | complications rate | RR = 0.83 |  |
| De N Dias et al. (42) | 2019 | SR with MA | RCTs | 4/4 | Short implants (≤ 8 mm) | Posterior atrophic partially edentulous mandible / Bone augmentation | Survival rate | RR = 1.05 (1.00 - 1.09) | In spite of similar survival rates when the residual bone is sufficient for placement of short implants, the latter should be preferred to augmentation techniques and standard-length implants due to fewer complications, lower morbidity and greater comfort for patients. |
|  |  |  |  |  | Standard implants (> 8 mm) |  | complications rate | RR = 0.17 (0.04 - 0.73) |  |
|  |  |  |  |  |  |  | Post-operative complications | RR = 0.22 (0.07 - 0.71) |  |
| Uehara et al. (43) | 2018 | SR with MA | RCTs | 7/7 | Short implants (≤ 7 mm) | Atrophic posterior maxilla and/or mandible / Bone augmentation | Survival rate | RD = -0.02 (-0.04 - 0.00) | The placement of short implants is a predictable alternative for the rehabilitation of atrophic posterior regions, avoiding all the intrinsic disadvantages of bone augmentation procedures. |
|  |  |  |  |  | Standard implants (> 7 mm) |  | Marginal bone loss | MD = -0.13 (-0.22 - -0.05) |  |
| Palacios et al. (44) | 2018 | SR with MA | RCTs | 8/8 | Short implants (< 10 mm) | Atrophic maxilla or mandible / Bone augmentation | Survival rate | RR = 1.34 (0.63 - 2.87) | There are no differences between the placement of short or standard implants in the treatment of atrophic arches. |
|  |  |  |  |  | Standard implants (≥ 10 mm) |  | Marginal bone loss | MD = -0.04 (-0.10 - 0.02) |  |
|  |  |  |  |  |  |  | Prosthetic complications | RR = 0.90 (0.38 - 2.14) |  |
| Papaspyridakos et al. (45) | 2018 | SR with MA | RCTs | 10/10 | Short implants (≤ 6 mm) | Atrophic posterior maxilla and/or mandible / NR | Survival rate | RR = 1.29 (0.67 - 2.50) | Short implants were found to have higher variability and lower predictability in survival rates compared to standard implants after periods of 1-5 years in function. |
|  |  |  |  |  | Standard implants (> 6 mm) |  |  |  |  |
| Starch-Jensen et al. (46) | 2018 | SR | RCTs | 6/0 | Short implants (≤ 8 mm) | Atrophic posterior mandible / Bone augmentation | Survival rate | SI: 91.7%; ST: 95.1% | Short implants and the sandwich osteotomy with delayed placement of standard length implants appear to result in predictable outcomes in terms of high survival rate of suprastructures and implants after prosthetic rehabilitation of the partially edentulous atrophic posterior mandible. |
|  |  |  |  |  | Standard implants (> 8 mm) |  | Marginal bone loss | SI: -2.24 mm; ST: -3.01 mm |  |
| Cruz et al. (47) | 2018 | SR with MA | RCTs | 11/11 | Short implants (≤ 8.5 mm) | Atrophic posterior maxilla / Sinus floor elevation | Survival rate | RR = 1.08 (0.46 - 2.52) | Short implant placement is an effective alternative because of fewer biological complications and similar survival and marginal bone loss than long implant placement with maxillary sinus augmentation. |
|  |  |  |  |  |  |  | Marginal bone loss | MD = -0.05 (-0.10 - 0.01) |  |
|  |  |  |  |  | Standard implants (> 8.5 mm) |  | Biological complications | RR = 0.21 (0.10 - 0.41) |  |
|  |  |  |  |  |  |  | Prosthetic complications | RR = 3.15 (1.32 - 7.51) |  |
| de Souza et al. (48) | 2018 | SR with MA | RCTs and CCTs | 4/3 | Short implants (≤ 8 mm) | Posterior maxilla and/or mandible / Bone augmentation | Survival rate | RR = 1.00 (0.97 - 1.03) | The survival rate of short implants was similar to the standard ones in posterior single crowns, for the one-year follow-up period. |
|  |  |  |  |  | Standard implants (> 8 mm) |  |  |  |  |
| Fan et al. (49) | 2017 | SR with MA | RCTs | 7/7 | Short implants (≤ 8 mm) | Atrophic posterior maxilla / Sinus floor elevation | Survival rate | RR = 1.00 (0.97 - 1.03) | No difference between the survival rates of short implants and long implants; complications in short implants are lower than that in long implants. |
|  |  |  |  |  | Standard implants (> 8 mm) |  | complications rate | RR = 0.58 (0.37 - 0.90) |  |
| Tong et al. (50) | 2017 | SR with MA | RCTs | 9/9 | Short implants (≤ 8 mm) | Partial or total maxillary and/or mandibular edentulism / Bone augmentation | Implant failure | RR = 0.78 (0.10 - 5.16) | Short implants are considered to be a suitable alternative treatment when bone height is not adequate for standard implants. |
|  |  |  |  |  |  |  | Marginal bone loss | MD = -0.57 (-1.10 - -0.04) |  |
|  |  |  |  |  | Standard implants (> 8 mm) |  | Prosthetic complications | RR = 0.91 (0.31 - 2.62) |  |
|  |  |  |  |  |  |  | complications rate | RR = 0.34 (0.15 - 0.79) |  |
| Toti et al. (51) | 2017 | SR with MA | RCTs | 12/5 | Short implants (≤ 8 mm) | Atrophic posterior mandible / Bone augmentation | Implant failure | RR = 1.59 (0.54 - 4.69) | Short implants placed in the posterior atrophic areas of partially edentulous mandibles were associated with superior outcomes compared with long implants in augmented bone, such as lower rate of biological complications and of peri-implant bone loss. |
|  |  |  |  |  |  |  | Marginal bone loss | MD = 0.05 (0.03 - 0.08) |  |
|  |  |  |  |  | Standard implants (> 8 mm) |  | Biological complications | RR = 2.82 (1.81 - 4.40) |  |
|  |  |  |  |  |  |  | Prosthetic complications | RR = 1.45 (0.56 - 3.96) |  |
| Lemos et al. (52) | 2016 | SR with MA | RCTs and PSs | 13/13 | Short implants (≤ 8 mm) | Posterior maxilla and/or mandible / NR | Survival rate | RR = 1.35 (0.82 - 2.22) | Short implants are considered a predictable treatment for posterior jaws. However, short implants with length less than 8 mm (4-7 mm) should be used with caution because they present greater risks to failures compared to standard implants. |
|  |  |  |  |  |  |  | Marginal bone loss | MD = -0.20 (-0.41 - 0.00) |  |
|  |  |  |  |  | Standard implants (> 8 mm) |  | Prosthetic complications | RR = 0.96 (0.44 - 2.09) |  |
|  |  |  |  |  |  |  | complications rate | RR = 0.54 (0.27 - 1.09) |  |
| Camps-Font et al. (53) | 2016 | SR with MA | RCTs | 14/4 | Short implants (≤ 8 mm) | Atrophic posterior mandible / Bone augmentation | Implant failure | OR = 1.02 (0.31 - 3.31) | Short implants in the posterior area of the mandible seem to be preferable to vertical augmentation procedures, which present similar implant and prosthetic failure rates but greater morbidity. |
|  |  |  |  |  | Standard implants (> 8 mm) |  | Prosthetic complications | OR = 0.64 (0.21 - 1.96) |  |
|  |  |  |  |  |  |  | Post-operative complications | OR = 0.12 (0.05 - 0.26) |  |
| Thoma et al. (54) |  |  | RCTs | 8/0 | Short implants (≤ 8 mm) | Atrophic posterior maxilla / Sinus floor elevation | Survival rate | SI: 99%; ST: 99.5% | Implant survival rates are high for short implants and standard implants placed in the augmented sinus and their respective reconstructions. Given the greater number of biological complications, increased morbidity, costs, and surgical time of standard dental implants in the augmented sinus, shorter dental implants may represent the preferred treatment alternative. |
|  |  |  |  |  |  |  | Marginal bone loss | SI: -0.1 to -1.02 mm; ST: -0.1 to -1.15 mm |  |
|  |  |  |  |  | Standard implants (> 8 mm) |  | Biological complications | SI: 2.94%; ST: 8.84% |  |
|  |  |  |  |  |  |  | Prosthetic complications | SI: 1.98%;ST: 1.4% |  |
| Nisand et al. (55) | 2015 | SR | RCTs | 4/0 | Short implants (≤ 8 mm) | Atrophic posterior mandible / Bone augmentation | Survival rate | SI: 96.24%; ST: 95.09% | Implant and prosthetic survival rates are similar between short implants and standard implants placed in vertically augmented bone. |
|  |  |  |  |  |  |  | Marginal bone loss | SI: -1.23 mm; ST: -1.51 mm |  |
|  |  |  |  |  | Standard implants (> 8 mm) |  | Prosthetic complications | SI: 3.68%; ST: 5.45% |  |
|  |  |  |  |  |  |  | complications rate | SI: 14.11%; ST: 38.79% |  |
| Lee et al. (56) | 2014 | SR with MA | RCTs | 4/4 | Short implants (≤ 8 mm) | Atrophic posterior mandible / Bone augmentation | Survival rate | RR = 0.68 (0.24 - 1.93) | Placement of short dental implants could be a predictable alternative to longer implants to reduce surgical complications and patient morbidity in situations where vertical augmentation procedures are needed. |
|  |  |  |  |  | Standard implants (> 8 mm) |  | Biological complications | RR = 0.27 (0.04 - 2.08) |  |
|  |  |  |  |  |  |  | Prosthetic complications | RR = 0.92 (0.43 - 1.97) |  |
| Monje et al. (57) | 2014 | SR with MA | RCTs and CCTs | 5/2 | Short implants (< 10 mm) | Partial or total maxillary and/or mandibular edentulism / NR | Marginal bone loss | SMD = -0.32 (-0.83 - 0.18) | Short dental implants had peri-implant marginal bone loss similar to standard implants for implant-supported fixed prostheses. |
|  |  |  |  |  | Standard implants (≥ 10 mm) |  |  |  |  |
| Mezzomo et al. (58) | 2014 | SR with MA | CCTs, Cohorts and Case series | 21/16 | Short implants (< 10 mm) | Posterior maxilla and/or mandible / Bone augmentation | Implant failure | RD = 0.06 (0.04 - 0.10) | Single crowns supported by short implants in the posterior region are a predictable treatment option with reduced failure rates, biological/prosthetic complications and minimal bone loss. |
|  |  |  |  |  |  |  | Marginal bone loss | MD = 0.84 (0.55 - 1.13) |  |
|  |  |  |  |  | Standard implants (≥ 10 mm) |  | Biological complications | RD = 0.04 (0.02 - 0.08) |  |
|  |  |  |  |  |  |  | Prosthetic complications | RD = 0.03 (0.02 - 0.06) |  |
| Monje et al. (59) | 2013 | SR with MA | CCTs | 13/13 | Short implants (< 10 mm) | Partial maxillary and/or mandibular edentulism / NR | Survival rate | RR = 0.92 (0.88 - 0.94) | In the long term, short implants are as predictable as standard implants. |
|  |  |  |  |  | Standard implants (≥ 10 mm) |  |  |  |  |
| Kotsovilis et al. (60) | 2009 | SR with MA | PSs | 22/17 | Short implants (< 10 mm) | Partial maxillary and/or mandibular edentulism / NR | Survival rate | RR = 0.99 (0.98 - 1.00) | The placement of short rough-surface implants is not a less efficacious treatment modality compared to the placement of standard rough-surface implants for the replacement of missing teeth in either totally or partially edentulous patients. |
|  |  |  |  |  | Standard implants (≥ 10 mm) |  |  |  |  |

SR = Systematic review; MA = Meta-analysis; RCT = Randomized clinical trial; CCT = Controlled clinical trial; RR = Risk ratio; MD = Mean difference; OR = Odds ratio; RD = Risk difference; OS = Observational study; RS = Retrospective study; PS = Prospective study; SI = Short implant; ST = Standard implant; NR = Not Reported

**References**

1. Alemán BO, Rivera-Velazquez I, Jana-Hernández Z, Rivas-Tumanyan S, Guerrero-Rodríguez LM, Elias-Boneta AR. Long-Term Outcomes of Short versus Long Dental Implants with Sinus Lift in Atrophied Posterior Maxillae: A Systematic Review and Meta-Analysis. *P R Health Sci J*. 2025;44(1):54-62.

2. Abayov P, Sarikov R, Nazarenko LM, Babich O, Haimov E, Juodzbalys G. Outcome Difference between Short and Longer Dental Implants Placed Simultaneously with Alveolar Bone Augmentation: a Systematic Review and Meta-Analysis. *J Oral Maxillofac Res.* 2024 ;15(2):e2. doi:10.5037/jomr.2024.15202

3. Zhang Y, Tang X, Zhang Y, Cao C. A network meta-analysis comparing treatment modalities of short and long implants in the posterior maxilla with insufficient bone height. *BMC Oral Health*. 2024;24(1). doi:10.1186/s12903-024-05377-1

4. Emfietzoglou R, Dereka X. Survival Rates of Short Dental Implants (≤6 mm) Used as an Alternative to Longer (>6 mm) Implants for the Rehabilitation of Posterior Partial Edentulism: A Systematic Review of RCTs. *Dent J*. 2024;12(6). doi:10.3390/dj12060185

5. Liang L, Wu X, Yan Q, Shi B. Are short implants (≤8.5 mm) reliable in the rehabilitation of completely edentulous patients: A systematic review and meta-analysis. *J Prosthet Dent*. 2024;131(5):826-32. doi: 0.1186/s12903-024-05377-1

6. Kermanshah H, Keshtkar A, Hassani A, Bitaraf T. Comparing short implants to standard dental implants: a systematic review and meta-analysis of randomized controlled trials with extended follow-up. *Evid Based Den*. 2023;24(4):192-3. doi:10.1038/s41432-023-00924-1

7. Mester A, Onisor F, Stasio DD, Piciu A, Cosma AM, Bran S. Short Implants versus Standard Implants and Sinus Floor Elevation in Atrophic Posterior Maxilla: A Systematic Review and Meta-Analysis of Randomized Clinical Trials with ≥5 Years’ Follow-Up. *J Pers Med*. 2023;13(2):169. doi:10.3390/jpm13020169.

8. Rosa A, nueva E a sitio externo E enlace se abrirá en una ventana, Pujia AM, Arcuri C. Complete Full Arch Supported by Short Implant (<8 mm) in Edentulous Jaw: A Systematic Review. *Appl Sci.* 2023;13(12):7162. doi: 10.3390/app13127162.

9. Grunau O, Terheyden H. Lateral augmentation of the sinus floor followed by regular implants versus short implants in the vertically deficient posterior maxilla: a systematic review and timewise meta-analysis of randomized studies. *Int J Oral Maxillofac Surg*. 2023;52(7):813-24. doi: 10.1016/j.ijom.2022.11.015

10. 启航张, 佳明龚, 佳颖余, 瑞敏赵, 萍苟, 占海余. 在后牙区应用4 mm超短种植体临床效果的Meta分析. *West China J Stomatol.* 2023;41(1):80. doi: 10.7518/hxkq.2023.01.011

11. Liang L, Wu X, Yan Q, Shi B. Are short implants (≤8.5 mm) reliable in the rehabilitation of completely edentulous patients: A systematic review and meta-analysis. *J Prosthet Dent*. 2022;S0022-3913(22):140-8. doi: 10.1016/j.prosdent.2022.02.015

12. Wang M, Liu F, Ulm C, Shen H, Rausch-Fan X. Short Implants versus Longer Implants with Sinus Floor Elevation: A Systemic Review and Meta-Analysis of Randomized Controlled Trials with a Post-Loading Follow-Up Duration of 5 Years. *Materials.* 2022;15(13):4722. doi:10.3390/ma15134722

13. Medikeri RS, Pereira MA, Waingade M, Navale S. Survival of surface-modified short versus long implants in complete or partially edentulous patients with a follow-up of 1 year or more: a systematic review and meta-analysis. *J Periodontal Implant Sci*. 2022;52(2):261-81. doi:10.5051/jpis.2007340367

14. Toledano M, Fernández-Romero E, Vallecillo C, Toledano R, Osorio MT, Vallecillo-Rivas M. Short versus standard implants at sinus augmented sites: a systematic review and meta-analysis. *Clin Oral Investig*. 2022;26(11):6681-98. doi:10.1007/s00784-022-04628-1

15. Guida L, Bressan E, Cecoro G, Volpe AD, Fabbro MD, Annunziata M. Short versus Longer Implants in Sites without the Need for Bone Augmentation: A Systematic Review and Meta-Analysis of Randomized Controlled Trials. *Materials.* 2022;15(9). doi:10.3390/ma15093138

16. Tang C, Du Q, Luo J, Peng L. Simultaneous placement of short implants (≤ 8 mm) versus standard length implants (≥ 10 mm) after sinus floor elevation in atrophic posterior maxillae: a systematic review and meta-analysis. *Int J Implant Dent.* 2022;8(1):45. doi:10.1186/s40729-022-00443-1

17. Terheyden H, Meijer GJ, Raghoebar GM. Vertical bone augmentation and regular implants versus short implants in the vertically deficient posterior mandible: a systematic review and meta-analysis of randomized studies. *Int J Oral Maxillofac Surg.* 2021;50(9):1249-58. doi: 10.1016/j.ijom.2021.01.005

18. Moraschini V, Mourão CF de AB, Montemezzi P, Kischinhevsky ICC, de Almeida DCF, Javid K, et al. Clinical Comparation of Extra-Short (4 mm) and Long (>8 mm) Dental Implants Placed in Mandibular Bone: A Systematic Review and Metanalysis. *Healthc Basel Switz*. 2021;9(3):315. doi:10.3390/healthcare9030315

19. Bitinas D, Bardijevskyt G. Short implants without bone augmentation vs. long implants with bone augmentation: systematic review and meta-analysis. *Aust Dent J.* 2021;66(S1):S71-81. doi:10.1111/adj.12859

20. Yu X, Ruogu X, Zhengchuan Z, Yang Y, Feilong D. A meta-analysis indicating extra-short implants (≤ 6 mm) as an alternative to longer implants (≥ 8 mm) with bone augmentation. *Sci Rep Nat Publ Group.* 2021;11(1):8152. doi:10.1038/s41598-021-87507-1

21. Wu H, Shi Q, Huang Y, Chang P, Huo N, Jiang Y, et al. Failure Risk of Short Dental Implants Under Immediate Loading: A Meta-Analysis. *J Prosthodont*. 2021;30(7):569-80. doi:10.1111/jopr.13376

22. Chaware S, Thakare V, Chaudhary R, Jankar A, Thakkar S, Borse S. The rehabilitation of posterior atrophic maxilla by using the graftless option of short implant versus conventional long implant with sinus graft: A systematic review and meta-analysis of randomized controlled clinical trial. *J Indian Prosthodont Soc.* 2021;21(1):28-44. doi: 10.4103/jips.jips_400_20

23. Abdel-Halim M, Issa D, Chrcanovic BR, nueva E a sitio externo E enlace se abrirá en una ventana. The Impact of Dental Implant Length on Failure Rates: A Systematic Review and Meta-Analysis. *Materials.* 2021;14(14):3972. doi:10.3390/ma14143972

24. Carosi P, Lorenzi C, Lio F, Laureti M, Ferrigno N, Arcuri C. Short implants (≤6mm) as an alternative treatment option to maxillary sinus lift. *Int J Oral Maxillofac Surg.* 2021;50(11):1502-10. doi: 10.1016/j.ijom.2021.02.014

25. Carosi P, Lorenzi C, Laureti M, Ferrigno N, Arcuri C. Short Dental Implants (≤ 6 mm) to Rehabilitate Severe Mandibular Atrophy: A Systematic Review. *Int J Oral Maxillofac Implants.* 2021;36(1):30-7. doi:10.11607/jomi.8510

26. Xu X, Huang J, Fu X, Kuang Y, Yue H, Song J, et al. Short implants versus longer implants in the posterior alveolar region after an observation period of at least five years: A systematic review and meta-analysis. *J Dent*. 2020;100:103386. doi: 10.1016/j.jdent.2020.103386

27. Lozano-Carrascal N, Anglada-Bosqued A, Salomó-Coll O, Hernández-Alfaro F, Wang HL, Gargallo-Albiol J. Short implants (<8mm) versus longer implants (≥8mm) with lateral sinus floor augmentation in posterior atrophic maxilla: A meta-analysis of RCT`s in humans. *Med Oral Patol Oral Cirugia Bucal.* 2020;25(2):e168-79. doi: 10.4317/medoral.23248

28. Xu X, Hu B, Xu Y, Liu Q, Ding H, Xu L. Short versus standard implants for single-crown restorations in the posterior region: A systematic review and meta-analysis. J Prosthet Dent. 2020;124(5):530-8. doi: 10.1016/j.prosdent.2019.09.030

29. Iezzi G, Perrotti V, Felice P, Barausse C, Piattelli A, Del Fabbro M. Are <7-mm long implants in native bone as effective as longer implants in augmented bone for the rehabilitation of posterior atrophic jaws? A systematic review and meta-analysis. *Clin Implant Dent Relat Res*. 2020;22(5):552-66. doi:10.1111/cid.12946

30. Vazouras K, de Souza AB, Gholami H, Papaspyridakos P, Pagni S, Weber HP. Effect of time in function on the predictability of short dental implants (≤6 mm): A meta-analysis. *J Oral Rehabil.* 2020;47(3):403-15. doi:10.1111/joor.12925

31. Mokcheh A, Jegham H, Turki S. Short implants as an alternative to sinus lift for the rehabilitation of posterior maxillary atrophies: Systematic review and meta-analysis. *J Stomatol Oral Maxillofac Surg*. 2019;120(1):28-37. doi:10.1016/j.jormas.2018.11.006

32. Nielsen HB, Schou S, Isidor F, Christensen AE, Starch-Jensen T. Short implants (≤8mm) compared to standard length implants (>8mm) in conjunction with maxillary sinus floor augmentation: a systematic review and meta-analysis. *Int J Oral Maxillofac Surg*. 2019;48(2):239-49. doi:10.1016/j.ijom.2018.05.010

33. Altaib FH, Alqutaibi AY, Al-Fahd A, Eid S. Short dental implant as alternative to long implant with bone augmentation of the atrophic posterior ridge: A systematic review and meta-analysis of RCTs. *Quintessence Int.* 2019;50(8):636-51. doi:10.3290/j.qi.a42948

34. Ravidà A, Wang IC, Sammartino G, Barootchi S, Tattan M, Troiano G, et al. Prosthetic Rehabilitation of the Posterior Atrophic Maxilla, Short (≤6 mm) or Long (≥10 mm) Dental Implants? A Systematic Review, Meta-analysis, and Trial Sequential Analysis: Naples Consensus Report Working Group A. Implant Dent. 2019;28(6):590-602. doi:10.1097/ID.0000000000000919

35. Esposito M, Buti J, Barausse C, Gasparro R, Sammartino G, Felice P. Short implants versus longer implants in vertically augmented atrophic mandibles: A systematic review of randomised controlled trials with a 5-year post-loading follow-up. Int J Oral Implantol. 2019;12(3):267-80.

36. Amine M, Guelzim Y, Benfaida S, Bennani A, Andoh A. Short implants (5–8 mm) vs. long implants in augmented bone and their impact on peri-implant bone in maxilla and/or mandible: Systematic review. J Stomatol Oral Maxillofac Surg. 2019;120(2):133-42. doi:10.1016/j.jormas.2018.11.007

37. Chen S, Ou Q, Wang Y, Lin X. Short implants (5‐8 mm) vs long implants (≥10 mm) with augmentation in atrophic posterior jaws: A meta‐analysis of randomised controlled trials. *J Oral Rehabil.* 2019;46(12):1192-203. doi:10.1111/joor.12860

38. Bitaraf T, Keshtkar A, Rokn AR, Monzavi A, Geramy A, Hashemi K. Comparing short dental implant and standard dental implant in terms of marginal bone level changes: A systematic review and meta-analysis of randomized controlled trials. *Clin Implant Dent Relat Res*. 2019;21(4):796-812. doi:10.1111/cid.12774

39. Ravidà A, Wang IC, Barootchi S, Askar H, Tavelli L, Gargallo-Albiol J, et al. Meta-analysis of randomized clinical trials comparing clinical and patient-reported outcomes between extra-short (≤6 mm) and longer (≥10 mm) implants. *J Clin Periodontol.* 2019;46(1):118-42. doi:10.1111/jcpe.13026

40. Yan Q, Wu X, Su M, Hua F, Shi B. Short implants (≤6 mm) versus longer implants with sinus floor elevation in atrophic posterior maxilla: A systematic review and meta-analysis. *BMJ Open*. 2019;9(10). doi:10.1136/bmjopen-2019-029826

41. Aldawood T, Qarni M, Alhayek A, Muslih W, Alfantoukh A, Albeladi R, et al. Comparison between short dental implants versus standard dental implants of posterior jaws: A systematic review & meta-analysis. World J Pharm Res. 2019;8:1501-13. doi: 10.20959/wjpr201912-16180

42. de N Dias FJ, Pecorari VGA, Martins CB, Del Fabbro M, Casati MZ. Short implants versus bone augmentation in combination with standard-length implants in posterior atrophic partially edentulous mandibles: systematic review and meta-analysis with the Bayesian approach. *Int J Oral Maxillofac Surg.* 2019;48(1):90-6. doi: 10.1016/j.ijom.2018.05.009

43. Uehara PN, Matsubara VH, Igai F, Sesma N, Mukai MK, Araujo MG. Short dental implants (≤7mm) versus longer implants in augmented bone area: A meta-analysis of randomized controlled trials. *Open Dent J.* 2018;12(1):354-65. doi: 10.2174/1874210601812010354

44. Palacios JAV, Garcia JJ, Caramês JMM, Quirynen M, da Silva Marques DN. Short implants versus bone grafting and standard-length implants placement: a systematic review. *Clin Oral Investig.* 2018;22(1):69-80. doi:10.1007/s00784-017-2205-0

45. Papaspyridakos P, De Souza A, Vazouras K, Gholami H, Pagni S, Weber HP. Survival rates of short dental implants (≤6 mm) compared with implants longer than 6 mm in posterior jaw areas: A meta‐analysis. *Clin Oral Implants Res*. 2018;29(S16):8-20. doi:10.1111/clr.13289

46. Starch-Jensen T, Nielsen HB. Prosthetic Rehabilitation of the Partially Edentulous Atrophic Posterior Mandible with Short Implants (≤ 8 mm) Compared with the Sandwich Osteotomy and Delayed Placement of Standard Length Implants (> 8 mm): a Systematic Review. *J Oral Maxillofac Res.* 2018;9(2):e2. doi:10.5037/jomr.2018.9202

47. Cruz RS, de Araújo Lemos CA, de Souza Batista VE, e Oliveira HFF, de Luna Gomes JM, Pellizzer EP, et al. Short implants versus longer implants with maxillary sinus lift. A systematic review and meta-analysis. *Braz Oral Res.* 2018;32:e86. doi:10.1590/1807-3107bor-2018.vol32.0086

48. Tolentino da Rosa de Souza P, Binhame Albini Martini M, Reis Azevedo-Alanis L. Do short implants have similar survival rates compared to standard implants in posterior single crown?: A systematic review and meta-analysis. *Clin Implant Dent Relat Res*. 2018;20(5):890-901. doi:10.1111/cid.12634

49. Fan T, Li Y, Deng WW, Wu T, Zhang W. Short Implants (5 to 8 mm) Versus Longer Implants (>8 mm) with Sinus Lifting in Atrophic Posterior Maxilla: A Meta-Analysis of RCTs. *Clin Implant Dent Relat Res.* 2017;19(1):207-15. doi:10.1111/cid.12432

50. Tong Q, Zhang X, Yu L. Meta-analysis of randomized controlled trials comparing clinical outcomes between short implants and long implants with bone augmentation procedure. *Int J Oral Maxillofac Implants.* 2017;32(1):e25-34. doi:10.11607/jomi.4793

51. Toti P, Marchionni S, Menchini-Fabris GB, Marconcini S, Covani U, Barone A. Surgical techniques used in the rehabilitation of partially edentulous patients with atrophic posterior mandibles: A systematic review and meta-analysis of randomized controlled clinical trials. *J Cranio-Maxillo-fac Surg Off Publ Eur Assoc Cranio-Maxillo-fac Surg.* 2017;45(8):1236-45. doi:10.1016/j.jcms.2017.04.011

52. Lemos CAA, Ferro-Alves ML, Okamoto R, Mendonça MR, Pellizzer EP. Short dental implants versus standard dental implants placed in the posterior jaws: A systematic review and meta-analysis. *J Dent.* 2016;47:8-17. doi:10.1016/j.jdent.2016.01.005

53. Camps-Font O, Burgueño-Barris G, Figueiredo R, Jung RE, Gay-Escoda C, Valmaseda-Castellón E. Interventions for Dental Implant Placement in Atrophic Edentulous Mandibles: Vertical Bone Augmentation and Alternative Treatments. A Meta-Analysis of Randomized Clinical Trials. *J Periodontol.* 2016;87(12):1444-57. doi:10.1902/jop.2016.160226

54. Thoma DS, Zeltner M, Hüsler J, Hämmerle CHF, Jung RE. EAO Supplement Working Group 4 - EAO CC 2015 Short implants versus sinus lifting with longer implants to restore the posterior maxilla: A systematic review. *Clin Oral Implants Res*. 2015; 26:154-69. doi:10.1111/clr.12615

55. Nisand D, Picard N, Rocchietta I. Short implants compared to implants in vertically augmented bone: a systematic review. *Clin Oral Implants Res.* 2015;26(S11):170-9. doi:10.1111/clr.12632

56. Lee SA, Lee CT, Fu MM, Elmisalati W, Chuang SK. Systematic review and meta-analysis of randomized controlled trials for the management of limited vertical height in the posterior region: Short implants (5 to 8 mm) vs longer implants (> 8 mm) in vertically augmented sites. *Int J Oral Maxillofac Implants.* 2014;29(5):1085-97. doi:10.11607/jomi.3504

57. Monje A, Suarez F, Galindo-Moreno P, García-Nogales A, Fu JH, Wang HL. A systematic review on marginal bone loss around short dental implants (<10 mm) for implant-supported fixed prostheses. *Clin Oral Implants Res*. 2014;25(10):1119-24. doi:10.1111/clr.12236

58. Mezzomo LA, Miller R, Triches D, Alonso F, Shinkai RSA. Meta-analysis of single crowns supported by short (<10 mm) implants in the posterior region. *J Clin Periodontol*. 2014;41(2):191-213. doi:10.1111/jcpe.12180

59. Monje A, Chan HL, Fu JH, Suarez F, Galindo-Moreno P, Wang HL. Are short dental implants (<10 mm) effective? A meta-analysis on prospective clinical trials. *J Periodontol*. 2013;84(7):895-904. doi:10.1902/jop.2012.120328

60. Kotsovilis S, Fourmousis I, Karoussis IK, Bamia C. A systematic review and meta-analysis on the effect of implant length on the survival of rough-surface dental implants. *J Periodontol.* 2009;80(11):1700-18. doi:10.1902/jop.2009.090107
